# Tracking *in situ* checkpoint inhibitor-bound target T cells in patients with checkpoint-induced colitis

**DOI:** 10.1016/j.ccell.2024.04.010

**Published:** 2024-05-13

**Authors:** Tarun Gupta, Agne Antanaviciute, Chloe Hyun-Jung Lee, Rosana Ottakandathil Babu, Anna Aulicino, Zoe Christoforidou, Paulina Siejka-Zielinska, Caitlin O’Brien-Ball, Hannah Chen, David Fawkner-Corbett, Ana Sousa Geros, Esther Bridges, Colleen McGregor, Nicole Cianci, Eve Fryer, Nasullah Khalid Alham, Marta Jagielowicz, Ana Mafalda Santos, Martin Fellermeyer, Simon J. Davis, Kaushal Parikh, Vincent Cheung, Lulia Al-Hillawi, Sarah Sasson, Stephanie Slevin, Oliver Brain, TIP Consortium, Elizabeth Bird-Lieberman, Simona Fourie, Richard Johnston, Heman Joshi, Debabrata Mujamdar, Simon Panter, Nishant Patodi, Sebastian Shaji, Jude Tidbury, Ajay Verma, Ricardo A. Fernandes, Hashem Koohy, Alison Simmons

**Affiliations:** 1 Medical Research Council (MRC) Translational Immune Discovery Unit, MRC Weatherall Institute of Molecular Medicine (WIMM), Radcliffe Department of Medicine, John Radcliffe Hospital, University of Oxford, Oxford OX3 9DS, UK; 2 Translational Gastroenterology Unit, John Radcliffe Hospital, Oxford OX3 9DU, UK; 3 MRC WIMM Centre For Computational Biology, MRC Weatherall Institute of Molecular Medicine, Radcliffe Department of Medicine, John Radcliffe Hospital, University of Oxford, Oxford OX3 9DS, UK; 4 Chinese Academy of Medical Sciences (CAMS) Oxford Institute (COI), University of Oxford, Oxford OX3 7BN, UK; 5 Academic Paediatric Surgery Unit (APSU), Nuffield Department of Surgical Sciences, University of Oxford, Oxford OX3 9DU, UK; 6 Pathology, Department of Cellular Pathology, Oxford University Hospitals NHS Foundation Trust, Oxford OX3 9DU, UK; 7 Nuffield Department of Surgical Sciences and Oxford NIHR Biomedical Research Centre (BRC), University of Oxford, John Radcliffe Hospital, Oxford OX3 9DU, UK; 8 Radcliffe Department of Medicine, John Radcliffe Hospital, University of Oxford, Oxford OX3 9DU, UK

**Keywords:** cancer immunotherapy, checkpoint colitis, ulcerative colitis, scRNA-Seq, spatial transcriptomics

## Abstract

The success of checkpoint inhibitors (CPIs) for cancer has been tempered by immune-related adverse effects including colitis. CPI-induced colitis is hallmarked by expansion of resident mucosal IFNγ cytotoxic CD8^+^ T cells, but how these arise is unclear. Here, we track CPI-bound T cells in intestinal tissue using multimodal single-cell and subcellular spatial transcriptomics (ST). Target occupancy was increased in inflamed tissue, with drug-bound T cells located in distinct microdomains distinguished by specific intercellular signaling and transcriptional gradients. CPI-bound cells were largely CD4^+^ T cells, including enrichment in CPI-bound peripheral helper, follicular helper, and regulatory T cells. IFNγ CD8^+^ T cells emerged from both tissue-resident memory (TRM) and peripheral populations, displayed more restricted target occupancy profiles, and co-localized with damaged epithelial microdomains lacking effective regulatory cues. Our multimodal analysis identifies causal pathways and constitutes a resource to inform novel preventive strategies.

## Highlights

• Multi-modal scRNA-seq and ST atlas incorporating CPI-bound T cell tracking
• Identified CD4^+^ (Tfh, Tph, Th17, Tregs) and CD8^+^ (HAVCR2^+^) T cells bound by CPI
• Increased CPI-bound T cells in distinct tissue niches in patients with CPI-colitis
• Colonic cellular microdomain composition differentiates CPI-colitis from UC

## Graphical abstract

**Figure:**
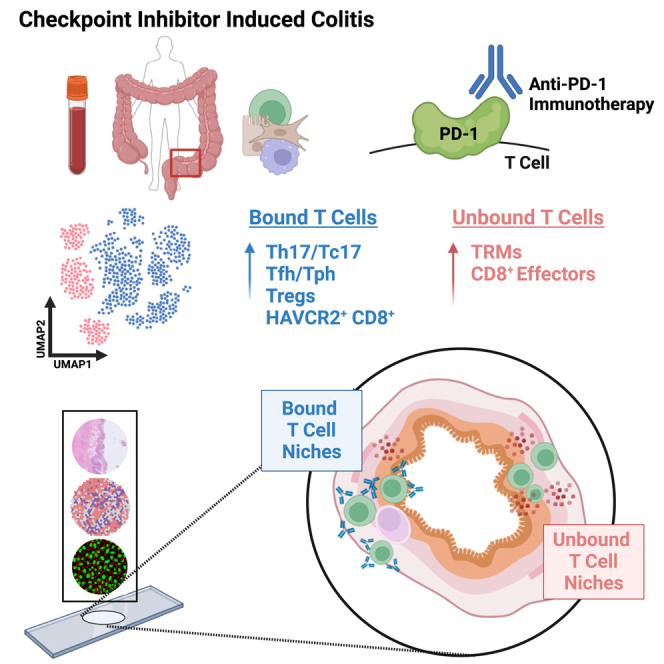

## Teaser

Gupta et al. track checkpoint inhibitor (CPI)-bound T cells utilizing scRNA-seq and subcellular spatial transcriptomics in blood and intestinal tissue, defining distinct T cell subtypes and tissue niches linked to colitis development. They define the pathogenic cellular microdomains differentiating CPI-colitis from ulcerative colitis (UC).

## Introduction

Checkpoint inhibitors (CPIs) have revolutionized cancer treatment by blocking regulatory immune signaling mediated by checkpoint molecules, restoring T cell-mediated eradication of cancer cells. However, these therapies also lead to a series of immune-related adverse events (irAEs) of which gastrointestinal inflammation is most common, affecting up to 60% of patients. While intestinal, skin, and joint irAEs have been linked to improved cancer survival, their debilitating side effects have led to an increasing healthcare burden.^1^ Although the precise immune pathogenesis of irAEs remains incompletely defined, it may be due to dysregulated activation of T cells that react with self-antigens or commensal microbes.^2^

Cytotoxic T lymphocyte antigen-4 (CTLA-4), programmed cell death 1(PD-1), and its ligand PD-L1 are key immune checkpoints targeted by CPIs whose expression in T cells increases with activation. CTLA-4 competes with CD28 for the co-stimulatory ligands CD80 and CD86 whereas PD-1 binds to its ligands PD-L1 and PD-L2 to conduct inhibitory T cell receptor (TCR) signaling. These effects of CTLA-4 and PD-1 suppress T cell-mediated cancer killing capacity by facilitating T cell exhaustion.^2^ Anti CLTA-4 and PD-1 restore the ability of T cells to respond to tumor antigen by reversing this effect and CPI-mediated irAEs are thought to be driven by the same principles.

Previous studies explored the etiology of CPI-induced colitis via single-cell RNA sequencing (scRNA-seq) of CD45^+^ intestinal immune cell infiltrates. This revealed accumulation of cycling, cytotoxic CD8^+^ T cells that emerged from tissue-resident memory (TRM) CD8^+^ T cells together with increased CD4^+^ effector and regulatory T cell (Treg) signatures.^3^^,^^4^

To date, it is unclear how CPI-mediated pathogenic CD8^+^ T cell infiltrates arise and whether they are driven via direct CPI binding to tissue-localized CD8^+^ T cells or are a bystander event. It is also not clear why toxicities only occur in a proportion of treated patients and whether the quantity of target engagement mediates side effects. Furthermore, the contribution of pre-existing T cell phenotypes or local tissue microenvironmental factors to CPI-mediated colitis has not been described.

Here, using a combination of unbiased and subcellular spatial transcriptomics (ST) analysis together with integrated multimodal single-cell data derived from immune cells and their surrounding niche, we tracked directly engaged CPI-bound T cells in inflamed and non-inflamed tissue from patients receiving CPI. We define transcriptional microdomains associated with cellular damage and dissociate these from target-bound niches thus providing a mechanistic understanding of how CPI-induced colitis arises.

## Results

### Unbiased scRNA-seq and spatial atlas of CPI-induced colitis

To perform unbiased analysis of CPI-induced colitis, we generated a multi-modal (scRNA-seq, VDJ-seq, CITE-seq) single-cell reference atlas of matched colonic tissue and peripheral blood mononuclear cell (PBMC) samples from patients receiving CPI mono-therapy (anti-PD-1, nivolumab or pembrolizumab) and dual-therapy (anti-PD-1 and anti-CTLA-4, ipilimumab) patients that developed colitis, together with healthy controls, CPI-treated patients that did not develop colitis, and non-CPI-treated inflammation controls (ulcerative colitis (UC), paired involved and non-involved tissue). Key characteristics of the patient cohort are summarized in Table S1.

**Figure 1:**
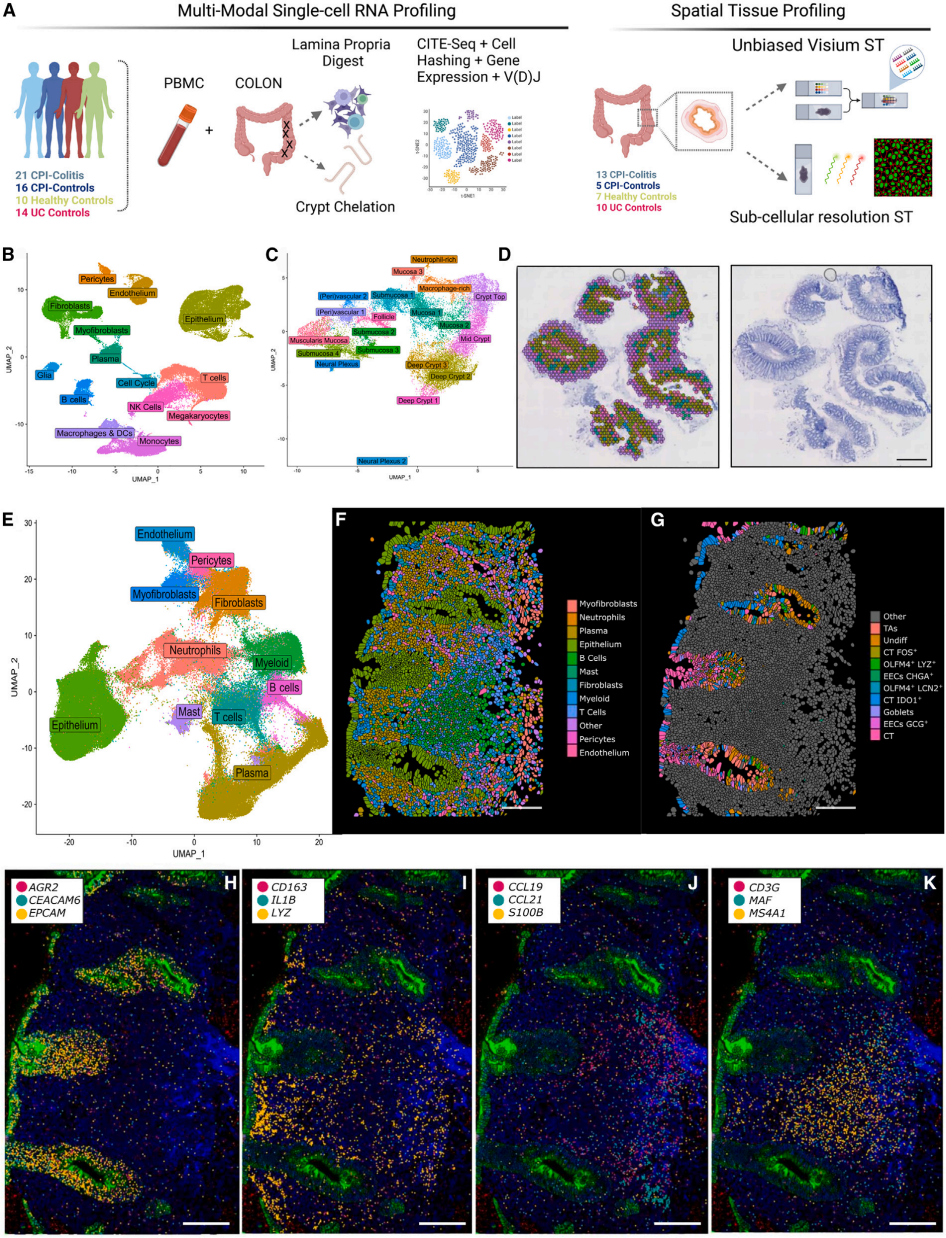
Spatial and single-cell atlas of CPI-Colitis (A) Schematic overview of the experimental design of multi-modal single-cell RNA sequencing and ST. (B) UMAP embedding visualizing scRNA-seq clusters of cell populations obtained from colonic tissue biopsies and paired PBMC samples using all cell isolation strategies. (C) UMAP embedding visualizing spot-based ST spatial region spot clusters, annotated according to tissue location/structure or cell type enrichment. (D) Spatial distribution of transcriptome clusters visualized in C, in a representative ST slide in healthy colon biopsy, overlaid on H&E image. Scale bar, 1 mm. (E) UMAP embedding visualizing segmented cell transcriptome-based clusters from sub-cellular resolution CosMx ST dataset. (F and G) Spatial distribution of cell type (F) and epithelial (G) clusters are visualized on a representative field of view of a CPI-colitis section. Scale bar, 100 μm. (H–K) Spatial distribution of selected gene single mRNA molecule detection visualized over cell morphology stain in a representative field of view of a CPI-colitis section. Segmented single-cell boundaries are shown in black. Selected cell type markers corresponding to epithelial (H), macrophages (I), stromal organizer, lymphatic and glial (J), and T and B cells (K) are shown. Scale bar, 100 μm. See also Figures S1–S3 and Tables S1, S2, and S3.

This single-cell atlas captured 186,838 cells from epithelial, stromal, and immune compartments from 72 donors (Figures 1A, 1B, and S1A–S1E). Clustering analysis identified most cell types expected in the colon (Figure 1B; Tables S2 and S3). In both immune and non-immune lineages, many of these populations were specific to CPI-colitis samples. For instance, we identified CPI-specific “activated” fibroblast populations, similar to cells previously described in Crohn’s disease^5^ as well as phenotypic shifts in epithelium, glia, endothelium, and macrophages (Figures S1F–S1I). These initial findings indicated involvement of the stroma and epithelium and highlighted the need to delineate the contribution of the tissue niche to CPI-induced inflammation.

To address this, we carried out unbiased ST of 18 tissue sections from 16 donors (Figures 1A, 1C, 1D, and S1J–S1L). Integrative clustering analysis of 25,672 tissue-covered ST spots (Figures 1C, 1D, and S1J–S1L; Table S3) revealed 19 joint spatial regions, which we labeled according to anatomical regions or representative cell type enrichment (Figures 1C, 1D, and S2A–S2C). Cell-cell signal correlation analysis (Figure S2D) revealed co-occurrence of cell types that correspond to colonic tissue architecture. In healthy colons, top crypt cells like mature enterocytes and BEST4 cells, and deep crypt cells such as goblets and transit amplifying cells, showed high correlation. Similarly, endothelial, pericyte populations, and S3 peri-vascular fibroblasts were closely associated. Immune cells, like follicular T and B cells, were found together in lymphoid structures. In CPI-colitis, there was notable co-localization of T cell subtypes with epithelial cells and increased proximity of macrophages and T cells (Figures S2D–S2F).

Spot-based ST data offer insights into spatial gene expression and tissue architecture changes but is low resolution, making it difficult to accurately deconvolute direct cell interactions and similar cell states like T cell subtypes. To address these limitations, we conducted subcellular resolution ST on an extra 292,305 cells from 17 samples using CosMx, identifying all key cell populations, including hard-to-capture neutrophils and fine-grained stromal, immune, and epithelial subsets (Figures 1A, 1E, 1F, and S3A–S3C; Table S3). Within the epithelial crypts, transit amplifying epithelia localized to deep crypts; secretory cells were more diffuse while increasingly differentiated enterocytes localized toward crypt tops (Figures 1G–1K). In CPI-colitis and UC, we observed divergent immune infiltrates, including CPI-colitis-specific T cell, neutrophil, and macrophage clusters while plasma cells dominated the tissue landscape in UC. We also observed an increase in “activated” fibroblasts co-localizing in immune-rich niches in both conditions (Figures 1H–1K, S3D, and S3E).

Full ST and scRNA-seq datasets from every cellular compartment have been made available as a community resource via an interactive data portal at https://simmonslab.shinyapps.io/CPI_COLITIS_DATA_PORTAL/. In-depth annotations summarizing cellular phenotypes and CPI-colitis-specific changes are summarized in Table S2.

### Identification of CPI-bound single cells

**Figure 2:**
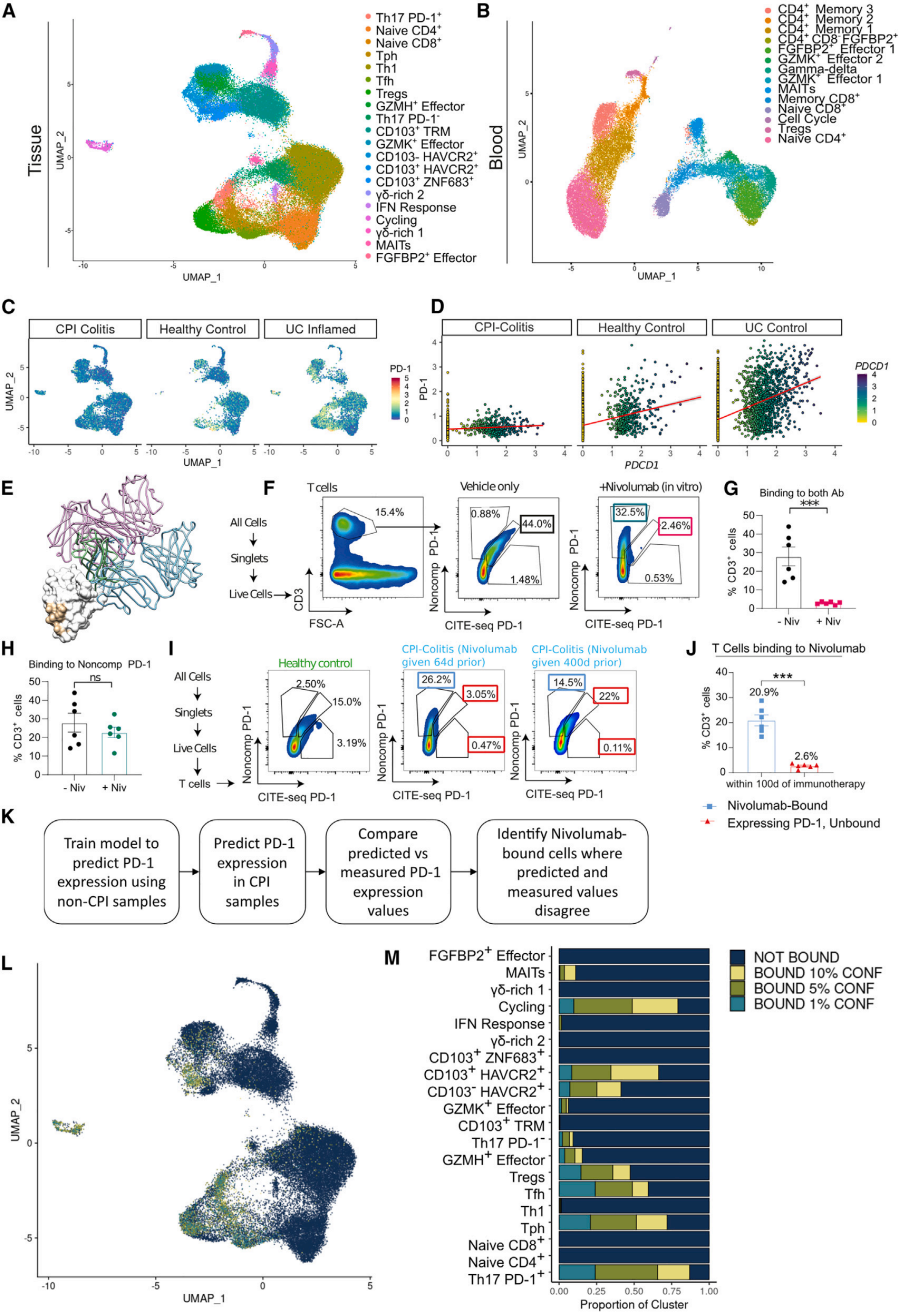
Identification of CPI-bound T cells (A) UMAP embedding visualizes scRNA-seq clusters of T cell subpopulations in CPI-colitis and control samples from colon tissue biopsies. Abbreviations: Tfh, T follicular helper cells; Tph, T peripheral helper cells; Tregs, regulatory T cells; MAIT, mucosal-associated invariant T cells; TRM, tissue-resident memory T cells; IFN response – T cells with strong interferon-induced response signatures. (B) As in A, except PBMC T cells are shown. (C) UMAP visualizes the protein expression distribution of PD-1 in checkpoint-treated (left) and untreated control samples (middle, right). (D) Correlation in expression between *PDCD1* mRNA and PD-1 protein in CPI-treated (left, Pearson’s correlation, r = 0.1486915, *p* = 1.815e−09) and non-treated healthy control (middle, Pearson’s correlation, r = 0.2927159, *p* < 2.2e−16) and UC (right, Pearson’s correlation, r = 0.4765922, *p* < 2.2e−16) sample cells. (E) Crystal structure of hPD-1 (white) in complex with hPD-L1 (green, PDB: 4ZQK), nivolumab (purple, PDB: 5WT9), and pembrolizumab (blue, PDB: 5JXE). The superposition was carried out using the Secondary Structure Matching algorithm in CCP4. (F) FACS strategy for identifying T cells bound to nivolumab after *in vitro* stimulation of healthy colonic biopsies by staining cells with competing and non-competing anti-PD-1 antibodies. (G) Bar plot showing the percentage of T cells positive for both competing and non-competing antibody in nivolumab-treated and untreated cells *in vitro*. Ratio paired t test, mean values are shown, error bars represent standard error of the mean (SEM). *p* value < 0.001∗∗∗; ns = not significant. *n* = 6 samples per condition. (H) Bar plot showing the percentage of T cells positive for non-competing anti-PD-1 antibody in nivolumab-treated and untreated cells *in vitro.* Ratio paired t test, mean values are shown, error bars represent SEM. *p* value < 0.001∗∗∗; ns = not significant. *n* = 6 samples per condition. (I) FACS analysis as in F, except in patient samples *in vivo* in healthy controls (left), CPI-colitis with last nivolumab treatment administered 64 days prior to sample collection (middle) and 400 days prior to sample collection (right). (J) Bar plot showing the percentage of cells in CPI-colitis samples (*n* = 6 per group) that remain bound to nivolumab where immunotherapy was administered within 100 days of sample collection. Paired t test, mean values are shown, error bars represent SEM. *p* value < 0.001∗∗∗; ns = not significant. *n* = 6 samples. (K) Computational strategy to identify CPI-bound single cells using CITE-seq PD-1 expression data. (L) UMAP overlay visualizes the distribution of CPI-bound T cells. (M) Proportion plot visualizing the fraction of each T cell phenotypic cluster that is predicted to remain CPI-bound in CPI-colitis samples. Confidence intervals were calculated from the conditional distribution from quantile random forest model. See also Figure S4 and Tables S2 and S3.

We sequenced a total of 72,561 CD3^+^ cells in colon biopsies and 36,176 from paired PBMCs (Figures 2A and 2B), which enabled T cell clone tracking between the colon and the periphery, as well as characterizing selected protein expression by CITE-seq analysis (Figures S4A–S4E). Following integrated cluster analysis and visualization (Figure 2A; Tables S2 and S3), we identified 20 distinct T cell states in tissue samples and 16 in PBMCs (Figure 2B; Tables S2 and S3), which we subsequently annotated based on cluster protein and mRNA markers, and transcription factor (TF) regulatory networks with reference to previously reported gut datasets (Tables S2 and S3).^3^^,^^6^^,^^7^^,^^8^

CITE-seq analysis showed both reduced anti-PD-1 antibody (clone EH12.2H7) binding as well as reduced correlation between *PDCD1* mRNA and protein detection in most CPI-colitis samples versus non-treated controls (Figures 2C and 2D). We reasoned this observation could be due to either PD-1 downregulation/internalization or retention of nivolumab/pembrolizumab binding, competing with the CITE-seq antibody. Since these treatments target the same PD-1/PD-L1 interaction domain as the CITE-seq antibody, they may hinder its detection of PD-1^9^^,^^10^^,^^11^ (Figure 2E).

To confirm this, using *in vitro* nivolumab-treated T cells, we compared PD-1 detection with EH12.2H7 and another non-competing anti-PD-1 antibody.^12^ Control cells were positive for both antibodies, but nivolumab-treated cells were only detected by the non-competing clone, indicating EH12.2H7’s competition with nivolumab. In line with *in vitro* data, the majority of PD-1^+^ T cells in CPI-treated biopsies could not be detected using both antibodies, while most PD-1^+^ cells were double-positive in healthy control samples (Figures 2F–2J), confirming that nivolumab binding, not PD-1 downregulation, affected PD-1 detection with EH12.2H7.

Phase 1 clinical trial data for nivolumab suggest that T cells remain drug-bound for at least 60 days, and likely up to 200 days.^13^ In our cohort, most samples were collected within 100 days of the last administration of CPIs. One sample with 400 days between last dose of immunotherapy and sample collection showed a reversion to unbound cells (Figure 2I). All patients within our CITE-seq dataset received their last dose of immunotherapy within 113 days (median 21 days, range 6–113 days), suggesting that indeed these cells were very likely to still be CPI-bound.

Next, we leveraged this information to identify individual, CPI-bound cells in our scRNA-seq data. Our strategy (Figure 2K) trained a quantile regression random forest^14^ model to predict PD-1 expression in non-checkpoint-treated samples using *PDCD1* mRNA expression values, QC-related metadata features, and integrated, reduced dimension components (Figure S4F). Predicting PD-1 expression in our hold-out, non-checkpoint-treated sample testing dataset showed good correlation with measured PD-1 expression (Figure S4G). Unsurprisingly, there was little correlation between predicted and measured PD-1 expression in CPI-colitis samples (Figure S4H). Thus, we hypothesized that cells where predicted and measured PD-1 expression values disagreed were likely enriched for CPI-bound cells. To quantify this, for each cell, we calculated the probability of where the measured PD-1 expression falls within the conditional distribution of model-predicted PD-1 values and then identified cells at a range of confidence cutoffs as putatively bound T cells (Figures 2L, 2M, and S4H).

Bound T cells across all confidence intervals were largely consistent, with most high-confidence calls falling into T follicular helper cells (Tfh), Th17, Tc17, cycling and CD103^−/+^ HAVCR2^+^ clusters, as well as a smaller subset of Tregs (Figures 2L and 2M). Effector populations showed putative, low confidence binding at low frequency, while as expected, other cell populations, such as naive cells, IELs (intra-epithelial lymphocytes), and TRM cluster cells were not CPI bound. We further confirmed this by fluorescence-activated cell sorting (FACS) analysis, where PD-1-expressing cells in CPI-colitis samples, such as 76% of cycling/KI67^+^, 64% CD4^+^ CXCR5^+^ (Tfh), 74% of CD8^+^ CCR6^+^ (Tc17), 81% of CD4^+^ CCR6^+^ (Th17s), and 87.5% PD-1^+^ CD103^+^ (PD-1^+^ exhausted TRMs), were CPI bound (Figure S4I).

### Distinguishing T cells responsible for initiation versus perpetuation of CPI-colitis

**Figure 3:**
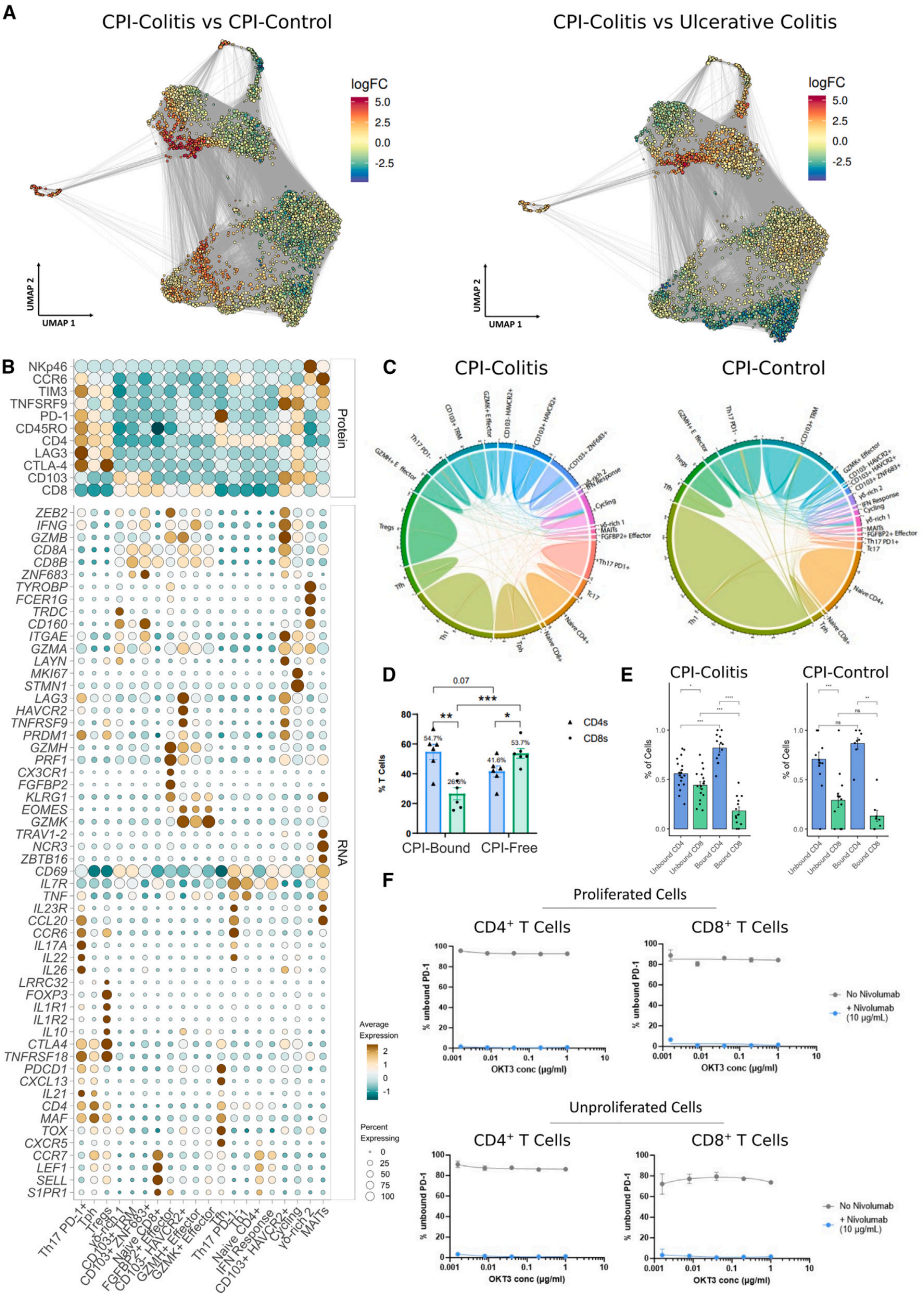
T cell landscape in CPI-colitis (A) UMAP overlay visualizing log-fold changes of local neighborhood abundance differences when comparing CPI-colitis and CPI-treated patient samples that did not go on to develop colitis (left) and CPI-colitis and ulcerative colitis patient samples (right). Each point represents a local neighborhood of cells within the single-cell k-nearest neighbor graph visualized as a UMAP embedding. Regions on the embedding with highest cellular enrichment in CPI-colitis samples are shown in red, while depleted regions are in blue. (B) Dot plot heatmap shows top T cell cluster marker transcript (bottom) and CITE-seq protein panel (top) distribution across all T cell clusters in tissue biopsy data. (C) Circos plot visualizes TCR clonal sharing and expansion levels between colonic tissue T cell populations in samples from CPI-colitis (left) and CPI control samples (right). (D) Bar plot visualizing the proportion of detected CPI-bound CD4^+^ and CD8^+^ T cells, respectively, compared with CPI-free ratio of CD4^+^-CD8^+^ T cells in colonic biopsy samples of CPI-colitis by FACS analysis. Unpaired t test with Welch’s correction, mean values are shown, error bars represent SEM. *p* value < 0.05 ∗; *p* value < 0.01 ^∗∗^*p* value < 0.001∗∗∗; ns = not significant. *n* = 6 samples. (E) Proportion of detected CPI-bound CD4^+^ and CD8^+^ T cells in CPI-colitis (left) and CPI-control (right) samples by CPI-binding prediction data from scRNA-seq analysis. Wilcox rank test, *n* = 8–18 per group, mean values are shown, error bars represent SEM. ^∗^*p* value < 0.05; ^∗∗^*p* value < 0.01; ^∗∗∗^*p* value < 0.001; ^∗∗∗∗^*p* value < 0.0001; n.s. not significant. (F) CPI binding retention rates of CD4^+^ and CD8^+^ T cells following 5-day *in vitro* stimulation culture. Proliferating cells (as measured by CFSE signal loss, top panels) and un-proliferating cells (bottom panels) visualized separately. Plots represent mean, error bars standard deviation. See also Figures S3–S6.

Next, we carried out differential abundance analysis to determine T cell states over- or underrepresented in CPI-colitis and whether these correlated with CPI-binding enrichment and/or trafficking (Figures 3A and S4J–S4M). In line with previous reports,^3^^,^^4^ within CD8^+^ T cells, we found an enrichment of two subsets of CD103^+^ TRM cells: a group characterized by expression of “exhaustion” markers (CD103^+^ HAVCR2^+^) and an activated ZNF683^+^ cell state (Figure 3B). The ZNF683^+^ cells were highly clonal, shared TCRs with actively proliferating T cells, but not cells from blood (Figures 3C, S4N, and S5A–S5D). Their phenotype was consistent with a shift away from long-term memory TRMs (IL7R^+^) toward a re-activated TRM phenotype (ZNF683, EOMES, PRDM1, granzymes) (Figures 3B and S6A–S6F). They expressed little *PDCD1* or *CTLA4* and displayed limited CPI engagement, suggesting that these TRM-derived cytotoxic CD8^+^ T cells were not activated due to direct effects of CPI binding.

Conversely, CD103^+^ HAVCR2^+^ cells showed high enrichment in CPI-bound cells (Figures 2L, 2M, and 3B), confirming their role in the pathogenesis of CPI-colitis as previously reported.^3^^,^^4^ Furthermore, as these cells showed strong CPI engagement, they must have existed in this state, including PD-1 expression, prior to development of colitis. Using TCR analysis, we further identified specific CPI-bound T cell clones (Table S3), which adopted complex cell fates, sharing clonality with other CPI-bound and CPI-free TRMs, Tc17s, IELs, and cycling T cells, but not T cells in circulation (Figures 3C, S4N, and S5A–S5G). This suggested this cluster was composed of “exhausted” resident cells with varied roles prior to inducing co-inhibitory gene expression; furthermore, not all clonal expansion could be explained by CPI binding, with many clones likely expanded prior to CPI administration.

In addition to resident T cells, we also observed increases in CD103^−^ cytotoxic effector T cell clusters (Figure 3A), characterized by expression of high levels of T cell activation and cytotoxicity molecules, and a further subset (CD103^-^ HAVCR2^+^) showing an analogous “exhaustion” gene expression profile observed in resident cells (Figures 3B and S6H). TCR analysis showed that these T cells did not share many clones with actively cycling T cell populations in tissue, instead showing a strong clonal overlap with T cells from blood, suggesting these cells expand in the periphery (Figures 3C, S4N, and S5B–S5D). In line with this, overall, we observed more TCR clonal sharing in CD8^+^ T cells in CPI-colitis when compared to non-inflamed control samples, but notably less than observed in UC (Figures S5B–S5D). Cells recruited from circulation also showed limited CPI engagement, despite upregulating *PDCD1* when compared with shared circulating clones and tissue controls (Figure S5E). Taken together, this suggests that although cytotoxic T cells are the major source of pro-inflammatory cytokines like IFNγ/TNF in CPI-colitis (Figure 3B), the recruitment of these specific subsets to the intestine from the periphery is likely a secondary event to CPI binding.

While these analyses delineate CPI-targeted and non-targeted but activated CD8^+^ states, it is striking that with the exception of HAVCR2^+^ and cycling cell clusters, other cytotoxic CD8^+^ T cells showed little CPI binding. In contrast to cancer tumor-infiltrating cells, the majority of CD8^+^ T cells in the colon, even under inflammatory conditions, do not express *PDCD1* (Figure S6F); thus, lower CPI engagement of CD8^+^ T cells is likely to arise due to differences in receptor availability in the colon. Furthermore, to maintain tolerance toward high load of commensal antigens at barrier sites, colonic CD8^+^ T cell activation is tightly controlled by additional mechanisms outside of PD-1/CTLA-4 pathways, including via innate-like inhibitory KLR/KIR receptors^15^ (Figure S6G).

Instead, we found that CPI target occupancy in CPI-colitis is overall greater within CD4^+^ T cells than CD8^+^ T cells (Figures 3D and 3E), in line with constitutive rather than induced PD-1 expression in specific colonic CD4^+^ populations. PD-1 is essential for both modulating Treg functions^16^^,^^17^ and effective B cell help by Tfh cells.^18^^,^^19^ Indeed, the loss of Tregs, which comprise a relatively small population in the gut, leads to gastrointestinal inflammation,^20^ suggesting that colitis initiating populations may not necessarily correspond to the most abundant/expanded T cell subtypes. We also identified a cluster of recently described T peripheral helper cells (reviewed in the study by Huang et al.^21^) in inflamed samples in both CPI-colitis and UC controls, which also expressed high levels of PD-1 and showed high levels of target engagement. These cells are thought to be capable of providing B cell help and likely drive the formation of tertiary lymphoid structures but are distinct from classical Tfh populations. In addition, a PD-1^+^ Th17 subset also showed high fraction of CPI binding and was also increased in abundance in CPI-colitis over controls (Figures 3A and S4J–S4M). In CPI-colitis, in addition to expressing transcriptional programs associated with type-17 T cells (*RORC*, *IL23R*, *IL17A*), PD-1^+^ Th17s were also a major source of IFNγ, while PD-1^−^ Th17s and Th17s in control samples had low IFNγ expression (Figure 3B).

We further examined the cytokine and chemokine profiles of all at least minimally expressed cytokines, chemokines, and TNF superfamily genes of CPI-bound and CPI-free CD4^+^ and CD8^+^ T cells and in line with cluster enrichment, observed that CPI-bound CD8^+^ T cells expressed not only more IFNγ, but also were more associated with type-17 cytokine profile than CPI-free CD8^+^ T cells. This was also true for CD4^+^ T cells (Figures S6I and S6J).

We next asked whether lower target engagement rates of CD8^+^ T cells when compared to CD4^+^ T cells were due to their increased proliferation potential, and whether initially CPI-bound CD8^+^ T cells had already diluted/recycled cell-surface CPI-bound PD-1 at the time of sample collection and CD4^+^ T cells retained CPI-binding for longer. We incubated T cells *in vitro* with or without nivolumab and stimulated them with plate-bound OKT3 to induce proliferation. Using CSFE to track cell divisions, we observed that after 5 days in culture both proliferated and non-proliferated CD4^+^ and CD8^+^ T cells retained CPI binding (Figures 3F and S6K). In our single-cell data, we found that there was no correlation between bound CD4/CD8^+^ T cell ratio with lag since last dose of immunotherapy. However, as most of the samples in our cohort received their last dose of immunotherapy within a month of sample collection, we further examined CPI-colitis samples with longer lag times (up to 304 days since last immunotherapy dose) by FACS analysis. While these data showed the expected decrease in overall binding, CD4/CD8 ratio remained constant (Figure S6L). Taken together, this suggests that nivolumab-PD-1 turnover is not substantially different between CD4^+^ and CD8^+^ T cells and CD4^+^ T cells are major CPI targets in colitis.

### CPI-bound and CPI-free T cells localize to divergent tissue regions in CPI-colitis

**Figure 4:**
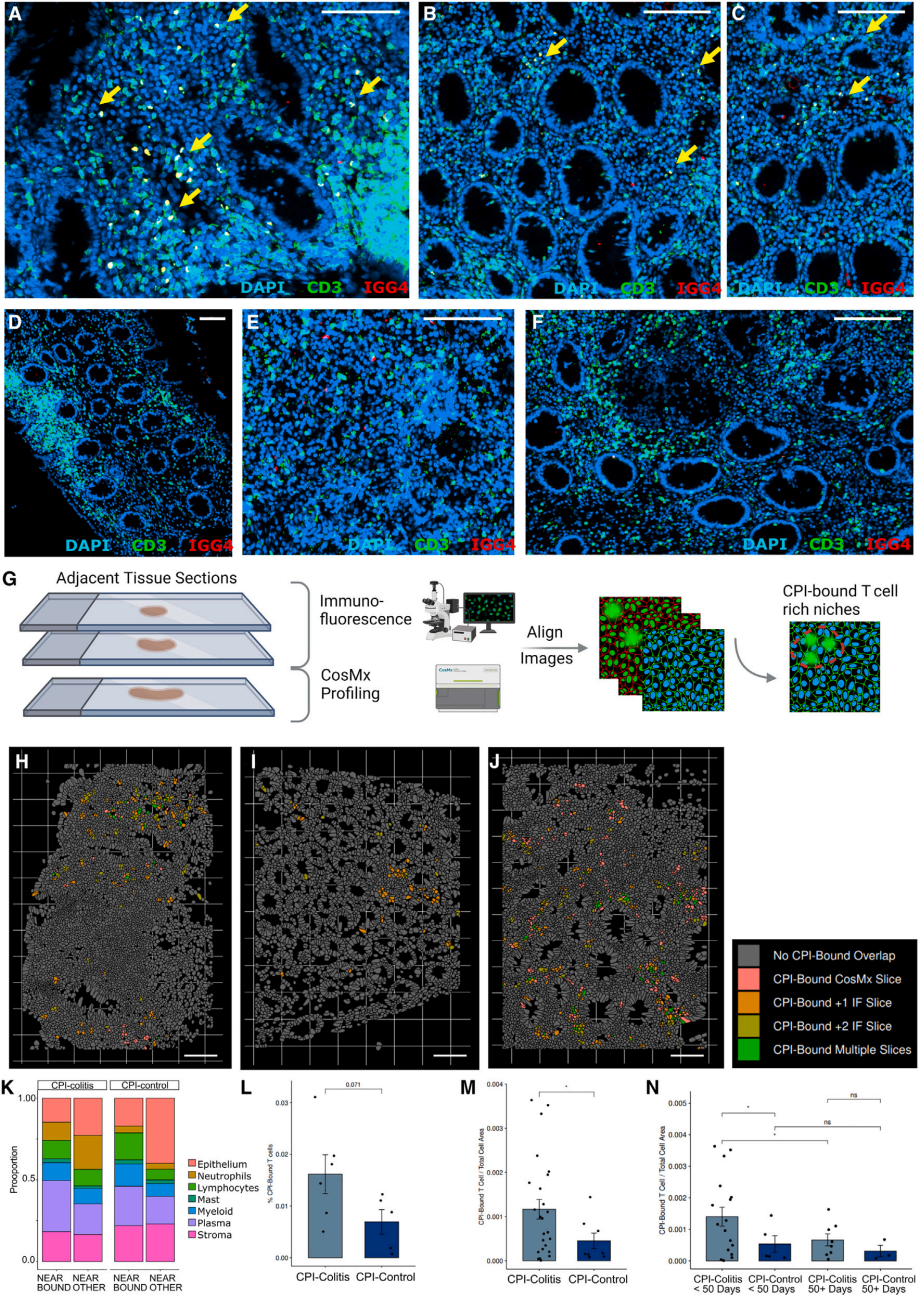
Identification of CPI-bound T cells *in situ* (A–F) Representative immunofluorescence (IF) images showing detection of CPI-bound T cells via combination of IgG4 (red) and CD3 (green). Yellow arrows indicate examples of double-positive IgG4^+^ CD3^+^ cells. Representative images from CPI-treated samples (A–C) and non-treated inflamed control samples with T cell-rich regions (D–F) are shown. Scale bar, 100 μm. (G) Schematic overview of the experimental strategy for detection of spatial regions in CosMx ST dataset enriched for CPI-bound T cells. (H–J) Selected fields of view in subcellular resolution CosMx ST dataset visualizing the spatial distribution of CPI-bound T cells detected using both tissue morphology markers (CosMx Slice, shown in salmon) and cells overlapping CPI-bound T cells detected by IF in two directly adjacent tissue slices (shown in green/orange). Examples of CPI-bound cells enriched in lamina propria regions (H), surrounding small lymphoid aggregate (I) and lamina propria and peri-follicular regions (J) are shown. Scale bar, 100 μm. (K) Proportion bar plot showing the distribution of broad cell populations overlapping CPI-bound T cells in adjacent IF slices in CPI-colitis and CPI-control samples. (L) Bar plot (mean, with +/− SEM) showing CPI-bound T cells detected in CosMx samples (adjacent IF sections), as a fraction of total cells in each section in CPI-colitis and CPI-control samples. Wilcox rank test, *n* = 5–6 per group. (M) Bar plot (mean, with +/− SEM) showing CPI-bound T cells detected in IF colonic tissue sections from a cohort of 33 donors of CPI-colitis (*n* = 25) and CPI-control (*n* = 8) samples. Wilcox rank test. ^∗^*p* value < 0.05. (N) As in (M), except samples are further grouped by CPI administration to sample collection lag. Bar plot shows mean, with +/− SEM. Wilcox rank test. ^∗^*p* value <0.05, ns - not significant. See also Figure S7.

To examine targeted T cell locations in colonic tissues, we distinguished between CPI-bound and CPI-free T cells using a combination of anti-IgG4 and anti-CD3 antibodies, leveraging the IgG4 hinge domain in nivolumab and pembrolizumab recognized by the HP6025 IgG4 antibody.^22^^,^^23^ We verified that we could detect CD3^+^IgG4^+^ cells by immunofluorescence (IF) in tissue sections from CPI-treated patients, but not from untreated samples (Figures 4A–4F).

For subcellular resolution spatial analysis using CosMx platform, we included both CD3 and IgG4 antibodies as part of the morphology staining panel and in line with IF, could detect double-positive CD3^+^ IgG4 cells in sections from CPI-treated patients, but not controls (Figure S7A). However, CD3^+^ cells were rare, and many T cells identified by transcriptome analysis were missed, likely due to epitope cleavage during harsh protease treatment required during the tissue processing step. Thus, to further explore tissue niches harboring CPI-bound T cells, we undertook IF of two immediately adjacent tissue sections and aligned the images to our CosMx ST data, with IF section morphology showing very close correspondence to our ST sections (Figure 4G). We found that CPI-bound T cells identified by both IF slices and CosMx morphology staining corresponded to the equivalent regions (Figures 4H–4J and S7B), further validating our approach.

This analysis revealed that several CPI-bound T cells often appear clustered together within certain lamina propria regions, often near other unbound T cells, but not between epithelial cells (Figures 4A–4F, 4H–4K, and S7B), in line with the observation that IELs in our single-cell data were almost never CPI bound. Conversely, often lamina propria regions even a few crypts away were occupied by unbound T cells only (Figures 4A–4C). Some CPI-bound T cells localized within lymphoid follicles, within the T cell zone near the outer edges of the structure, in line with our scRNA-seq data suggesting that a high proportion of Tfh and Tph cells are CPI bound (Figures 4I and 4J). On the other hand, in other sections, we observed several follicles entirely devoid of CPI-bound T cells (Figure 4H), suggesting these structures may be newer and formed post-CPI treatment.

We next quantified CPI-bound T cells detected in CPI-colitis and CPI-control samples and found that the number of CPI-bound T cells (normalized to tissue area) was notably increased in CPI-colitis sections when compared to controls (Figure 4L). We confirmed this trend by IF from additional 33 patient samples. Furthermore, we also observed a decrease in CPI-bound T cells in patients where CPI treatment was administered more than 50 days prior to sample collection when compared to patients where the time lag was less than 50 days (Figures 4M and 4N). Taken together, this suggests that (a) CPI-bound cells are present in colonic tissue of CPI-treated patients who did not go on to develop colitis, suggesting that target engagement of colonic T cells alone is not sufficient to induce colitis, and (b) CPI target availability and/or engagement at the time of drug administration may be higher in patients that go on to develop colitis vs. those that do not, and may be clinically predictive.

### Chemotactic, pro-inflammatory tissue niches are enriched in highly proliferative but CPI-free CD8^+^ T cells in CPI-colitis

**Figure 5:**
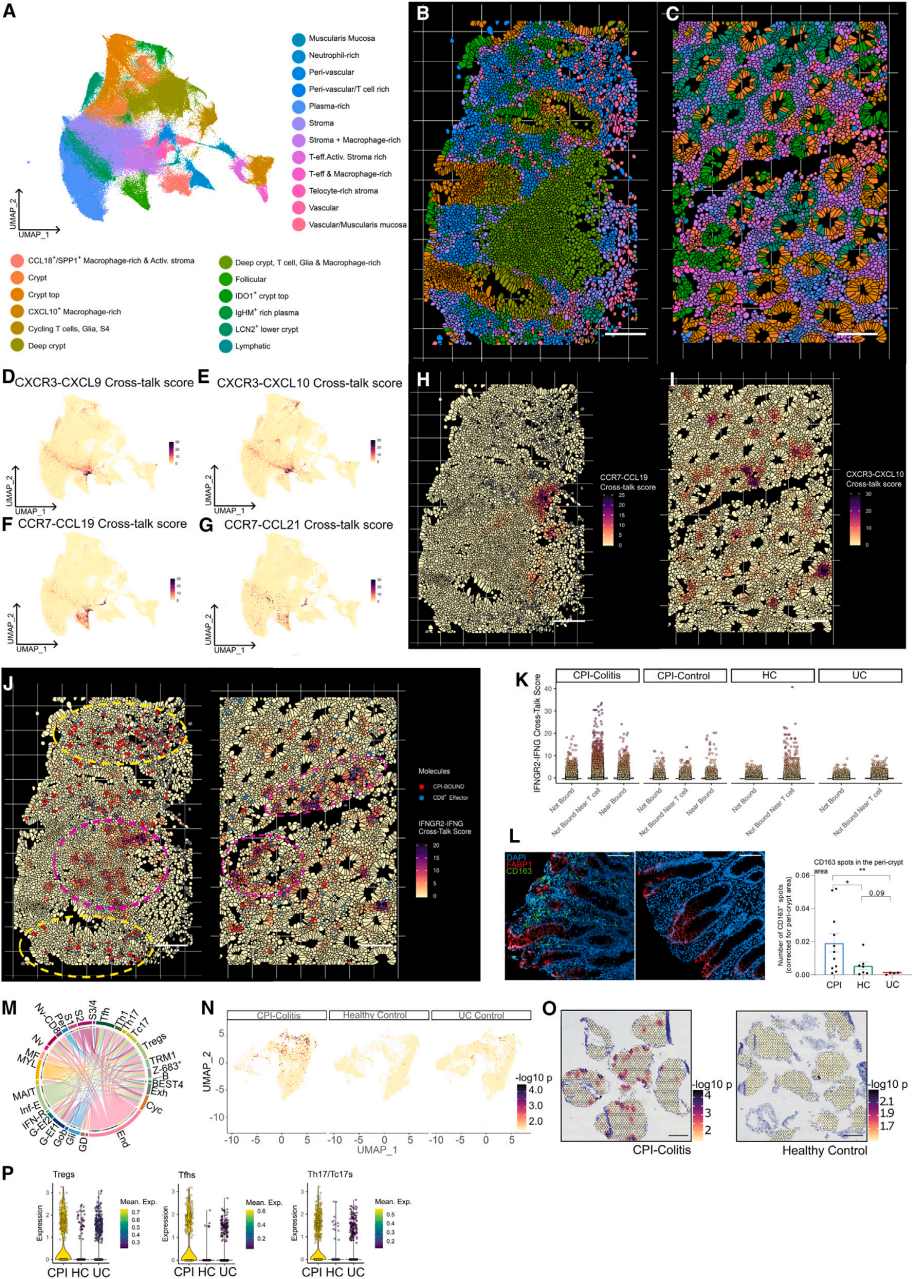
Tissue microdomains in CPI-colitis (A) UMAP embedding visualizing spatial niches, clustered using local cell neighborhood composition of individual single cells. Niches are labeled based on anatomical tissue structures they correspond to, or cell type enrichments. (B and C) Spatial niche overlay in selected fields of view in CPI-colitis. Scale bar, 100 μm. (D–G) Spatial niche UMAP overlay of local neighborhood cellular cross-talk scores, showing differential local enrichment of T cell chemotactic cellular signaling via CXCR3-CXCL9 (G), CXCR3-CXCL10 (E), CCR7-CCL19 (F), and CCR7-CCL21 (G) in different spatial niches. (H and I) Selected field of view overlay of local cellular cross-talk score distribution in CPI-colitis sections showing enrichment of CCR7-CCL19 (H) near lymphoid structures and CXCR3-CXCL10 (I) signaling in lamina propria regions, often regions with evidence of tissue damage. Scale bar, 100 μm. (J) Selected field of view from CPI-colitis sample ST dataset visualizing local IFNγ signaling score distribution in relation to regions enriched for CPI-bound T cells (red) or CD8^+^ effector T cells (blue). Regions rich in CPI-bound cells are highlighted in yellow, regions rich in CD8^+^ effector T cells are highlighted in purple. Scale bar, 100 μm. (K) Interferon gamma local signaling score distribution in cells nearby CPI-bound T cells, nearby other T cells, or nearby other cells in CPI-colitis and control samples. (L) Representative IF images in CPI-colitis (left) and UC (middle) in colonic tissue sections showing the distribution of CD163 expressing cells (green) in relation to epithelial crypts. FABP1 (red) is used as a crypt top/gradient marker. Scale bar, 100 μm. Bar plot (right) showing the number of CD163^+^ peri-cryptal spots, corrected for total peri-cryptal area, in CPI-colitis, UC, and HC tissue sections. Unpaired t test with Welch’s correction, mean values are shown, error bars represent SEM. *p* value < 0.05 ∗; *p* value < 0.01∗∗; ns = not significant. *n* = 4–11 samples per condition. (M) Receptor ligand analysis of spatial and cell type distribution of CXCL11-CXCR3 signaling. Circos plots showing source (ligand) and target (receptor) expressing cell populations in CPI-colitis samples. Coord width is scaled to expression level. scRNA-seq populations are abbreviated as follows: Gli, glial cells; GD,gamma-delta cells; F-Ef., FGFBP2^+^ effector T cells; Ent, enterocytes; End, endothelial; Cyc, cycling cells; Exh – CD8^+^ HAVCR2^+^ T cells; B, B cells; Z-683^+^ - CD103^+^ ZNF683^+^; S3/S4, stromal 3 & 4; S2, stromal 2; S1, stromal 1; Gob, goblets; Per, pericytes; Nv CD8, naive CD8^+^ T cells; Nv, naive CD4^+^ T cells; MF, myofibroblasts; MYL, myeloid cells; IFN-R, interferon response B cells; G-Ef1, GZMK^+^ effector T cells; G-Ef2, GZMH^+^ effector T cells; CT-E, crypt top enterocytes; TAs, transit amplifying epithelium; PL, plasma cells; EEC, enteroendocrine cells; Abs and Abs2, absorptive epithelial cells 1 and 2. (N) UMAP overlay of spatial distribution of significant co-localization of CXCL11-CXCR3 spots in CPI-colitis, healthy and UC control slides. (O) Representative ST slides showing CXCL11-CXCR3 spots with significant co-localization in CPI-colitis (left), and healthy control slides (right). Scale bar, 1 mm. (P) Violin plots show upregulation of *CXCR3* in CPI-colitis in Tregs (left, CPI-colitis vs. HC – *p* = 9.875531e-06, CPI-colitis vs. UC - 3.891223e-15, negative binomial test), Tfhs (middle, CPI-colitis vs. HC – *p* = 0.0008017907, CPI-colitis vs. UC - 5.791019e-08, negative binomial test), and Th17/Tc17s (right, CPI-colitis vs. HC – *p* = 0.4290415, CPI-colitis vs. UC – *p* = 1.081205e-06, negative binomial test). Center bar indicates median value; color indicates mean expression. See also Figures S7–S9 and Table S3.

In order to better understand tissue damage pathways in CPI-colitis and identify spatial microdomains enriched for CPI target and CPI-free T cells, we partitioned our high-resolution single-cell data into spatial niches defined by their cellular composition. This approach was able to group cells locally in each FOV (field of view) into common structural domains, recapitulating known tissue morphology—e.g., deep crypt and crypt top niches, lymphoid follicles, muscularis mucosa, lymphatics, and vascular/peri-vascular regions—as well as identifying multiple lamina propria cellular domains that otherwise could not be identified using histopathology alone (Figures 5A–5C).

We identified several tissue niches that were specific to CPI-colitis sections (Figure S7C). CPI-specific niches could be differentiated by the composition of different immune sub-populations (Figure S7D) and were analogous to spatial clusters identified in our Visium ST sections (Figures 1C and 1D). Spatial cell-cell signaling analysis revealed that this diversity in immune cell localization could be largely explained by niche-specific chemotactic signaling gradients (Figures 5D–5I and S7E). Regions with significant neutrophil infiltration showed a high degree of CXCR2-CXCL8-mediated chemotaxis. T cells were primarily enriched in niches with high chemotactic activity either via CXCL9/CXCL10-CXCR3 or CCR7-CCL19/CCL21 axes, corresponding respectively to regions within the lamina propria or discrete lymphoid aggregates (Figures 5D–5I). Cytotoxic CD8^+^ effector T cells, TRMs, and cycling CD8^+^ T cells were found to mostly localize in the lamina propria near epithelial crypts, often congregating in small microdomains (Figures S7F and S7G). Tissue composition and scRNA-seq analyses highlighted the involvement of epithelial cells, activated fibroblasts, neutrophils, and glial and endothelial cells in orchestrating these chemotactic domains (Figures 5D–5I, S7D, and S8A). IFNγ signaling was also locally enriched in these spatial regions (Figures 5J and S8B). *CXCL9/10* induction occurs through interferon signaling,^24^^,^^25^ indicating a feedforward loop of CD8^+^ activation and migration is perpetuated within the local tissue microenvironment in CPI-colitis.

Nonetheless, most of the T cells in these highly pro-inflammatory, CD8^+^ T effector rich niches remained CPI-free (Figure 5J). We compared cellular signaling patterns near CPI-bound and CPI-free T cells and found that both chemotactic signaling via CXCR3 and IFNγ signaling were more strongly enriched near CPI-free T cells without CPI-bound T cells in the local vicinity (Figure 5K), indicating that activated CPI-free cells directly contribute to the local pro-inflammatory cytokine milieu.

### Upregulation of diverse immune suppression pathways is insufficient to dampen local immune response in pro-inflammatory crypt niches in CPI-colitis

In CPI-colitis epithelia, we found two unique cell types: IDO1^+^ cells at crypt tops facing the lumen and LCN2^+^ cells in deeper crypts, both showing increased interferon response and epithelial regeneration pathways (Figures 5A–5C and S8C; Table S3). These cells segregated to their own distinct epithelial niches, typically within entire, specific crypts within the tissue and were often adjacent to cytotoxic T cell-rich lamina propria domains, confirming that these changes within the epithelia are in response to localized pro-inflammatory signaling (Figures 5A–5C).

IDO1^+^ cells (Figure S8C–S8E), which may promote Treg polarization via kynurenine pathway,^26^^,^^27^ were also often adjacent to a layer of macrophages directly lining crypt top cells. We mapped these macrophages to a CPI-colitis-specific, CD163^+^ M2-like cluster (Figures 5L, S8F, and S8G; Tables S2 and S3) that specifically expressed *CCL18*, which may further promote Treg recruitment and differentiation.^28^^,^^29^ This was supported by our single-cell data (Figures S8F, S8G, and S9A; Tables S2 and S3), where in CPI-colitis there was an enrichment of cells expressing *CCL18* and *CD163*^30^^,^^31^^,^^32^ and TF networks associated with increased lipid metabolism (Figures S9A and S9B), which can inhibit expression of pro-inflammatory genes.^33^^,^^34^^,^^35^ CCL18 itself may act as an autocrine M2 differentiation factor,^29^ perpetuating this local macrophage phenotype. We localized these signatures to the myeloid-rich crypt-top regions in Visium ST CPI-colitis slides (Figure S9B) which were verified by IF (Figures 5L and S9C).

Epithelial cells from these involved crypts were also one source of *CXCL11* (Figures 5M–5O and S8A; Tables S2 and S3). While both CXCL9/10 and CXCL11 are T cell chemoattractants that can be induced by interferon signaling in epithelial cells, which we verified in epithelial organoid cultures (Figure S9D), *CXCL11* specifically promotes both Treg polarization and recruitment, while CXCL9/10 polarize T cells toward pro-inflammatory effector phenotypes.^36^ We observed a strong induction of *CXCR3* expression in CPI-bound Tregs (as well as Tfh and PD-1^+^Th17 cells) in CPI-colitis (Figure 5P). CXCR3 upregulation has been shown to be induced by both direct PD-1 inhibition^36^^,^^37^ and interferon gamma signaling.^38^ In line with this, unbiased ST analysis of inflamed, IDO1^+^ crypt top spots identified specific increase in Treg signature gene expression in CPI-colitis but not UC (Figure S9E).

Taken together, this suggests that in CPI-colitis, individual crypts and nearby cells are actively working to suppress CD8-mediated inflammation by enhancing Treg polarization/recruitment. However, CPI binding to in Tregs may render their suppressive activity inadequate.^39^

### Inflammation in CPI-colitis and UC diverges in lymphoid follicle-associated responses

UC is a complex inflammatory disorder involving colonic barrier dysfunction, genetic predisposition, and immune dysregulation.^40^ Despite advances in understanding the cellular pathology of UC, failure to respond to current therapies occurs in up to 40% of patients. CPI-colitis is empirically treated utilizing the same drugs as UC,^41^^,^^42^^,^^43^ an approach beset with similar failure rates. However, we do not know whether colitis arising from different etiologies leads to identical or disparate cellular pathology. Elucidating these mechanisms of tissue destruction occurring in either disease is required to develop precision medicine approaches. This knowledge is essential for the treatment of CPI-colitis, where it is desirable to inhibit off-target immune activation but preserve anti-tumor responses.

**Figure 6:**
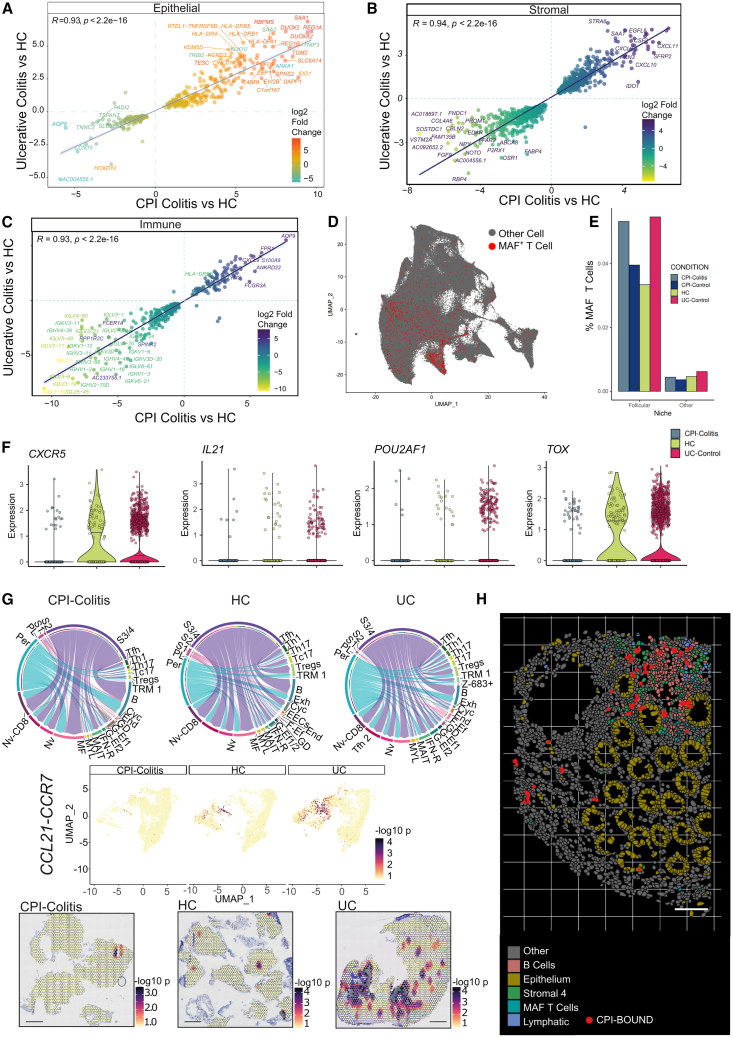
Differential tissue responses between UC and CPI-colitis (A–C) Correlation plot visualizing all significantly differentially expressed genes detected when comparing CPI-colitis vs. healthy and UC vs. healthy in epithelial (A), stromal (B), and immune (C) cells. (D) UMAP overlay of spatial niche embedding visualizing the niche distribution of *MAF*^+^ T cells, enriched in but not exclusively within follicular tissue niches. (E) Bar plot showing the percentage of MAF^+^ T cells within follicular and other spatial niche types in CPI-colitis and control samples. (F) Violin plots showing downregulation of expression of *CXCR5* (CPI-colitis vs. UC, *p* = 9.77127e-17, CPI-colitis vs. HC, *p* = 3.66914e-09, negative binomial test), *IL21* (CPI-colitis vs. UC, *p* = 0.8696069, CPI-colitis vs. HC, *p* = 0.01183161, negative binomial test), *POU2AF1* (CPI-colitis vs. UC, *p* = 0.0006161389, CPI-colitis vs. HC, *p* = 0.0009668945, negative binomial test), and *TOX* (CPI-colitis vs. UC, *p* = 0.001535492, CPI-colitis vs. HC, *p* = 0.1449066, negative binomial test) in Tfh cells in CPI-colitis. Center bar indicates median value; color indicates mean expression. (G) Receptor ligand analysis of spatial and cell type distribution of CCR7-CCL21 signaling. Top: Circos plots showing source (ligand) and target (receptor) expressing cell populations in CPI-colitis (left), HC (middle), and UC (right) samples. Coord width is scaled to expression level. Middle: UMAP overlay of spatial distribution of significant co-localization of CCR7-CCL21 spots in CPI-colitis, HC, and UC slides. Bottom: Representative ST slides showing CCR7-CCL21 spots with significant co-localization in CPI-colitis (left), HC (middle), and UC (right). Scale bar, 1 mm. scRNA-seq populations are abbreviated as follows: Gli, glial cells; GD, gamma-delta cells; F-Ef., FGFBP2^+^ effector T cells; Ent, enterocytes; End, endothelial; Cyc, cycling cells; Exh, CD8^+^ HAVCR2^+^ T cells; B, B cells; Z-683^+^, CD103^+^ ZNF683^+^; S3/S4, stromal 3 & 4; S2, stromal 2; S1, stromal 1; Gob, goblets; Per, pericytes; Nv CD8, naive CD8^+^ T cells; Nv, naive CD4^+^ T cells; MF, myofibroblasts; MYL, myeloid cells; IFN-R, interferon response B cells; G-Ef1, GZMK^+^ effector T cells; G-Ef2, GZMH^+^ effector T cells; CT-E, crypt top enterocytes; TAs, transit amplifying epithelium; PL, plasma cells; EEC, enteroendocrine cells; Abs and Abs2, absorptive epithelial cells 1 and 2. (H) Selected field of view containing a lymphoid follicle in CPI-treated colonic tissue sample highlighting selected follicular cell types detected by clustering analysis (B cells, stromal 4/lymphoid organizer cells, lymphatic cells, and *MAF*^+^ T cells), as well as landmark epithelial cells. Cells overlapping CPI-bound regions in adjacent IF tissue sections are indicated in red, enriching around the lymphoid follicle. Scale bar, 100 μm. See also Tables S2 and S3.

In our data, principal component analysis of pseudo-bulk epithelial, stromal, and immune cells showed clear separation between inflamed and non-inflamed conditions, while differences between CPI-colitis and UC-inflamed samples were less pronounced (Figures S1A–S1D). Differential gene expression and cell abundance analyses highlighted that in stromal and epithelial cells, CPI-colitis and UC inflammation converged along similar transcriptomic and cellular responses (Figures 6A–6C). These responses were enriched for highly similar pathways and functions (Table S3), including response to interferon gamma, and antigen-processing and presentation pathways.

Notably, the differences between inflamed UC and CPI-colitis samples were greater when looking within immune cell populations, including T cells (Figure S1D). In UC, in contrast to CPI-colitis, we observed depletion in TRMs (Figures 3A and S4J–S4M). In line with this, there was an increase in tissue egress and lymph node homing marker expression in cytotoxic CD8^+^ T cells in UC (Figure S10A), as well as an increase in naive T cells in the gut (Figures 3A and S4J–S4M) and increased clonal sharing between paired blood and tissue samples (Figures S5B–S5D).

Within the CD4^+^ compartment, our scRNA-seq dataset identified four clusters of PD-1^+^ T cells—Tregs, a canonical CXCR5^+^ Tfh population, a CXCR5-low Tfh cluster, and a unique PD-1^+^ Th17 population (Figures 2A and 3B; Tables S2 and S3). The latter two populations likely represent two distinct T peripheral helper cell (Tph) subtypes. These cells share Tfh-associated TF MAF regulon activity, express ICOS, TIGIT, and PD-1/*PDCD1*, and produce some IL-21 but retain limited expression of CXCR5 and CXCL13 (Figure 3B). Th17 PD-1^+^ cells upregulated PRDM1/Blimp1, which in opposition to Tfh-specific BCL6 has been shown to be specifically upregulated by Tph cells in other inflammatory contexts.^44^^,^^45^ Both CXCR5-low Tph cells and PD-1^+^ Th17s were expanded in CPI-colitis while cells in UC were shifted toward a classical Tfh phenotype. Expanded Tph cells may represent an early stage of follicle formation,^46^ or have different migration/localization patterns in tissue and provide B cell help outside of mature tissue lymphoid structures.^46^ Indeed, while the equivalent PD-1^+^ MAF^+^ T cells identified in our high-resolution ST dataset was the most enriched T cell subpopulation in follicular regions, a large proportion of these cells nonetheless localized away from lymphoid structures (Figures 6D and 6E). Co-localization analysis highlighted that even outside of follicular regions, there was a greater than expected B cell to MAF^+^ T cell adjacency (Figure S10B), consistent with extra-follicular B cell help roles of these cells.

Differential expression and signaling analysis of scRNA-seq Tfh populations highlighted that several genes critical to Tfh functions were downregulated in CPI-colitis but not UC, including *POU2AF1* (required for germinal center formation),^47^ *CXCR5* (germinal center localization), and its ligand *CXCL13* and *IL21* (cytokine required for germinal center formation)^48^(Figure 6F; Tables S2 and S3). In ST data, we identified a significant reduction in the activity in key chemotactic signaling pathways in CPI-colitis compared to UC (Figures S10C and S10D), including CXCR5-CXCL13 and CCR7-CCL19/CCL21 axes that induce both T cell and T cell recruitment.^49^^,^^50^ CCL21/CCL19 is co-expressed solely by “S4” type fibroblasts, which have been previously reported to be a hallmark of UC^7^^,^^51^ and which we localized to regions within and surrounding lymphoid structures. In lymphoid structures in CPI-colitis, we observed that S4 cell/expression of CCL19/CCL21 was contained within these structures; however, in UC, these cells were more widespread in the surrounding stroma.

While mature follicular structures were overall rare in tissue sections, they were more prevalent in UC than CPI-colitis in ST data and an independent cohort of histological tissue sections, an effect that could not be explained by overall disease chronicity/duration of inflammation (Figures 6H, S10E, and S10F). Similarly, we did not observe a difference in Tfh abundance when comparing CPI-colitis patients in acute (<10 days of symptoms) and chronic inflammation (more than 1 month symptom duration) (Figures S10G and S10H).

Finally, we were able to detect CPI-bound T cells within and surrounding follicles in CPI-treated patients, confirming that Tfh cells are also *bona fide* targets of PD-1 inhibitors (Figure 6H). In mice, PD-1 has been shown to be required for maintaining the stringency of B cell selection and has an effect on Tfh migration and positioning within lymphoid follicles by attenuating TCR signaling in Tfh cells.^19^

## Discussion

In this work, we harnessed unbiased single-cell and subcellular ST to identify the cell states, intercellular signaling, and transcriptional gradients linked to tissue damage in CPI-colitis. Tracking CPI-bound T cells in multimodal single-cell and ST data enabled us to distinguish the effects of target occupancy on subsequent pathobiology, differentiating causal and perpetuating axes of inflammation.

Previously, it was thought that CPI-colitis might arise via reversal of the exhaustion phenotype of CD8^+^ T cells as a direct effect of CPI binding, analogous to their presumed effect in driving anti-tumor responses.^2^ We tracked nivolumab/pembrolizumab-bound T cells using both direct *in situ* tissue staining and *in silico* approaches and identified more CPI-bound CD4^+^ T cells than CD8^+^ T cells overall, suggesting that perturbed regulatory cues from targeted Tregs, Tfhs, Tphs, and Th17s may play a bigger role in colitis initiation than previously thought. For instance, it has been reported that PD-1 blockade on Tregs reduces their immune-suppressive functions;^39^ conversely, in mouse models, Treg-specific PD-1 deficiency enhances suppressive phenotype.^52^^,^^53^ Thus, while the role of PD-1 in Tregs remains to be concluded, Tregs express both PD-1 and CTLA-4, and in dual-therapy patients in particular, the exact effects of individual signaling pathway inhibition are difficult to tease apart. Unlike the kinetics of anti-PD-1 therapies, upon ipilimumab binding, CTLA-4 is internalized and rapidly recycled^54^^,^^55^^,^^56^ and therefore it is not possible to detect originally targeted T cells by the time of CPI-colitis onset to study these mechanisms in the same way.

Similarly, a large proportion of target-occupied CD4^+^ T cells identified are involved in follicular responses and B cell help. As a result, improperly coordinated B cell responses may fail to provide adequate barrier protection contributing to dysbiosis and may explain why fecal matter transplants show some efficacy in the treatment of CPI-colitis.^57^

CD8^+^ T cells implicated in tissue damage are thought to arise from TRMs, as these cells are highly clonally expanded and proliferative in CPI-colitis in our data and other studies.^3^^,^^4^ Here, leveraging our matched peripheral blood and mucosal sample TCR dataset, we nonetheless observed increased CD8^+^ trafficking, suggesting there is a secondary, re-circulating CD8^+^ response in CPI-colitis. In line with this, the gut-selective anti-a4b7 integrin monoclonal antibody vedolizumab has been successfully used to treat irAEs,^58^ which our data highlight would assist in depleting trafficking of peripherally expanded CD8^+^ T cells to sites of tissue damage in CPI-colitis.

Interestingly, in clinically inflamed tissue, crypt inflammatory microdomains were observed in a sporadic distribution with complete sparing of many crypts. It is possible this is reflective of susceptibility of some crypts to CD8^+^ T cell recognition, perhaps as a result of presentation of distinct microbial or self-antigens, pre-existing age-related somatic mutational landscape of individual crypts, or age-related epigenetic remodeling driving abnormal expression of endogenous retroviruses.

Increasing use of CPIs is driving unmet need in definition of markers predicting irAE occurrence and in tissue-specific understanding of pathobiology to enable design of niche-specific modulators. Here, we found that overall target engagement was increased in CPI-treated patients that developed colitis and may be an early predictor of patients at risk of developing irAEs. Furthermore, selective expression of IL-23R on the majority of PD-1-bound T cells in CPI-colitis suggests targeting the IL-23 axis might limit tissue-restricted side effects and emerging case reports of ustekinumab use are supporting this.^59^

### Limitations of the study

This study is a deep multi-modal single-cell and ST characterization of a CPI-colitis patient cohort. While we were able to identify and characterize individual CPI-bound T cells in our cohort, it is unclear how these populations evolve from homeostatic gut T cells and whether any steady-state differences of targeted populations lead to susceptibility to colitis in some patients—longitudinal sampling of pre- and post-treatment patients would be required to investigate this.

In the scRNA-seq cohort, CPI-bound cells were identified using a computational model and validated by flow cytometry. In future studies, anti-IgG4 and anti-PD-1 CITE-seq antibodies could be used to directly identify CPI-bound cells. Similarly, the ST platform methodology did not allow for the detection of CPI-bound T cells in the same tissue slices, so we used directly adjacent tissue, and in the future, it may become easier to undertake a simultaneous approach.

We identified CPI binding of follicular T cell populations; however, how this affects lymphoid follicle biology in the gut requires functional exploration. Finally, our approach cannot be used to detect anti-CTLA-4 CPIs due to rapid recycling of CTLA-4 from the cell surface. At the transcriptome level, we and other studies have detected few differences between monotherapy and dual-therapy patients that could not be accounted by other clinical variables (e.g., severity of inflammation); however, how CPI binding impacts and interacts with cells expressing both PD-1 and CTLA-4 (e.g., Tfh, Tregs) remains to be explored.

## Consortia

Dr Elizabeth Bird-Lieberman, Oxford University Hospitals NHS Foundation Trust.

Dr Simona Fourie, Oxford University Hospitals NHS Foundation Trust. Simona.fourie@rdm.ox.ac.uk

Dr Richard Johnston, Torbay and South Devon NHS Foundation Trust. richardjohnston@nhs.net.

Mr Heman Joshi, Cambridge University Hospitals NHS Foundation Trust. heman.joshi4@nhs.net

Dr Debabrata Mujamdar, Ashford and St Peter’s Hospitals NHS Foundation Trust. debarata.mujamdar@nhs.net

Dr Simon Panter, South Tyneside and Sunderland NHS Foundation Trust. simon.panter@nhs.net

Dr Nishant Patodi, Royal Berkshire NHS Foundation Trust. nishant.patodi@royalberksihre.nhs.uk

Prof Sebastian Shaji, Hull University Teaching Hospitals NHS Trust. shaji.sebastian@hey.nhs.uk

Dr Jude Tidbury, East Sussex Healthcare NHS Trust. judith.tidbury@nhs.net

Dr Ajay Verma, Kettering General Hospital NHS Foundation Trust. ajay.verma@nhs.net

## STAR★Methods

### Key resources table

**Table:** 

REAGENT or RESOURCE	SOURCE	IDENTIFIER
**Antibodies**		

E-cadherin rabbit anti-human antibody	Cell Signalling	Cat#3195, RRID: AB_2291471
CD103 mouse anti-human antibody	Abcam	Cat#ab238010 [ITGAE/2063]
DAPI (4′,6-diamidino-2-phenylindole) Solution	BD Pharminogen	Cat#564907
FITC-Conjugated anti-EPCAM	Miltenyi	Cat#130080301, RRID: AB_244192
APC-conjugated anti-CD45	Miltenyi	Cat#130113676, RRID: AB_2726217
Human Trustain FcX (Fc Block)	Biolegend	Cat# 422302, RRID: AB_2818986
PEDazzle-conjugated anti-CD3	Biolegend	Cat# 300449, RRID: AB_2563617
AF488 Goat Anti-Rabbit Secondary Antibody (Cross Adsorbed)	Thermo Fisher Scientific	Cat# A32731, RRID: AB_2633280
AF647 Goat Anti-Mouse Secondary Antibody (Cross Adsorbed)	Thermo Fisher Scientific	Cat# A-21235, RRID: AB_2535804
Totalseq C0251 Anti-Human Hashtag 1	Biolegend	Cat#394661, RRID: AB_2801031
Totalseq C-0252 Anti-human Hashtag 2	Biolegend	Cat#394663, RRID: AB_2801032
Totalseq C-0253 Anti-human Hashtag 3	Biolegend	Cat#394665, RRID: AB_2801033
Totalseq C-0254 Anti-human Hashtag 4	Biolegend	Cat#394667, RRID: AB_2801034
Totalseq C-0255 Anti-human Hashtag 5	Biolegend	Cat#394669,RRID: AB_2801035
Totalseq C-0046 Anti-human CD8	Biolegend	Cat#344753, RRID: AB_2800922
Totalseq C-0045 Anti-human CD4	Biolegend	Cat#344651, RRID: AB_2800921
Totalseq C-0087 Anti-human CD45RO	Biolegend	Cat#304259, RRID: AB_2800766
Totalseq C-0088 Anti-human PD-1 (Clone EH12.2H7)	Biolegend	Cat#329963, RRID: AB_2800862
Totalseq C-0101 Anti-human CD335 (NKp46)	Biolegend	Cat#331941, RRID: AB_2800874
Totalseq C-0143 Anti-human CD196 (CCR6)	Biolegend	Cat#353440, RRID: AB_2810563
Totalseq C-0152 Anti-human CD223 (LAG-3)	Biolegend	Cat#369335, RRID: AB_2814327
Totalseq C-0169 Anti-human CD366 (Tim-3)	Biolegend	Cat#345049, RRID: AB_2800925
Totalseq C-0355 Anti-human CD137 (4-1BB)	Biolegend	Cat#309839, RRID: AB_2800807
Totalseq C-0151 Anti-human CD152 (CTLA-4)	Biolegend	Car#369621, RRID: AB_2801015
Totalseq C-0145 Anti-human CD103	Biolegend	Cat#350233, RRID: AB_2800933
Totalseq C-0053 Anti-human CD11c	Biolegend	Cat#371521, RRID: AB_2801018
Totalseq C-0161 Anti-human CD11b	Biolegend	Cat#301359, RRID: AB_2800732
BV421-conjugated anti-KI67	Biolegend	Cat#350505, RRID: AB_10896915
Zombie Aqua fixable viability kit	Biolegend	Cat# 423101
FITC-conjugated anti-PD-1 (Clone EH12.2H7)	Biolegend	Cat #329904, RRID: AB_940479
BV605-conjugated anti-CD3	Biolegend	Cat #300459,RRID: AB_2564379
BV785-conjugated anti-CD8	Biolegend	Cat #344739, RRID: AB_2566201
FITC-conjugated anti-human mouse IGG1 Isotype control	Thermofisher	Cat#11-4714-42; RRID: AB_10596964
PE-conjugated anti-CCR6	Biolegend	Cat#353409, RRID: AB_10915968
PE-Cy7-conjugated anti-CD103	Biolegend	Cat#350211, RRID: AB_2561598
APC-Cy7- conjugated anti-CXCR5	Biolegend	Cat#356925, RRID: AB_2562592
AF647-conjugated mouse IGG1 isotype control	Biolegend	Cat#400135, RRID: AB_2832978
Non-competitive PD1 binding antibody (Clone 19)	Davis Group, WIMM	Not commercially available, validated in-house, see references.
CD3 rabbit anti-human antibody	Cell Signaling Technology	Cat# 85061, RRID: AB_2721019)
IGG4 mouse anti-human antibody	Bio-Rad	Cat# MCA2098G, RRID: AB_323685
iNOS2 mouse anti-human antibody	R&D Systems	Cat# MAB9502, RRID: AB_2152874
FABP1 rabbit anti-human antibody	Sigma-Aldrich	Cat# HPA028275, RRID: AB_10600909
Neutrophil elastase/ELA2 mouse anti-human antibody	Novus Biologicals	Cat# MAB9167
CD163 mouse anti-human antibody	Novus Biologicals	Cat# NB110-40686, RRID: AB_714951
FOXP3 rabbit anti-human antibody	Atlas Antibodies	Cat# HPA045943, RRID: AB_2679508
E-cadherin mouse anti-human antibody	Cell Signaling Technology	Cat# 14472, RRID: AB_2728770
Cleaved caspase-3 rabbit anti-human antibody	Cell Signaling Technology	Cat# 9664, RRID: AB_2070042
CD3 mouse anti-human antibody (IF validation)	Abcam	GeneTex Cat# GTX17143, RRID: AB_422603
IGG4 rabbit anti-human antibody (IF validation)	Abcam	Abcam Cat# 3479-1, RRID: AB_10702925

**Biological samples**		

Adult human colon resections, biopsies and blood samples	John Radcliffe Hospital NHS Foundation Trust, TIP consortium	REC reference(s): PRISE: 18/LO/0412, GI Biobank: 16/YH/0247, IBD Biobank: 09/H1204/30, TIP: 18/WM/0237 Sample overview detailed in Supplementary Data Table S1

**Chemicals, peptides, and recombinant proteins**		

OCT Embedding matrix for frozen sections	CellPath	Cat#KMA-0100-00A
Isopentane (2-Methylbutane)	Sigma	Cat#277258-1L
RPMI-1640 medium	Sigma	Cat#R8758-500ml
Dulbecco's Modified Eagle's Medium (DMEM)	Sigma	Cat#D5796-500ML
Penicillin-Streptomycin	Sigma	Cat#P0781-100ML
HEPES Buffer Solution (1M)	Gibco	Cat#15630-056
Fetal Calf Serum / Fetal Bovine Serum	Sigma	Cat#F9665-500ML
Lymphoprep	Serumwerk Bernburg	Cat#1858
Dimethyl Sulfoxide	Sigma	Cat#D8418-100mls
MEM Non-Essential Amino Acids	Sigma	Cat#M7145-100ml
Sodium Pyruvate Solution	Sigma	Cat#S8636-100mls
Vectashield Mounting Medium with DAPI	Vector	Cat #H-1200
Ultrapure 0.5M EDTA, ph8.0	Invitrogen	Cat#15575-038
HBSS medium	Lonza	Cat#10-543F
Pierce DTT (Dithiothretitol)	Thermo Scientific	Cat#A39255
Bovine Serum Albumin	Sigma	Cat#A7906-100G
Tryple Express	Gibco	Cat#12605-028
Lamina Propria Dissociation Kit, Mouse	Miltenyi	Cat#130-097-410
Mayer's Hematoxylin (used for ST)	Dako	Cat#S3309
Dako Bluing Buffer (used for ST)	Dako	Cat#CS702
Eosin Y solution	Sigma	Cat#HT110216-500ml
Normal Goat Serum 2.5%	ImmPRESS Vector	Cat#30023
Trueview Autofluorescence Quenching Kit	Vector	SP-8400
Phosphate Buffered Saline (PBS)	Oxoid Ltd or Sigma (experiment dependent)	Cat#BR0014G / D8537-500ML
CryostorCS10	Sigma	Cat#C2874-100ML
APC conjugation kit (Lightning-Link)	Abcam	Cat#ab201807-300ug
Dynabeads™ Human T-Activator CD3/CD28 for T Cell Expansion and Activation	Thermofisher	Cat#11161D
Nivolumab 40mg in 4ml Injection "Opdivo" (Packs of 1 vial)	Oxford University Hospitals Pharmacy	Opdivo
Fixation/Permeabilization Kit for FACS (Cytofix/Cytoperm)	BD biosciences	Cat #554714, AB_2869008
Recombinant Human Interferon gamma	Peprotech	Cat#300-02-20uG
Recombinant Human TNF alpha	Peprotech	Cat#300-01A-10uG
WNT conditioned medium	ATCC	CRL:2647TM
DMEM-F12 medium	Gibco	Cat#11320033
B-27 Supplement (50X), serum free	Thermo fisher scientific	Cat#17504044
Recombinant Human EGF	PeproTech	Cat#AF-100
*N*-acetyl-L-cysteine	Sigma Aldrich	Cat#A9165
Recombinant Human Noggin	Peprotech	Cat#120-10C
Recombinant Human R-Spondin-1	PeproTech	Cat#120-38
Human Gastrin-I	Sigma Aldrich	Cat#G9145
Nicotinamide	Sigma Aldrich	Cat#N0636
A83-01	R&D Systems	Cat#2939/10
SB202190	R&D Systems	Catalog#1264
CTS GlutaMAX-I Supplement	ThermoFisher Scientific	Cat#A1286001
HEPES solution	Sigma	Cat#H0887
N-2 Supplement (100X)	ThermoFisher Scientific	Cat#17502048
Y-27632 dihydrochloride	R&D Systems	Cat#1254/10
CellTrace™ CFSE Cell Proliferation Kit, for flow cytometry	ThermoFisher Scientific	Cat#C34554
CD3 Monoclonal Antibody (OKT3)	ThermoFisher Scientific	Catalog#14-0037-82

**Critical commercial assays**		

Visium Spatial Tissue Optimization Slide	10x Genomics	Cat#1000191
Visium Spatial Gene Expression Slide	10x Genomics	Cat#1000185
KAPA SyBR FAST qPCR kit	Kapa biosystems	Cat # KK4600
10x Chromium Single Cell 3' GEM, Library & Gel Bead Kit v3	10x Genomics	Cat#1000075
KAPA library quant kit (illumina) uiversal qPCR mix	Kapa biosystems	Cat# KK4824
QuBit dsDNA HS Assay Kit (used with QuBit 3.0)	Invitrogen	Cat#Q32851
High sensitivity RNA Screen Tape, Buffer and Reagents (for use with Agilent 2200 TapeStation system)	Agilent	Cat#5067-5579,5580 and 5581
10x Chromium Single Cell 5' GEM, Library and Gel Bead	10x Genomics	Cat#1000006
RNeasy Plus Micro Kit	Qiagen	Cat#74034
Novaseq 6000 S4 150bp PE reads	Illumina	Cat#20012866
Nextseq 500/550 Hi Output kit v2.5	Illumina	Cat# 20024907
High Sensitivity DNA reagents (Used with Agilent 2100 Bioanalyser system)	Agilent Technologies	Cat#5067-4626
CosMx SMI	Nanostring	

**Deposited data**		

Slide A1 and A2 (Spatial Transcriptomics), Raw data	Fawkner-Corbett et al.^60^	GEO ID: GSE158328 Mendeley Data (H&E Images): https://doi.org/10.17632/gncg57p5x9.2
Visium Spatial Transcriptomics	https://www.ncbi.nlm.nih.gov/geo/query/acc.cgi?acc=GSE189184	GEO: GSE189184
scRNA-Seq, CD45^+^ Cells	https://www.ncbi.nlm.nih.gov/geo/query/acc.cgi?acc=GSE189754	GEO: GSE189754
scRNA-Seq, CD3^+^ Cells	https://www.ncbi.nlm.nih.gov/geo/query/acc.cgi?acc=GSE189040	GEO: GSE189040
scRNA-Seq, epithelium, stroma, immune from biopsies + PBMCs	https://www.ncbi.nlm.nih.gov/geo/query/acc.cgi?acc=GSE190564	GEO: GSE190564
Mendeley data - raw image data, gene marker tables, differentially expressed gene tables, pathway analysis results, QC metrics summaries, featue barcoding antibody oligo tags.	https://doi.org/10.17632/7z8yx644hb.1	https://doi.org/10.17632/7z8yx644hb.1
scRNA-Seq colorectal cancer	https://www.ncbi.nlm.nih.gov/geo/query/acc.cgi?acc=GSE108989	GEO: GSE108989
scRNA-Seq liver cancer	https://www.ncbi.nlm.nih.gov/geo/query/acc.cgi?acc=GSE98638	GEO: GSE98638
scRNA-Seq and ST browsable data portal, with links to processed data Seurat objects	https://simmonslab.shinyapps.io/CPI_COLITIS_DATA_PORTAL/	https://simmonslab.shinyapps.io/CPI_COLITIS_DATA_PORTAL/

**Software and algorithms**		

FlowJo v10.7.1	FlowJo	FlowJo.com
Graphpad Prism v9.1.2	Graphpad	www.graphpad.com
Las X Version 3.7.4.23463	Leica Microsystems CMS GmbH	www.leica.com
QuPath v0.2.3	Github (open source)	https://qupath.github.io
Zen Blue Edition v3.3.89.0000 (ZEN lite)	Carl Zeiss Microscopy GmbH	www.zeiss.com
Visiopharm Integrator System (VIS) platform v 2019.07.3	Visiopharm	www.visiopharm.com
Biorender (Graphical Abstract)		Created with BioRender.com
fastQC version 0.11.9	https://www.bioinformatics.babraham.ac.uk/projects/fastqc/	
cellranger version 6.0.1	https://support.10xgenomics.com/single-cell-gene-expression/software/pipelines/latest/what-is-cell-ranger	
spaceranger version 1.2.2	https://support.10xgenomics.com/spatial-gene-expression/software/pipelines/latest/what-is-space-ranger	
bcl2fastq version 2.20.0.422	https://support.illumina.com/sequencing/sequencing_software/bcl2fastq-conversion-software.html	
R package DropletUtils version 1.8.0	https://bioconductor.org/packages/release/bioc/html/DropletUtils.html	
R package Seurat version 4.0.1	https://satijalab.org/seurat/	
R package Harmony version 1.0	https://github.com/immunogenomics/harmony	
R package Monocle3 version 0.2.3.0	https://cole-trapnell-lab.github.io/monocle3/	
R package ggplot2 version 3.3.2	https://ggplot2.tidyverse.org/	
R package DESeq2 version 1.28.1	https://bioconductor.org/packages/release/bioc/html/DESeq2.html	
R package ggpubr version 0.4.0	https://cran.r-project.org/web/packages/ggpubr/index.html	
R package miloR version 0.99.19	https://github.com/MarioniLab/miloR	
R package AUCell version 1.10.0	http://bioconductor.org/packages/release/bioc/html/AUCell.html	
pySCENIC version	https://github.com/aertslab/pySCENIC	
R package CellChat version 1.0.0	https://github.com/sqjin/CellChat	
R package SPOTlight version 0.1.0	https://github.com/MarcElosua/SPOTlight	
R package RCTD	https://github.com/dmcable/RCTD	
R package igraph version 1.2.5	https://cran.r-project.org/web/packages/igraph/index.html	
R package ggraph version 2.0.3	https://cran.r-project.org/web/packages/ggraph/index.html	
R package SingleCellSignalR version 1.0.0	http://www.bioconductor.org/packages/release/bioc/html/SingleCellSignalR.html	
R package MAST version 1.14.0	https://www.bioconductor.org/packages/release/bioc/html/MAST.html	
R package clusterProfiler version 3.16.0	https://bioconductor.org/packages/release/bioc/html/clusterProfiler.html	
R package org.Hs.eg.db version 3.11.4	https://bioconductor.org/packages/release/data/annotation/html/org.Hs.eg.db.html	
TRUST4	https://github.com/liulab-dfci/TRUST4	
R package divo version 1.0.1	https://cran.r-project.org/web/packages/divo/index.html	
GLIPH2	https://doi.org/10.1038/s41587-020-0505-4	
R package jcolors version 0.0.4	https://cran.r-project.org/web/packages/jcolors/index.html	
R package immunarch version 0.6.6	https://cran.r-project.org/web/packages/immunarch/index.html	
R package venneuler 1.1-0	https://cran.r-project.org/web/packages/venneuler/index.html	
VDJtools	https://github.com/mikessh/vdjtools	

**Other**		

Haematoxylin and Eosin images from all Spatial Transcriptomic sections	Zeiss Axioscanner	Supplementary data in Mendeley. https://doi.org/10.17632/7z8yx644hb.1
Immunofluorescence Images	Leica Widefield Microscope scanner Zeiss Axioscanner	Supplementary data table in Mendeley. https://doi.org/10.17632/7z8yx644hb.1

### Resource availability

#### Lead contact

Further information and requests for resources and reagents should be directed to and will be fulfilled by the lead contact: Alison Simmons (alison.simmons@imm.ox.ac.uk).

#### Materials availability

This study did not generate new unique reagents.

#### Data and code availability

Raw scRNA-seq, CITE-seq and hashing antibody data have been deposited at GEO:GSE189040 GSE189754 GSE190564.

Raw ST sequencing data have been deposited at GEO:GSE189184 GSE158328.

De-identified standardized patient information has been deposited at Mendeley Data: https://doi.org/10.17632/7z8yx644hb.1.

Microscopy images, including original spatial transcriptomic-paired Hematoxylin & Eosin images, as well as immunofluorescence images have been deposited at Mendeley Data: https://doi.org/10.17632/7z8yx644hb.1.

CITE-Seq and hashing antibody tag sequences have been deposited at Mendeley Data: https://doi.org/10.17632/7z8yx644hb.1.

Sequencing data QC metrics have been deposited at Mendeley Data: https://doi.org/10.17632/7z8yx644hb.1.

All analyzed scRNA-seq and ST data has been made available via an interactive data portal at https://simmonslab.shinyapps.io/CPI_COLITIS_DATA_PORTAL/. All processed data objects, cell/spot meta data and embeddings used in this publication and analysis code are available via the data portal and Mendeley data.

### Experimental model and study participant details

#### Human patients

Fully informed consent was taken from patients prior to collection of research samples attending Oxford University Hospital NHS Foundation Trust under the aegis of multiple protocols approved by NHS Research Ethics Committees (PRISE: 18/LO/0412, GI Biobank: 16/YH/0247, IBD Biobank: 09/H1204/30, TIP: 18/WM/0237). Patient demographic and treatment data are summarized in Table S1.

### Method details

#### Patient Sample collection

Samples were taken during clinically mandated visits in compliance with local and national patient safety directives, with informed consent. Some patients undergoing immunotherapy attended for the sole purpose of sample collection as part of the PRISE study protocol, however this was suspended after the start of the Covid-19 pandemic. Patients with suspected immunotherapy colitis were empirically and variably treated with steroids at the discretion of the admitting clinician, as per national guidelines (https://doi.org/10.1016/S2468-1253(20)30014-5). Consequently, initiation of treatment prior to confirmation of disease state at endoscopy was variable, and is reflected in the sample cohort, with both patients subsequently confirmed to have inflammation (CPI colitis) and those without colitis (CPI controls) receiving steroid treatment.

3-4 biopsy pairs were taken at endoscopy and 10mls of blood was collected at the same clinical visit. Surgical samples were collected by the operating surgeon from tissue in excess of clinical histopathological requirements. Inflammation status was confirmed through endoscopic assessment as well as after review of the clinical histopathology report issued by experienced consultant pathologists employed for NHS patient care. Anonymized individual participating patient details (including sex, medication and disease severity) have been deposited at Mendeley Data (accession: https://data.mendeley.com/datasets/7z8yx644hb/draft?a=e8b9e179-8fb7-448e-a481-7cd4c090f71a).

#### Sample Handling and processing

Tissue samples were transported on ice in DMEM supplemented with 10% Fetal Calf Serum (FCS, Sigma-Aldrich) and 100 U/mL of Penicillin and 100 ug/mL Streptomycin (Sigma-Aldrich), were washed in Phosphate-buffered saline (PBS) and frozen down immediately in CryoStor® cell cryopreservation media (CS10, Sigma-Aldrich) in order to be processed simultaneously reducing batch effects. Viability was similar to those of freshly isolated samples.

Blood was diluted in PBS and layered on a standard density gradient (Lymphoprep™). Peripheral Blood Mononuclear Cells (PBMC) were collected from the interface and washed in PBS. PBMCs were then resuspended in 10% DMSO 90% FCS and immediately transferred into a freezing isopropanol container (VWR; cooling rate of 1°C/min) for freezing, and stored at −80°C overnight. Samples were then transferred to a liquid nitrogen storage tank until required.

For cryosections, the mucosa with adherent submucosa was dissected away from surgical samples. Surgical and biopsy samples were washed in PBS, arranged in cryomolds containing OCT (Glycol and resin compound used for cryo-section tissue embedding), and then placed in an isopentane bath cooled to -80°C. Once solid, these embedded sections were placed in sealed containers at -80°C for storage until processing together for spatial analysis.

For Formalin-Fixed Paraffin-Embedded (FFPE) sections, biopsy samples were fixed in formalin for 48 hours before transfer to 70% ethanol and wax embedding through a standard graded series.

#### Cell isolation and staining from biopsies and blood

##### Epithelial/lamina propria/matched PBMC separation

Tissue dissociation was performed as previously described.^61^ Briefly, in order to enrich for the epithelial fraction, biopsies were defrosted, cut into small pieces in ice cold media, then agitated at 37°C in Hank’s balanced salt solution (HBSS, Gibco) supplemented with 5mM Ethylenediaminetetraacetic acid (EDTA, Invitrogen) and 2mM Dithiothreitol (DTT, ThermoFisher). The supernatant containing epithelial crypts was digested into a single cell suspension utilizing TrypLE™ Express Enzyme (ThermoFisher). Dead cells and debris were finally removed through filtering through 100uM and 40uM meshes. The remaining fragments of the biopsies (enriched for the lamina propria fraction) were dissociated using the Lamina Propria digestion kit (Miltenyi) for 1 hour. Debris and dead cells were removed as described above. For matched blood samples, frozen PBMCs were defrosted into cold media and washed three times with media before suspension in PBS supplemented with 2% Bovine Serum Albumin (BSA, Sigma-Aldrich) and 0.01% Tween (Sigma-Aldrich) (Staining buffer). Following separation, 0.5 million cells from each fraction (Epithelial, CD45/Stromal and PBMC) were incubated with a mix of optimized concentrations of hashing and CITE-seq antibodies for 30 minutes at 4dC after blocking with Trustain FcX (Biolegend) for 10 minutes. At each step cells were washed in staining buffer to remove unbounded antibodies.

##### CD45^+^ enrichment

The entire biopsy was digested using the Lamina Propria digestion kit (Miltenyi), and dead cells and debris were removed by filtering through 100uM and 40uM meshes. Following dissociation, 0.5 million cells from each patient were incubated for 10 minutes at 4°C with Trustain FcX (Biolegend) to block non-specific binding, followed by 30 minutes at 4°C with anti-CD45 (APC, 5B1, Miltenyi) and anti-CD236 (FITC, HEA-125, Miltenyi) in order to stain CD45^+^ cells. DAPI (4′,6-diamidino-2-phenylindole, BD) was added at 1ug/ml for live-dead differentiation just prior to sorting. Samples were acquired immediately afterwards on a BD FACSAria III Cell Sorter (BD FACS Diva Software). The accuracy of sorting was confirmed using beads and cells. Cells were gated based on size using Forward and Side scatter, followed by identification of singlets using FSC-H and FSC-A. After gating on Live cells, CD45^+^ cells were sorted. For each sample, 100,000 live single cells were sorted into Eppendorfs containing PBS supplemented with 2% Bovine Serum Albumin (BSA, Sigma-Aldrich) and 0.01% Tween (Sigma-Aldrich). Cells were spun down, counted, resuspended at the desired concentration and immediately loaded on the 10X scRNA-seq platform.

##### CD3^+^ enrichment from tissue and blood

The entire biopsy was digested using the Lamina Propria digestion kit (Miltenyi) , and dead cells and debris were removed by filtering through 100uM and 40uM meshes. PBMCs from matched frozen samples were defrosted into cold medium. Following isolation of cells from biopsy and blood, 0.5 million cells from each patient were incubated for 10 minutes at 4°C with Trustain FcX (Biolegend) to block non-specific binding, followed by 30 minutes with anti-CD45 (APC, 5B1, Miltenyi) and Anti-CD3 (PE-Dazzle594, UCHT1, Biolegend) along with a mix of optimized concentrations of hashing and CITE-seq antibodies. DAPI (4′,6-diamidino-2-phenylindole, BD) was added at 1ug/ml for live-dead differentiation just prior to analysis. Samples were immediately sorted on a BD FACSAria III Cell Sorter (BD FACS Diva Software). The accuracy of sorting was confirmed using beads and cells. Cells were gated based on size using Forward and Side scatter, followed by identification of singlets using FSC-H and FSC-A. After gating on Live cells, the target population was sorted for CD3^+^ cells. For each sample, 100,000 live single cells were sorted into Eppendorfs containing PBS supplemented with 2% Bovine Serum Albumin (BSA, Sigma-Aldrich) and 0.01% Tween (Sigma-Aldrich).

#### Proliferation analysis – CFSE staining

A 96-well plate was treated to create a OKT3 titration series from 1-0.0016ug/ml. Previously isolated PBMCs from healthy controls were defrosted, rested overnight and incubated at 75,000 cells/well. Cells were stained with for CD3 (AF700), CD4 (FITC), CD8 (BV421), CD25 (APC-Fire 750), PD-1 (PE, Clone EH12.2H7) and non-comp PD-1 antibody (AF647) along with viability dye (Zombie Aqua) and CFSE. These cells were either incubated with medium or Nivolumab at 10ug/ml. FACS analysis of harvested cells was carried out at 5 days.

#### Organoid cultures

Organoid cultures were established as previously described.^62^ In brief, cultures were established from the chelated epithelial fraction from four pairs of colonic biopsies (as previously described for single-cell RNA sequencing). This was then mixed with 50 μl Matrigel (Corning) and plated on prewarmed 24-well culture dishes. Embedded cells were overlaid with WREN conditioning medium (WNT3A conditioned medium, containing lipid-modified WNT3A and ADF (advanced DMEM-F12 medium) 50/50, glutamax, 10 mM HEPES, N-2 supplement (×1), B-27 supplement (×1), 10 mM nicotinamide, 1 mM N-acetyl-L-cysteine, 1 μg ml−1 R-spondin 1, 50 ng ml−1 human epidermal growth factor, 100 ng ml−1 human Noggin, 1 μg ml−1 gastrin, 0.1 μM A83-01, 10 μM p38 inhibitor SB202190, and 10 μM Y27632 (until the first medium change), supplemented with Pen-strep and Normacin anti-microbials. Medium was replaced with fresh WREN medium every other day. Differentiation medium was also changed every day and constituted as above with a lower WNT3A concentration (10% of WREN CM), 125 ng ml−1 human epidermal growth factor and without SB202190 or nicotinamide.

For organoid-stimulation experiments, once organoid cultures were established and a first passage performed, 10 ng ml−1 interferon-γ and TNF-α were added to medium every 48 hours at the time of medium change. For gene-expression quantification, we isolated RNA from organoids using the Qiagen RNA micro kit and Taqman assay-based RT-PCR (GAPDH and RPLP0 housekeeping genes).

#### Single-cell RNA sequencing

##### Epithelial/lamina propria/matched PBMC cells

Following cellular isolation and staining (described above), cells from up to five different samples were pooled at 1:1 ratio. Each pool was loaded on a channel of the 10X Chromium single-cell platform, one for the epithelial fraction and the other for the lamina propria fraction. An input of 30,000 single cells per pool was added to each channel with a recovery rate of approximately 10,000 cells. Libraries were prepared using 10x Genomics Library Kits (10X Genomics, CG000186,Rev A). From matched blood samples, cells from four different samples were pooled at 1:1 ratio. For each pool 30,000 cells were added to each channel. Gene expression (GEX) and Antibody-derived Tag (ADT) Libraries were prepared using 10x Genomics Library Kits (10X Genomics, CG000186,Rev A). In addition, single-cell TCR and BCR libraries were generated from the lamina propria and PBMC pools.

##### Tissue CD45^+^ cells

Live CD45^+^ cells were sorted as described above, counted and resuspended at a final concentration of 1million/mL. For each sample, 10’000 cells were loaded into the 10X scRNA-seq platform. GEX Libraries were prepared using 10x Genomics Library Kits (10x Genomics, CG000183,Rev A).

##### CD3^+^ cells

Live CD3^+^ cells from tissue and blood were sorted as described above. Cells from three samples were counted, pooled at a 1:1 ratio, and 20,000 cells from each pool were loaded into the 10X scRNA-seq platform. GEX, ADT and TCR libraries were prepared using 10x Genomics Library Kits (10x Genomics, CG000208,Rev F).

Sequencing of libraries was carried out on an Illumina NovaSeq 6000 platform with 1% PhiX (Novogene).

#### Spatial Transcriptomics

##### Visium 10X protocol

RNA was extracted from samples and a selection of representative cryosections using the RNEasy plus Micro RNA kit (Qiagen). RNA quality and quantity were assessed using a high sensitivity RNA ScreenTape assay in a 4200 TapeStation (Agilent Technologies). All the samples retained a RNA Integrity Number (RIN) > 8.5. The 10X Visium system was utilized for matched H&E (Hematoxylin and Eosin) and Spatial Transcriptomics information. Tissue permeabilization was optimized for RNA extraction from inflamed sections using the Visium Spatial Gene Expression Kit (CG000238 Rev D; Imaged on a Leica DMI8 inverted microscope). Briefly, 10uM thick serial sections from an inflamed biopsy sample (Nancy Score 4) on a tissue optimization slide were incubated with permeabilization enzyme from a range of 3-24 minutes as per the described protocol (CG000238 Rev D) to determine the optimum tissue digestion time. The timepoint which yielded an optimum trade-off for RNA recovery across epithelium and stroma was chosen for tissue digestion for all samples. 10uM sections from inflamed sections and healthy controls were placed onto slides and processed to yield H&E sections (imaged at 10X on a Zeiss Axioscan z.1 slidescanner). These sections were then processed for spatial transcriptomic libraries at a resolution of 55uM per spot (Visium 10X, CG000239,Rev D). Libraries were sequenced on an Illumina NextSeq platform.

##### CosMx high resolution spatial transcriptomics

We utilized this technology prior to its widespread commercial availability through the Nanostring SMI Technology Access Program. The exact protocol is currently proprietary, but briefly, a company-developed standardized panel of probes was used on an adapted GeoMx protocol for data generation. Both IgG4 antibodies validated for detecting bound cells at immunofluorescence (IF, see below) were tested for detecting bound cells on this platform by using a company-provided protocol for slide preparation (requiring deparaffinization, antigen retrieval, proteinase digestion, post-fix preservation, and morphology marker incubation), followed by standard imaging using IF. We determined that the HP6025 anti-human IgG4 clone could successfully detect the bound cell epitope despite the proteinase digestion step required prior to tagged RNA probe detection via CosMx. Freshly cut 4uM sections of patients were then sent on ice to Nanostring for blinded data generation via their TAP programme, with delivery of raw data back to us.

#### Immunofluorescence

Deparaffinized 4uM sections from biopsies were subjected to heat-mediated antigen retrieval at pH 9 in Tris-EDTA buffer for 30 minutes, followed by a 1 hour blocking with goat serum (Vector) at room temperature. Sections were incubated with optimized dilutions of primary antibodies to the appropriate antigens ( FOXP3 - HPA 045943, 1 in 4000 dilution; FABP1 - HPA028275, 1 in 800 dilution; CD163 - Ed-Hu1, 1 in 200; CD3 – D76AE, 1 in 200 dilution; IgG4 – HP6025, 1 in 500 dilution; CD3 – F7.2.38, 1 in 50 dilution; IgG4 – EP4420, 1 in 2000 dilution), in PBS supplemented with 1% BSA at 4°C overnight, followed by secondary goat antibodies (1 in 500 dilution) conjugated to AF488 and AF647 fluorochromes for 1 hour at room temperature (RT) in 1% BSA/PBS ^+^ 1ug/ml DAPI. Following this, slides were incubated with Vectashield Truview (Vector) for 4 minutes to reduce autofluorescence. Each step was separated by multiple washes in PBS/PBS-Tween. Slides were imaged at 20X on a Zeiss Axioscan z.1 slide scanner within 24 hours of staining, being kept at 4°C until acquisition. We developed a standardized exposure and acquisition protocol that was used across all sections. A control ‘secondary antibody only’ section was used to correct for autofluorescence prior to image export at >75% original size and resolution JPEG/TIF format used for image analysis.

#### Quantification of lymphoid follicles

Histopathological analysis was carried out in a blinded fashion by an experienced consultant gastrointestinal pathologist who routinely assesses colonic inflammation (E.F.) Briefly, deparaffinized sections from a randomly selected proportion of patients with checkpoint inhibitor induced colitis, ulcerative colitis and health were stained utilizing a Vector kit and standard hematoxylin and eosin protocol (Materials). Brightfield images at 10X were acquired with a Zeiss Axioscanning widefield microscope. Anonymized images were analyzed by the histopathologist to quantify the number of lymphoid follicles. The data was corrected for image area (calculated using the area tool on opensource ImageJ software) and analyzed, generating figures utilizing GraphPad Prism software.

#### FACS validation of CPI-bound T cells

The non-competing PD-1 antibody (conjugated to AF647) had been previously validated to bind at a site on PD-1 more proximal to Nivolumab that did not interfere with Nivolumab binding. To verify that Nivolumab interfered with the binding of the commercial CITE-seq antibody (Clone EH12.2H7) but did not interfere with the binding of the non-competing PD-1 antibody (Noncomp PD-1), the following protocol was used. Healthy colonic biopsy samples were digested as described above for scRNA-Seq and then stimulated with anti-CD3/CD28 beads (10uL/1 million cells) for 18 hours. Following washing, the sample was split into three – one half was incubated with Noncomp PD-1 and CITE-seq antibody (at concentrations of 10ug/ml) and the other half with Noncomp PD-1, CITE-seq antibody and unlabeled Nivolumab (all at concentrations of 10ug/ml) for 30 minutes at 4°C. The samples were then run on an LSRII analyzer with isotype controls for Noncomp PD-1 and CITE-seq antibody at 10ug/ml each to assist with gating. The third fraction was incubated with Nivolumab conjugated to APC (data not shown) and demonstrated that the proportion of cells binding Nivolumab was the same as those binding Noncomp PD-1.

To verify that Nivolumab was still bound to cells from patients given checkpoint inhibitors *in vivo*, colonic biopsy samples from patients with checkpoint inhibitor induced colitis were digested as specified above, and then incubated with Noncomp PD-1 and CITE-seq antibody (at 10ug/ml) for 30 minutes at 4°C. The samples were then run on an LSRII analyzer with isotype controls for Noncomp PD-1 and CITE-seq to assist with gating.

#### Image analysis

Quantification of the appropriate signal was done on images by a researcher (NK.A) blinded to the identity of the samples. The n for each condition indicates the number of patients scanned, and are elucidated in the figure legends. The data are presented as Mean, with error bars denoting Standard Error of Mean (SEM). Digitized slides were analyzed using the Visiopharm Integrator System (VIS) platform (v 2019.07.3). Image analysis protocols were implemented as Analysis Protocol Packages (APP) in VIS. Several APPs were designed using threshold classification to quantify the slides. Data was analyzed using GraphPad Prism software, using an unpaired t-test for non-parametric data (Kolmogorov-Smirnov test), with significance being defined as p <0.05. Data are presented as mean with error bars denoting standard error of mean.

##### FABP1-associated CD163 DAPI analysis

From previously published data as well as our own transcriptomic analysis, we determined FABP1 was upregulated in a graded fashion from the crypt base to the crypt top. We first validated this in immunofluorescence of longitudinal sections of crypts, and standardized the fluorescence signal intensity to distinguish crypt tops from bases across sections, in order to be able to analyses sections where the orientation was not optimal. For each section, utilizing this intensity of signal, we determined whether the crypt area detected was from crypt base, mid-crypt or crypt top. For each area (crypt base, mid-crypt and crypt-top), we designated a standard ‘test zone’, which was delineated by half the average distance between crypts in pixels (to avoid double counting cells next to adjacent crypts), annotating this as a ‘peri-crypt zone’. Within this test peri-crypt zone, we then counted the area positive for CD163 (FABP1-high crypt top, mid-crypt or FABP1-low crypt base M2 macrophages), correcting for total area using DAPI as before.

##### FOXP3-associated CD163 macrophages

FOXP3 presented a challenge in detection given its relative paucity, which meant even with autofluorescence quenching, small false positive spots were present in each tissue. To circumvent this, we utilized a data analysis approach that ignored such false positives. Briefly, around each CD163-positive area (M2 macrophage) we designated a standard ‘test zone’ double the radius of an average M2 macrophage (reasoning this represented a maximum diameter within which cells could be expected to be interacting, with any secreted cytokine approaching ∼1/50^th^ of the concentration at the center). Within this ‘test zone’, we only counted a FOXP3-positive Treg cell if the FOXP3 signal was wholly ensconced within a halo of DAPI (i.e. intranuclear), discounting false positive autofluorescence that did not obey nuclear boundaries. Analysis was again carried out with correction for DAPI-derived section area.

#### Raw sequencing data processing

All raw sequencing data was converted to from bcl to fastq format using Illumina bcl2fastq software, version 2.20.0.422, with allowing up to one mismatch in each sample index barcode. Raw sequence reads were quality checked using FastQC software.^63^

#### Raw 10X scRNA-Seq, CITE-Seq and spatial transcriptomics data processing

For each sequenced scRNA-Seq pool, Cellranger software from 10 × Genomics (https://support.10xgenomics.com/single-cell-gene-expression/software/downloads/latest) was used to process, align and summarize unique molecular identifier (UMI) counts against hg38 (10x reference: refdata-gex-GRCh38-2020-A) human reference genome. Matched protein CITE-Seq and hashing antibody panel data were processed together with scRNA-Seq as matched feature barcoding libraries. Antibody tag UMI counts were summarized using a joint feature barcoding sequence reference of TotalSeq antibody sequences of hashing antibodies and protein expression target panel. Feature barcoding reference tag sequences are provided in **Mendeley Data**.

#### 10x scRNA-Seq data analysis

Raw UMI count matrices were imported into R for further processing. For each scRNA-seq sample, cell calling was performed using ‘emptyDrops’^64^ function from DropletUtils on the full raw count matrices of all barcodes in the 10x barcode whitelist to distinguish cells from empty droplets containing only ambient RNA. Raw count matrices were corrected for Illumina index swapping using ‘swappedDrops’.^65^

Furthermore, droplet barcodes for which a high percentage of total UMIs originated from mitochondrial RNAs were filtered out, as well as low total UMI count barcodes. These thresholds were derived individually for cells within each compartment following an initial clustering solution of all cells by examining and thresholding empirical distributions within each compartment, as total RNA content (notably higher in endothelial and myeloid cell populations) and mitochondrial RNA content (notably higher in epithelial cells) are highly cell type dependent.

For each individual 10x reaction, Seurat R package^66^ was used to normalize expression values for total UMI counts per cell. Highly variable genes were identified by fitting the mean-variance relationship and dimensionality reduction was performed using principal-component analysis. Scree plots, which visualize the amount of variation in the data accounted for by each principial component were used to determine the number of principal components to use for clustering analyses for each pool. Cells were then clustered using Louvain algorithm for modularity optimization using kNN graph as input. Cell clusters were visualized using UMAP algorithm^67^ with principal components as input and n.neighbors = 30, spread = 1 and min.dist = 0.1.

Cells from separate pools/reactions were merged together within the same reaction/isolation type (e.g., all epithelial crypt dissociation pools vs all whole PBMC reactions) and the pool batch effect signal was corrected using the harmony algorithm.^68^ This enabled batch correction of individual 10x reaction technical background/ambient RNA effects without loss of condition-specific signals as each set of samples was pooled in hashed reactions in such a way as to reduce condition-batch confounding where possible. Where samples from different isolation protocols were merged for further analysis (e.g., T cells from CD3^+^, CD45^+^ or total non-epithelial fraction cells), harmony was used to correct for both 10x reaction and isolation protocol effects, using equal weighting/parameters for both batch variables.

In each case, merged cell clustering and visualization of cells was performed as before using Louvain and UMAP algorithms, using harmony dimensionality reduction as input instead of principal components. Merged pool clusters were compared with cell types obtained from individual pools to ensure cell type heterogeneity was not lost due to batch correction.

For CITE-Seq protein expression analysis, count matrices were imported into R as before as a separate assay in Seurat objects and filtered to retain only those cells passing QC based on RNA expression analysis. Count data were normalized using a centered log ratio transformation within each reaction type separately (CD3^+^ FACS sort, PBMCs and non-epithelial fraction of crypt dissociation protocol), as the quality of staining differed between reaction types, with sorted T cell (the majority of T cells in this analysis) samples yielding the most distinct tag profiles. CD4 and CD8 antibody tag data were then further used to classify T cells into CD4, CD8 or double positive lineages, with normalized expression tag density distributions for each reaction type used to define cut offs for positive and negative staining. While the majority of CD8 and CD4 T cell subpopulations cluster separately on RNA expression alone, certain phenotypes (e.g., Tc17 and Th17 cells) are more difficult to differentiate and these clusters were split based on antibody expression classifications. We further confirmed the validity of this grouping by comparing shared TCR clonotype landscape, with CD8^+^ Tc17 cells showing more clone sharing with other CD8^+^ populations (as described in the *TCR Analysis* section below).

scRNA-seq cell populations were annotated using a combination of known marker gene and protein expression profiles (summarized in Table S2 for each population/cluster) and using previously published scRNA-seq reference atlas datasets in the colon and PBMCs.^6^^,^^7^^,^^8^^,^^51^^,^^61^^,^^69^ In the case of the latter, we carried out label transfer between datasets for each cell type in reference 10x datasets for each cell in our data using Seurat and assigned putative cluster identities to populations based on the most frequently predicted cell label for each cluster. Classification results were then checked against known marker gene expression. Finer/higher resolution clusters or condition-specific populations were denoted by their most specific or informative gene marker.

Similarly, we also carried out a label transfer procedure to assign a phenotype to cycling cells in G2M and S phases, which typically segregate into their own clusters and may be identified by G2M and S phase markers (e.g., *MKI67*). Firstly, in heterogeneous cell type reactions (e.g., non-epithelial or CD45^+^ isolations), we subset all non-cycling cell clusters and used these data as a reference to classify cycling cells into broad populations. Classification labels, which we further checked against known marker gene expression within each predicted cell group, were then used to separate cycling cells and take them forward for individual subset analysis (e.g., T cells). For T cell analysis, in order to analyses the phenotypes of proliferating T cells, we repeated the procedure by separating cycling and non-cycling T cells and used non-cycling T cell clusters as reference for label transfer. Similarly, to analyses the phenotypes of intra-epithelial T cells, we subset the “contaminating” T cell cluster recovered as part of the epithelial crypt dissociation reactions and classified it using the annotated cluster reference of all other T cells, the majority of which were recovered from location-agnostic CD3^+^ FACS sorted populations from whole biopsies.

Trajectories on T cell embedding were calculated using Monocle 3 algorithm^70^ using integrated, batch corrected, dimension-reduced data as described above. Seurat objects were converted to Monocle 3 cell_data_set objects and cells were re-clustered to enable learning of disjoint graphs in multiple partitions for CD4^+^ and CD8^+^ populations separately. Trajectory reconstruction was carried out allowing for closed loops and multiple partitions. The start of the trajectory was denoted as the node within the naïve cell cluster and pseudotime was computed along the trajectory. To calculate cell densities over pseudotime for TRMs -> reactivated TRMs part of the trajectory, cells along the branch of trajectory were subset and density distribution was calculated and visualized using gaussian density kernel estimate as implemented in ggplot2 function stat_density(), with default parameters.

Genes varying with pseudotime were detected by fitting generalized linear negative binomial models to each gene in cells within the TRM-> reactivated TRM part of the trajectory. To test for non-linear dependence of gene expression on pseudotime, we constructed natural splines (3 degrees of freedom) for pseudotime variable and used a likelihood ratio tests between full and reduced (no pseudotime) models to detect pseudotime varying genes. p-values were further corrected for multiple testing using Benjamini-Hochberg multiple testing correction. In order to visualize selected pseudotime varying gene expression patterns over pseudotime, for each gene across all cells within the TRM branch of the trajectory we generated a loess regression fit of expression over pseudotime. Smoothed expression patterns across 100 bins of pseudotime values obtained from the fits were visualized as heatmaps using R package ‘pheatmap’. Genes (but not pseudotime bins) were clustered using hierarchical clustering, with complete linkage.

Sample PCA analysis was carried out as follows. UMI raw count matrices of all confidently de-hashed cells passing QC were used to calculate pseudobulk counts for each individual sample by summing across all UMI counts for each gene for each cell for a given sample. Immune (T cells, B cells, Plasma cells, Mast cells, Myeloid cells) and non-immune (fibroblasts, myofibroblasts, endothelium, pericytes) cell clusters were separated in mixed reactions and analyzed separately. For epithelial crypt dissociation reactions, we only considered epithelial lineage (EPCAM^+^ cell clusters) cells, excluding all immune cell clusters to avoid capturing differences in immune infiltration levels that may mask transcriptomic differences within epithelial cells. Count data were normalized using sample normalization size factors which were computed using the median ratio method as implemented in DESeq2^71^ R package. A variance-stabilizing transformation was further applied via vst() function in order to reduce heteroskedasticity of the data. The top 1000 most variable genes were then selected and the normalized, transformed expression values for each sample were used to compute PCA.

To identify condition specific clusters, for each cluster for each sample within each cellular compartment we normalized cell counts to the total number of cells detected within that compartment in a given sample and the proportions of cells were compared using a two-sided Wilcoxon test, with p-values < 0.05 considered as significantly different. Proportion distributions, cluster means and standard errors were visualized using R package ggpubr. Differential abundance was further tested using sccomp R package.^72^ For cell type populations which exist on a phenotypic continuum rather than discrete clusters (e.g., T cell subtypes), we further carried out graph-based differential abundance analysis using R package miloR,^73^ using integrated, batch-corrected harmony components for nearest neighbor graph reconstruction, with k=10 nearest neighbors used for neighborhood definitions throughout.

The R package SCENIC workflow was used to detect active transcription factor modules in all datasets. The normalized single-cell gene expression matrix was first filtered to exclude all genes detected in fewer than 20 cells. The RcisTarget database, containing transcription factor motif scores for gene promoters and transcription start sites for the hg38 human reference genome, was downloaded from https://resources.aertslab.org/cistarget/databases/homo_sapiens/hg38/refseq_r80/mc9nr/gene_based/, and the expression matrix was further filtered to include only genes available in the RcisTarget database. The remaining genes were used to compute a gene–gene correlation matrix for co-expression module detection using the random forest-based GENIE3;^74^ the R package SCENIC^75^ was used to perform transcription factor network analysis to detect co-expression modules enriched for target genes of each candidate TF from the RcisTarget database. The AUCell package was used to compute a score for each TF module in each individual cell. In order to identify condition- or cluster specific TF modules, we used generalized linear models to test for condition or cluster dependence of TF AUC values, including batch and gene detection rate (as we found that AUC values are significantly dependent on gene detection rate of individual cells) as blocking co-variates in the model formula. Resultant p-values were further adjusted for multiple testing using Benjamini-Hochberg multiple testing correction.

Receptor-ligand interactions between all single cell clusters identified from scRNA-seq data as well as condition-specific interactions were inferred using R package ‘CellChat’.^76^ Cell-to-cell crosstalk using CellChat was done on each condition separately and then merged different CellChat objects for conditions together to model communication probability and significant communications between conditions. Normalized data matrix and cluster label metadata was provided as input to compute probability and infer cellular communication networks using CellChat functions “computeCommunProb”, “identifyCommunicationPatterns”, “computeCommunProbPathway”, “aggregateNet” and “compareInteractions”, with default parameters. Circos plots were used to visualize specific interactions.

#### Hashed sample de-multiplexing and doublet removal

Hashing antibody UMI count matrices were filtered to keep only 10x cellular barcodes from droplets passing QC based on mRNA expression profiles, as described above. Non-hashing antibody counts and hashing tags not present within any given pool (as hashed sample numbers varied between reactions between 3-5) were also filtered out for each pool individually. Each filtered matrix was used to demultiplex samples as described in.^77^ Counts were first normalized using centered log ratio transformation and an initial clustering solution was obtained using clara k-mediods clustering with k = 1 ^+^ number of samples in the pool. A negative binomial distribution was then fit for each tag and a positive tag threshold was defined as 99th percentile of the normalized UMI counts, with cells below this threshold considered negative for the tag. Cell sample-of-origin was then assigned for each cell based on individual hashtag thresholds with doublets/multiplets defined as cells positive for multiple tags and filtered out from further analysis. Due to random mixing, provided a sufficiently large number of samples are mixed together, the majority of doublets in the sample pool will be between-sample doublets and therefore can be filtered out from the data using this experimental labelling technique. A minor fraction of all cells were found negative/below tag threshold for all hash tags and were also filtered out, following inspection of their mRNA-cluster distributions. Untagged cells correlated with lower total mRNA content cells and did not segregate with any particular cluster and thus likely contained unstained/dying cells or free nuclei that have lost their cytoplasm during sample processing. Demultiplexed cells were visualized as tSNE plots from Euclidian distance matrixes. In each case, we then further examined whether sample demultiplexing was correct by ascertaining that the expression of sex-specific genes, such as *XIST*, segregated correctly with sample-of-origin assignments.

#### Spatial transcriptomics data analysis - Visium

Raw UMI count spot matrices, images, spot-image coordinates and scale factors were imported into R. Spot matrix was filtered out to keep only spots overlaying tissue sections. We next fit a negative binomial distribution to total UMI counts in ST spots not under tissue sections to determine the expected recovery of UMI spots in non-tissue/technical background spots. We then additionally filtered out all under-tissue ST spots with low RNA content where total UMI recoveries were consistent with non-tissue spots, as these areas in tissue were likely under permeabilized. The majority of spots filtered out this way were either section-specific or corresponded to tissue artefacts. Additional tissue artefacts (e.g., folds, tears) were further annotated in H&E and ST spots directly covering these regions were also excluded from further analysis. Spots under cover slip air bubbles were retained as these impacted only H&E image and were not considered tissue artefacts (and are denoted by ‘^∗^’).

Raw UMI spot counts were then normalized using regularized negative binomial regression (SC Transform)^78^ to better account for variability in total spot RNA content. Dimensionality reduction was performed using PCA and for each slide, scree plots were examined the determine the optimum number of principal components to use in downstream clustering analyses.

Clustering was performed using Louvain clustering algorithm as before (resolution = 0.5) and clusters were visualized using UMAP algorithm as before. Clusters distributions were visualized in spatial context over H&E images with spot size scaling factor of 1.6 used throughout.

For integrative data analysis, spots from individual slides were integrated using harmony algorithm correcting for slide-specific effects and clustering on merged dataset was carried out as before, except using harmony reduced dimension components instead of PCA. Merged data clusters were compared with those obtained from individual slides to ensure no heterogeneity was lost due to batch correction. Conversely, we examined individual slide contribution to integrated regions to ensure that equivalent regions between different tissue sections were clustering together. The majority of integrated mucosal regions showed contributions from all ST slides, however as expected submucosal regions had more contributing spots from deeper resection slides than biopsies, which tended to be shallower with only few submucosal spots. Furthermore, two transcriptionally diverse sub-mucosal regions and one deep tissue neural plexus region was captured only in two resection slides which covered deepest tissue regions. These clusters showed substantial biological pathway divergence from less deep submucosal regions and were observed only in deep tissue near vessel regions and thus are unlikely to be technical artefacts from insufficient batch correction, but rather section-depth related transcriptional heterogeneity.

For the majority of the transcriptome driven clusters, integrated ST slide clusters corresponded to H&E image features (e.g., muscularis mucosa, deep crypts, crypt tops, vessels, neural plexus) and these spot groups were annotated based on their ST structure/region accordingly. Two smaller clusters however were denoted based on the dominant cell type transcriptomic signature (macrophage and neutrophil rich clusters). All region annotations in H&E images were carried out in consultation with a specialist gastrointestinal cellular pathologist (EF) who was blinded to associated gene expression and associated derived data.

Due to the nature of biopsy tissue sections, within any given ST slide, there may be substantial variation between section cut type and directionality through the biopsy section – e.g., longitudinal vs cross-sectional, straight vs angled. In some cases, it is difficult to determine the depth of epithelial crypts in cross-sectional tissue cuts from histology alone. We additionally scored each ST spot using transcriptomic epithelial crypt axis score as described in,^61^ allowing to assign a numerical crypt depth value to each individual ST spot regardless of cut type. Crypt axis score vs actual tissue depth was determined by histology of longitudinal cut resection ST slides. Specific analyses of upper crypt spots were then carried by sub-setting all ST slides for spots with crypt-axis score > 1.0 (and confirmed by visual examination of H&E images where possible), with the cut-off determined based on score distribution in longitudinal cut resection slides to select spots restricted to the topmost two-spot depth upper crypt layer.

Cell type prediction probabilities were calculated for each spot using factor analysis via FindTransferAnchors and TransferData functions in Seurat using scRNA-seq data as reference datasets. scRNA-seq reference data from separate compartments (epithelial, stromal and tissue immune, but not PBMC) were merged and batch corrected as previously described into a unified reference dataset. We retained two levels of cell type annotations – broad populations (e.g., T cells) and fine sub-clusters (e.g., Naïve T cells, Tregs, Tfh, etc.). For broad population cell type predictions, we aligned the first 30 components of scRNA-seq and ST datasets, while the first 60 components were used for predictions of finer cell type sub-populations as more components were required to adequately capture transcriptional networks delineating finer grouping such as T cell subpopulations. To ensure that slide-to-slide variation did not skew single cell type spot composition analysis between conditions, we scored spots for single cell type composition using the integrated components in addition to uncorrected slide by slide basis. We further compared these results with two other ST single cell composition analysis approached, Cell2location,^79^ SPOTlight^80^ and RCDT^81^ to ensure cell type predictions were robust and there was agreement between different methods.

To identify spatial region cluster and condition specific cell type signal enrichment, we prioritized all cell type signatures by fitting generalized linear models, testing the dependence of cell type signature probability/decomposition scores on spatial region cluster vs all other spots, or between conditions. These data were visualized using dot plot heatmaps, where a spot mean probability was computed and scaled within groups for visualization. To compute the fraction of spots positive for a given cell type signature, positive spots were classified as those with cell type prediction probability > 0.

For cell type co-occurrence analysis, in order to broadly assess the spatial co-localization of cell populations within the same spots, we calculated all pairwise cell type prediction probability score correlations across all slides in UC, CPI-Colitis and HC conditions. To visualize cell type co-localizations in the three conditions, undirected, edge-weighted cell type networks were constructed from the correlation matrix from spots in each condition, retaining only significantly (p < 0.01) positively correlated cell type pair edges using R package ‘igraph’,^82^ with correlation values as edge weights. Diagonals (cell type signal correlation to itself) were also filtered out. Due to the large number of cell types, for visualization clarity we further filtered out edges below r < 0.15 to remove low correlation edges from the graphs across all conditions. Networks were visualized using R package ‘ggraph’, using force-directed Fruchterman-Reingold layout. The range of edge weights/widths and node sizes were standardized between visualizations for UC, CC and HC conditions.

To identify region-specific spatially co-localized cellular signaling events, we first downloaded receptor-ligand databases from^76^^,^^83^ and scored all individual ST spots for receptor-ligand co-expression as follows. As individual ST spots have lower gene detection rate than scRNA-seq, for each ST spot we also considered weighted receptor and ligand expression in surrounding spots. First, all pairwise Euclidean distances for all ST spots in all slides were computed and for each spot, a proximity-based linear weight was assigned to all other spots, with distal spots further than two immediately surrounding spots away assigned a weight of zero (no contribution towards co-localizing receptor-ligand score) while other surrounding spots were assigned a distance-normalized weighting between >0 and 1. Then, for each spot a distance-weighted, local region smoothed receptor-ligand product score is calculated, which is further scaled to total non-zero weight spots to account of edge of tissue cases:

$$\frac{\sum_{i=1}^{n}{\mathrm{d}}_{i}L_{i}}{n}\;.\;\frac{\sum_{i=1}^{n}{\mathrm{d}}_{i}R_{i}}{n}$$

Where L is ligand gene expression, R is receptor gene expression, d is distance-based weight and n is the number of spots with distance weight > 0. Next, we randomly shuffle all spot locations across all slides using 100 permutations and re-calculate the scores to compute an empirical background distribution that would be expected if there was no location specificity of receptor-ligand co-expression. Then for each spot and each receptor-ligand pair, we compute a p-value based on the empirical background distribution. A multiple testing Benjamini-Hochberg correction is further applied to control false discovery rate as all receptor-ligand pairs are tested; spots with <5% FDR were then considered as positive for a cross-talk via a given receptor-ligand pair.

To priorities region specific cross-talk events, we used generalized linear modelling, modelling receptor-ligand score dependence on each spatial region cluster, compared to spots in all other clusters and blocking for individual spot gene detection rate to account for variation in recoveries/permeabilization effectiveness between/within different slides. Similarly, condition-specific interactions were also modelled. We tested all receptor-ligand pairs which were detected as significantly co-localizing in at least one spot in a tested region/condition. Benjamini-Hochberg multiple testing correction was further applied to control false discovery rate.

For spatial transcriptomics pathway activity analysis, gene sets for Gene Ontology^84^ terms and REACTOME^85^ pathways were downloaded from MsigDB.^86^ For each gene set, we used R package ‘AUCell’ to compute an activity score in each ST spot in each slide. Briefly, we used the expression matrix to compute gene expression rankings within each spot using “AUCell_buildRankings” function. For each pathway, area under the curve (AUC) was then calculated for each spot using “AUCell_caclAUC” function based on the ranked gene expression profiles, where AUC value then represents the fraction of genes within the top-ranking genes for each spot that are defined as part of the pathway gene set. In order to prioritize spatial region and condition specific pathways, we fit generalized linear models to test condition or spatial region cluster dependence of the pathway AUC values. As AUC values are significantly dependent on gene spot gene detection rate, we further including gene detection rate as a blocking co-variate in the model. Resultant p-values were further adjusted for multiple testing using Benjamini-Hochberg multiple testing correction. Pathway activity scores for single cell data were also computed and tested this way.

Spatial spot cluster adjacency networks for crypt top regions were computed as follows. For each slide, we subset crypt top region spots (as described above, crypt axis score > 1, and confirmed by visual examination where possible). For each crypt top spot in each slide, we computed Euclidean distances to each other spot using the spot coordinates of ST images. Using distances, for any given spot, we selected immediately spatially adjacent spots (dist < 4 and dist > 0 with respect to the downscaled, low resolution image coordinates) and counted the fraction of the directly adjacent spots occupied by different crypt top region clusters. We then computed the mean surrounding spot cluster fractions for all “central” spots in each cluster and used these values as an edge weight to construct a weighted, directed network for visualizing cluster spatial adjacency in UC, CPI-Colitis and HC slides. Diagonals (representing spots from the same transcriptome driven cluster occupying adjacent spots in tissue space) were kept. As before, R package ‘igraph’ was used to construct the network, ‘ggpraph’ was used to visualize the network. Networks were laid out for visualization using a force-directed Fruchterman-Reingold layout. The range of edge weights/widths and node sizes was standardized between visualizations of CPI-Colitis, UC and HC conditions.

Marker gene and differential gene expression analyses between conditions in both ST and scRNA-seq data were performed by using negative binomial generalized linear model statistical testing. In each case, confounding sources of variation stemming from spot/cell gene detection rate and batch/donor/slide effects were included in the model formula as blocking covariates. Benjamini-Hochberg multiple testing correction was used to estimate FDR. Genes with FDR < 5% were considered significantly differentially expressed. Additionally, we used R package MAST^87^ to identify differentially expressed genes which behave more in an on-off manner by testing the continuous and discrete components of the Hurdle model.

We also performed pseudobulk differential expression analysis for scRNA-seq datasets, computing sample pseudobulk values from raw UMI counts as described above. R package DESeq2^71^ was used to normalize pseudobulk counts and carry out differential expression analysis, blocking for confounding variation was before. Wald test was used to compute p-values, which were further adjusted for multiple testing using Benjamini-Hochberg multiple testing correction. Log2 fold change values were additionally shrunk to account for data heteroskedasticity.

Gene Ontology and pathway enrichment analyses of both scRNA-seq and spatial transcriptomics derived results were performed using the clusterProfiler R package.^88^ The annotation Dbi R package org.Hs.eg.db was used to map gene identifiers. Cluster marker sets and differentially expressed genes were tested individually for overrepresentation, with all expressed/detected genes in each case used as a background control. Hypergeometric *P* values were adjusted in each case for multiple testing using Benjamini–Hochberg correction as before. The results were visualized using the R packages clusterProfiler and ggplot2.

#### Spatial transcriptomics data analysis – CosMx

Raw image data processing was carried out using Nanostring CosMx pipeline. Cell segmentation polygon coordinates, transcript coordinates, cell meta data and cell morphology images were imported into R for further analysis. Cells with low molecule detection counts were filtered out from subsequent analysis; we further filtered out fields of view where more than 25% of all cells did not pass this quality threshold, as this would negatively impact cell adjacency-based analyses. Data from non-negative control probes in remaining cells was normalized using SCTransform as implemented in Seurat package and PCA dimensionality reduction was carried out. Initial clustering analysis highlighted some batch effects were present between different slides and fields of view, therefore harmony algorithm was used to batch correct reduced dimension components as described above. Harmonized reduced dimension components were then used as input for Louvain clustering (resolution .7) and cluster visualization using UMAP embeddings.

As in 10x data, all major cell lineages (epithelium, stroma, lymphocytes, myeloid) were further sub clustered for a higher resolution population analysis. In cases where sub-cluster analyses identified cell segmentation “doublets”, these were removed from sub-cluster annotations and only unambiguous clusters were kept. For distance-based gene expression analyses where cell identity is not directly relevant, these doublet cells were kept to avoid gaps in local tissue area. Doublet cells were identified as cells with mixed lineage marker cell transcriptomic signatures, and were largely accounted by immune cells, likely due to the positioning of these cells as they migrate through the tissue.

Using 10x single cell data generated in this study as a reference, the data was first subset to retain only genes for which probes were present within our CosMx dataset, and labels of broad cell populations were transferred using Seurat label transfer workflow to annotate closest cell populations between the two datasets. These were checked against canonical marker gene expression. In the case of granulocytes such as neutrophils, which were not captured in our 10x reference dataset due to technical difficulties in profiling these cells using single cell sequencing approaches, clusters were annotated manually using expression of canonical marker genes. While we were able to detect most major cell type populations in the dataset, due to the limitations of the probe set and/or in some cases non-specific probe signal, some cell populations could not be confidently differentiated. The distribution of all negative probe signal was examined and while negative signal was detected in ∼ 28% of all segmented cells, few cells had more than one false positive signal. As such, for marker gene expression and cell annotation purposes, we only counted cells as positive for a marker if more than one molecule per cell was detected, as it was extremely unlikely that the signal from both molecules would arise as a false positive. However, in some cases of key marker genes of rare, low expression transcripts (e.g. FOXP3/Tregs), the detection rate was overall very low and therefore non-specific. In these cases, cell clusters were named based on marker gene expression. These populations included: MAF^+^ T cells, which encompassed Tfh, Tph and Treg subpopulations; GNLY^+^ T cells – intra-epithelial/gamma-delta and “exhausted” HAVCR2^+^ T cells; Undiff – epithelial stem cells and secretory and absorptive progenitor cells.

Spatial niches were defined based on local cell type composition. Following cluster annotation, for each cell the identity of its immediately adjacent neighbors was counted, excluding itself. This resulted in a cell by cell type of adjacent neighbors count matrix, which was used as input for dimensionality reduction and clustering analysis. Clusters were annotated based on cell type composition or corresponding anatomical structures.

Receptor-ligand analysis was carried out as described in Visium analysis section above, except only receptor-ligand pairs present in the CosMx panel were used for the analysis and for each cell, surrounding cells rather than spots were used.

To detect CPI-bound cells, CosMx morphology and adjacent IF images were analyzed in QuPath. Morphology images and adjacent slices were manually pre-aligned using QuPath interactive image alignment, and then the affine transformation was estimated for each set of images that enabled the transfer of point coordinates between IF and CosMx data/images. To detect CPI-bound cells, we trained two image classifiers – one using CosMx morphology images and another to process IF images. In each case, example training cells were manually selected from several training images from different patients as belonging to either a) CD3^+^ cells (DAPI/CD3), b) CPI-Bound T cells (DAPI/CD3/IgG4), c) IgG4 cells/background IgG (IgG4 only) d) Other cells (DAPI) and a QuPath pixel classifier was trained. For each image, tissue area was manually selected and pixels were classified, fragments and holes in the resulting objects were removed and polygon shapes were simplified. To quantify CPI-binding levels, total area of CPI-bound and CPI-free was calculated, and adjusted for the total area of DAPI^+^ cells. For the analysis of CPI-bound cell localization with respect to CosMx data, polygon coordinates of CPI-bound and CPI-free T cells were first affine-transformed to the CosMx coordinate space. Individual cells detected by CosMx analysis at these coordinates were then classified as either: CPI-free, CPI-bound, near CPI-bound cell in z-coordinate space in one or more adjacent slices, or overlapping multiple CPI-bound cells.

#### TCR analysis

Single-cell TCR clonotypes were assembled using Cellranger VDJ software. Single-cell barcodes were then used to link corresponding VDJ clonotypes and gene expression data. Droplets where multiple clonotypes or chains were reconstructed were further filtered out as doublets/multiplets, with the exception of T cells bearing two alpha and one beta chains, which can occur naturally at a rare frequency during VDJ recombination.

VDJTools^89^ software was used to compute TCR repertoire statistics for individual samples, as well as cluster-level statistics. Assembled TCR CDR3β amino acid sequences were used to query VDJdb^90^ (vdjdb-2021-02-02), McPAS^91^ (downloaded 2021-07-21) and TBAdb(downloaded 2021-07-21) for overlap with known and/or public TCR sequences. GLIPH2^92^ was used to predict antigen specificity of TCR clonotypes and analyzed specificity clusters with Fisher’s p-values ≤ 0.05.

T cell clonality was defined by computing Shannon Entropy as well as computing the fraction of total clonal space occupied by any given clonotype in a sample. For grouping cells into expanded/non-expanded binary categories, a clonotype was considered expanded if more than one clone was detected within the same sample and sample type (blood and biopsy samples from the same donor were considered separately and addressed by trafficking analyses).

To examine overlaps of TCR repertoires between populations and assess phenotypic conversion and trafficking, we computed Morisita's index using R divo package between all pairwise blood and tissue T cell clusters. For shared clonal network analysis and visualization between and within blood and tissue T cell populations, CD4^+^ and CD8^+^ cell populations were considered separately to aid clarity, as little clonal sharing was observed between the two lineages as expected. As before, R package igraph was used to construct weighted, undirected CD4^+^ and CD8^+^ cell networks, with edge weights defined as Morisita’s clone overlap index. For CD8^+^ cell networks, for clarity we further filtered out edges with very low clonal overlap. We defined these filter thresholds based on the distribution of Morisita’s index between all CD4 and CD8 cell populations, as these are likely to be spurious connections/noise, considering all edges below the 95^th^ percentile as low confidence. As CD4^+^ cell populations are a lot less clonal than CD8^+^, all edges were retained for visualization. However, much larger sampling of CD4^+^ cells of each individual would be required to confidently infer clonal dynamics of CD4^+^ populations, as most of the TCR sharing between populations was observed as only one or two clones, which may be spurious in the cases of closely phenotypically related cell clusters (but not blood-tissue sharing) due to sources of errors in the dataset such as barcode multiplets.^93^ Raw, unfiltered counts of all overlaps in CD4 and CD8 populations in CPI-Colitis, UC and HC were visualized as heatmaps. Networks were visualized using R package ggraph. Graphs were laid out for visualization using a force-directed Fruchterman-Reingold layout. As before, edge width ranges were standardized between conditions for comparable visualization.

Hierarchical clustering of CD4 and CD8 T cell populations was similarly carried out with pairwise Morisita’s overlap index matrix used to define similarity between populations in CPI-Colitis, HC and UC samples. Clustering was carried out in R using hclust() function and a complete linkage method.

#### Computational identification of Nivolumab-bound T cells

To train a model for prediction of PD-1 protein expression, we first split the CD3^+^ CITE-Seq dataset into checkpoint-inhibitor treated and non-treated sample cells. In the latter subset, we set aside cells from one UC donor as an independent control set as well as sampling 33% of other donor cells as a random hold-out testing subset. The other non-checkpoint treated cells constituted the training data.

For each cell, the following variables were put forward for feature selection: the first 30 harmony-integrated reduced dimension components, cell cycle scores and phase classifications, 10X reactions (all reactions had a mix of checkpoint treated and non-treated samples hashed together), total cell RNA counts, gene detection rate per cell, mitochondrial and ribosomal gene percentages and expression of PDCD1 mRNA. Following recursive feature selection fitting a random forest regression model for prediction of PD-1 protein expression, we ranked features by their relative importance and selected those with importance > 5 for final model training.

Using selected features, we then fit a quantile regression random forest model,^14^ as implemented in R package quantregForest (version 1.3-7), using the training data set. We then predicted the conditional quantile distribution of PD-1 protein expression in both 33% hold out and independent donor validation datasets. We compared the predicted/expected values (at 0.5 quantile) with measured PD-1 expression by CITE-Seq antibodies to assess the accuracy of the model, obtaining strong correlation between measured and predicted values in both validation datasets.

We then predicted the expected PD-1 expression in checkpoint-inhibitor treated sample cells. For each cell, we compared the measured PD-1 expression with predicted PD-1 expression at .99, 0.95, 0.9 and 0.85 quantile thresholds from the model to identify T cells where measured PD-1 expression differs from predicted PD-1 expression at 1%, 5%, 10% and 15% confidence intervals, identifying these cells as those still putatively bound to Nivolumab.

### Quantification and statistical analysis

Data were analyzed in Prism version 10.0.3 or R version 4.3.0 and results associated with p < 0.05 were considered statistically significant. Statistical significance was analyses by 2-tailed Student’s t-test, Wilcox rank sum test, negative binomial and likelihood ratio tests, as indicated. Error bars show standard error of the mean, as indicated. Sample size used in each case was estimated based on assay sensitivity and expected heterogeneity of samples, previous similar studies and pilot experiment outcomes.

### Additional resources

All analyzed scRNA-seq and ST data has been made available via an interactive data portal at https://simmonslab.shinyapps.io/CPI_COLITIS_DATA_PORTAL/.

Additional data associated with the study has been made available at Mendeley Data: https://doi.org/10.17632/7z8yx644hb.1.

## Supplemental information

Document S1. Figures S1–S10

Table S1. Patient sample cohort characteristics overview, related to Figure 1 and STAR Methods

Table S2. scRNA-Seq cell type cluster annotations, related to Figures 1, 2, and 6, and STAR Methods

Table S3. Data from gene-expression based analyses from scRNA-Seq and ST datasets, related to Figures 1, 2, 5, and 6, and STAR Methods

Document S2. Article plus supplemental information
