# Supplementary material for: Tracking in situ checkpoint inhibitor-bound target T cells in patients with checkpoint-induced colitis
Source: Cancer Cell. 2024 May 13;42(5):797–814.e15. doi: 10.1016/j.ccell.2024.04.010 (PMC12979251; doi:10.1016/j.ccell.2024.04.010)
Supplement: Document S1. Figures S1–S10 [file mmc1.pdf]

Cancer Cell, Volume *42*

## Supplemental information

# Tracking *in situ* checkpoint inhibitor-bound target T cells in patients with checkpoint-induced colitis

**Tarun Gupta, Agne Antanaviciute, Chloe Hyun-Jung Lee, Rosana Ottakandathil Babu, Anna Aulicino, Zoe Christoforidou, Paulina Siejka-Zielinska, Caitlin O'Brien-Ball, Hannah Chen, David Fawkner-Corbett, Ana Sousa Geros, Esther Bridges, Colleen McGregor, Nicole Cianci, Eve Fryer, Nasullah Khalid Alham, Marta Jagielowicz, Ana Mafalda Santos, Martin Fellermeyer, Simon J. Davis, Kaushal Parikh, Vincent Cheung, Lulia Al-Hillawi, Sarah Sasson, Stephanie Slevin, Oliver Brain, TIP Consortium, Ricardo A. Fernandes, Hashem Koohy, and Alison Simmons**

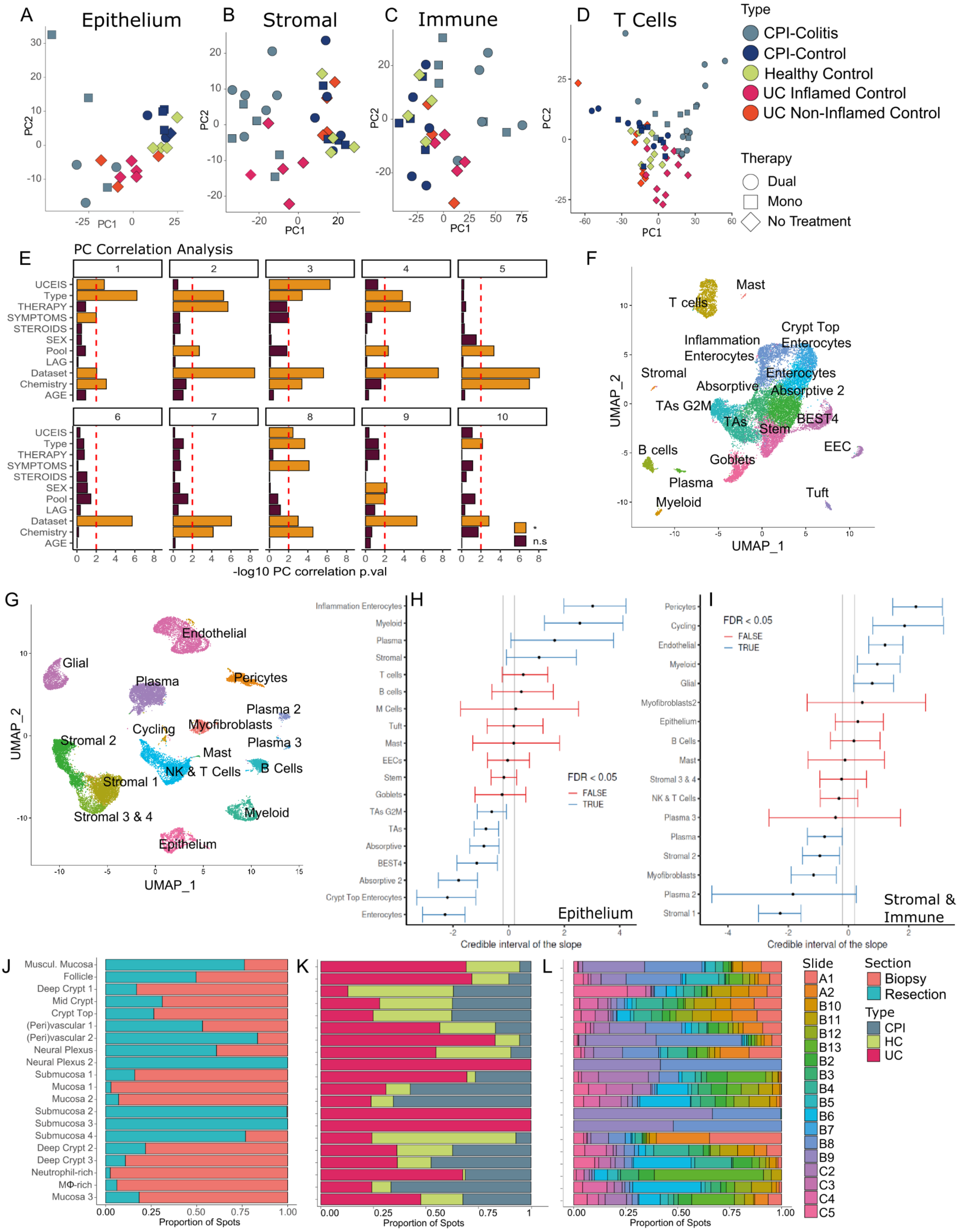

A
Epithelium
B
Stromal
C
Immune
D
T Cells
PC1
PC2
Type
CPI-Colitis
CPI-Control
Healthy Control
UC Inflamed Control
UC Non-Inflamed Control
Therapy
Dual
Mono
No Treatment
E
PC Correlation Analysis
UCEIS
Type
THERAPY
SYMPTOMS
STEROIDS
SEX
Pool
LAG
Dataset
Chemistry
AGE
*
n.s
-log10 PC correlation p.val
F
Mast
T cells
Crypt Top Enterocytes
Inflammation Enterocytes
Stromal
Enterocytes
Absorptive
Absorptive 2
TAs G2M
TAs
Stem
BEST4
B cells
EEC
Goblets
Plasma
Tuft
Myeloid
UMAP_1
UMAP_2
G
Endothelial
Glial
Plasma
Pericytes
Plasma 2
Cycling
Myofibroblasts
Stromal 2
Plasma 3
Mast
NK & T Cells
B Cells
Stromal 1
Stromal 3 & 4
Myeloid
Epithelium
UMAP_1
UMAP_2
H
Inflammation Enterocytes
Myeloid
Plasma
Stromal
T cells
B cells
M Cells
Tuft
Mast
EECs
Stem
Goblets
TAs G2M
TAs
Absorptive
BEST4
Absorptive 2
Crypt Top Enterocytes
Enterocytes
FDR < 0.05
FALSE
TRUE
Epithelium
Credible interval of the slope
I
Pericytes
Cycling
Endothelial
Myeloid
Glial
Myofibroblasts2
Epithelium
B Cells
Mast
Stromal 3 & 4
NK & T Cells
Plasma 3
Plasma
Stromal 2
Myofibroblasts
Plasma 2
Stromal 1
FDR < 0.05
FALSE
TRUE
Stromal & Immune
Credible interval of the slope
J
Muscul. Mucosa
Follicle
Deep Crypt 1
Mid Crypt
Crypt Top
(Peri)vascular 1
(Peri)vascular 2
Neural Plexus
Neural Plexus 2
Submucosa 1
Mucosa 1
Mucosa 2
Submucosa 2
Submucosa 3
Submucosa 4
Deep Crypt 2
Deep Crypt 3
Neutrophil-rich
MΦ-rich
Mucosa 3
Proportion of Spots
K
Proportion of Spots
L
Proportion of Spots
Slide
A1
A2
B10
B11
B12
B13
B2
B3
B4
B5
B6
B7
B8
B9
C2
C3
C4
C5
Section
Biopsy
Resection
Type
CPI
HC
UC

## Supplementary Figure S1. scRNA-Seq and ST Atlas in CPI-Colitis and UC, Related to Figure 1.

**A-D.** Principal component analysis plot of per sample, normalized scRNA-seq pseudo-bulk using the top 1000 most highly variable genes. The first two principal components are shown. Epithelial (**A**), stromal (**B**) and immune (**C**) compartments, as well as T cells (**D**), from colonic tissue sample cells were analyzed and shown separately.

**E.** Bar plot visualizing clinical and technical dataset feature correlation with the first ten principal components in T cell pseudobulk PCA analysis, visualized in **D**. Significant correlations are shown in orange. Kruskal-Wallis rank sum test and Spearman's correlation were used to test for significance.

**F.** UMAP embedding visualizes scRNA-seq clusters of cell populations obtained from epithelial crypt dissociation strategy.

**G.** UMAP embedding visualizes scRNA-seq clusters of cell populations obtained from stromal/$CD45^+$ isolation strategy.

**H.** Abundance analysis of single cell population epithelial clusters, comparing CPI-colitis versus non-inflamed control sample cells. Over-represented cell subpopulations in CPI-colitis are indicated by positive slope interval, while under-represented cell subpopulations are negative. Information on individual cell clusters is summarized in **Tables S2** and **S3**.

**I.** Abundance analysis of single cell population of broad stromal and immune cell clusters, comparing CPI-colitis versus non-inflamed control sample cells. Over-represented cell subpopulations in CPI-colitis are indicated by positive slope interval, while under-represented cell subpopulations are negative. Information on individual cell clusters is summarized in **Tables S2** and **S3**.

**J.** ST integrated spot cluster region composition bar plot by section cut type. Populations are abbreviated as follows: MΦ-rich – Macrophage rich.

**K.** ST integrated spot cluster region composition bar plot by sample type. Populations are abbreviated as follows: MΦ-rich – Macrophage rich.

**L.** ST integrated spot cluster region composition bar plot by individual section. Populations are abbreviated as follows: MΦ-rich – Macrophage rich.

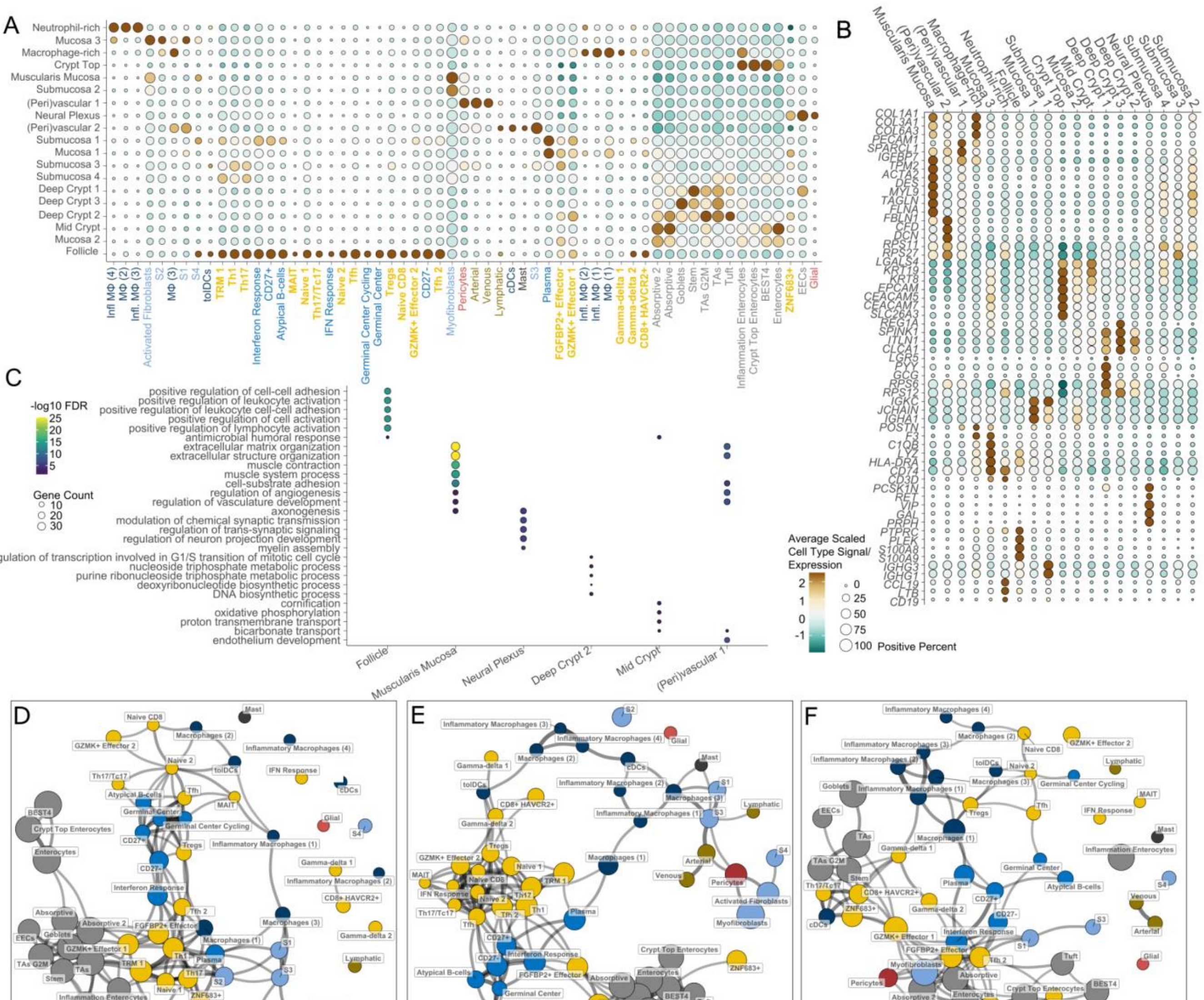
A
Neutrophil-rich
Mucosa 3
Macrophage-rich
Crypt Top
Muscularis Mucosa
Submucosa 2
(Peri)vascular 1
Neural Plexus
(Peri)vascular 2
Submucosa 1
Mucosa 1
Submucosa 3
Submucosa 4
Deep Crypt 1
Deep Crypt 3
Deep Crypt 2
Mid Crypt
Mucosa 2
Follicle
Infl MΦ (4)
MΦ (2)
Infl. MΦ (3)
Activated Fibroblasts
S2
MΦ (3)
S1
S4
tolDCs
TRM 1
Th1
Th17
Interferon Response
CD27+
Atypical B-cells
MAIT
Naive 1
Th17/Tc17
IFN Response
Naive 2
Tfh
Germinal Center Cycling
Germinal Center
Tregs
Naive CD8
GZMK+ Effector 2
CD27-
Tfh 2
Myofibroblasts
Pericytes
Arterial
Venous
Lymphatic
cDCs
Mast
S3
Plasma
FGFBP2+ Effector
GZMK+ Effector 1
Infl. MΦ (2)
Infl. MΦ (1)
MΦ (1)
Gamma-delta 1
Gamma-delta 2
CD8+ HAVCR2+
Absorptive 2
Absorptive
Goblets
Stem
TAs G2M
TAs
Tuft
Inflammation Enterocytes
Crypt Top Enterocytes
BEST4
Enterocytes
ZNF683+
EECs
Glial
B
Muscularis Mucosa
(Peri)vascular 2
Macrophage-rich
(Peri)vascular 1
Mucosa 3
Neutrophil-rich
Follicle
Submucosa 1
Mucosa 1
Crypt Top
Mucosa 2
Mid Crypt
Deep Crypt 2
Deep Crypt 1
Deep Crypt 3
Neural Plexus
Submucosa 4
Submucosa 3
Submucosa 2
COL1A1
COL3A1
COL6A3
PECAM1
SPARC
IGFBP7
TPM2
ACTA2
DES
MYL9
TAGLN
FLNA
FBLN1
CFD
PI16
RPS11
RPS27
LGALS4
KRT19
KRT8
EPCAM
CEACAM5
CEACAM7
SLC26A3
REG1A
SPINK1
ITLN1
CLCA1
LGR5
PYY
GCG
RPS6
RPS12
IGKC
JCHAIN
IGHA1
POSTN
F3
C1QB
LYZ
HLA-DRA
CD74
CD3D
PCSK1N
RET
VIP
GAL
PRPH
PTPRC
PLEK
S100A8
S100A9
IGHG3
IGHG1
CCL19
LTB
CD19
Average Scaled
Cell Type Signal/
Expression
2
1
0
-1
0
25
50
75
100
Positive Percent
C
-log10 FDR
25
20
15
10
5
Gene Count
10
20
30
positive regulation of cell-cell adhesion
positive regulation of leukocyte activation
positive regulation of leukocyte cell-cell adhesion
positive regulation of cell activation
positive regulation of lymphocyte activation
antimicrobial humoral response
extracellular matrix organization
extracellular structure organization
muscle contraction
muscle system process
cell-substrate adhesion
regulation of angiogenesis
regulation of vasculature development
axonogenesis
modulation of chemical synaptic transmission
regulation of trans-synaptic signaling
regulation of neuron projection development
myelin assembly
regulation of transcription involved in G1/S transition of mitotic cell cycle
nucleoside triphosphate metabolic process
purine ribonucleoside triphosphate metabolic process
deoxyribonucleotide biosynthetic process
DNA biosynthetic process
cornification
oxidative phosphorylation
proton transmembrane transport
bicarbonate transport
endothelium development
Follicle
Muscularis Mucosa
Neural Plexus
Deep Crypt 2
Mid Crypt
(Peri)vascular 1
D
HC
E
UC
F
CPI
Cell Type
B cells
Endothelium
Epithelium
Fibroblasts
Glial
Mast
Myeloid
Pericytes
T cells
% Spots Positive
1
5
10
25
50
Weight
0.00
0.25
0.50
0.75
1.00
G
Stem Cells
Crypt Top Enterocytes
Crypt.Top.Enterocytes
2.94
Myofibroblasts
1.18
2.35
S2
0.30
Enterocytes
0.02
1.04
H
CD27+ B Cells
GC B Cells
Tfh Cells
Naive T Cells
Cell type signal
CD3D
MS4A1
Exp.

## Supplementary Figure S2. Unbiased Visium ST analysis of CPI-Colitis and UC. Related to Figure 1.

**A.** Dot plot heatmap shows cell type enrichment and distribution across all spatial regions identified in **Figure 1C**.

**B.** Dot plot heatmap shows cluster marker gene expression distribution across all spatial regions identified in **Figure 1C**.

**C.** Dot plot visualizing top gene ontology (GO) terms enriched in selected ST region marker genes.

**D-F.** Single cell population type pairwise signal spatial correlation network visualizes cell populations signal most strongly co-occurs within the same spatial locations. Edge width and intensity represent correlation between populations. Edges below $r < 0.15$ are not shown for clarity. Nodes represent cell types, colored by broad compartment. Node size represents the percentage of all spots with cell type signal > 0. Spots from healthy control colon slides are shown in **D**, UC in **E** and CPI-colitis in **F**. Exp=Expression.

**G.** Epithelial stem cell, crypt top enterocyte, myofibroblast, stromal 2/telocyte and enterocyte cell type signature signal spatial distribution visualized over a representative healthy control tissue section individually (left and middle) and blended using cell2location package (right), with reference H&E image shown in **Figure 1D.** Scale=1mm

**H.** Spatial distribution of selected follicular immune single cell type signatures (left) and individual canonical T and B cell (*CD3D*, *MS4A1*/CD20) gene expression (right) in a representative UC ST slide. Zoomed in region around lymphoid tissue follicles are indicated by a dashed line in H&E image (middle). Color bars represent cell type spot composition probability scores (left) and normalized gene expression values (right). Scale=1mm.

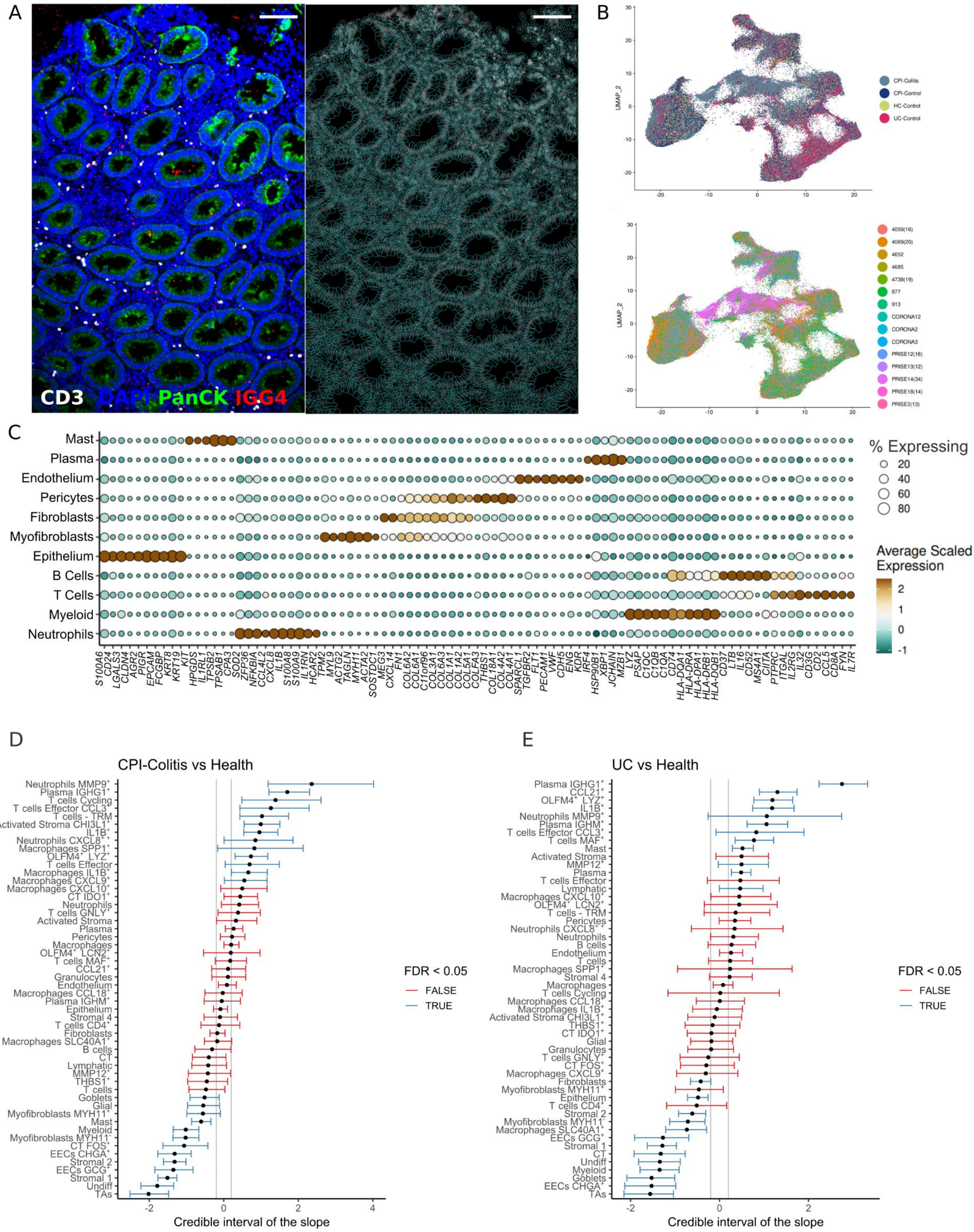
A
CD3 DAPI PanCK IGG4
B
UMAP_2
CPI-Colitis
CPI-Control
HC-Control
UC-Control
4059(16)
4069(20)
4652
4685
4738(19)
877
913
CORONA12
CORONA2
CORONA3
PRISE12(16)
PRISE13(12)
PRISE14(34)
PRISE18(14)
PRISE3(13)
C
Mast
Plasma
Endothelium
Pericytes
Fibroblasts
Myofibroblasts
Epithelium
B Cells
T Cells
Myeloid
Neutrophils
S100A6 CD24 LGALS3 CLDN4 AGR2 PIGR EPCAM FCGBP KRT8 KRT19 KIT HPGDS IL1RL1 TPSB2 TPSAB1 CPA3 SOD2 ZFP36 NFKBIA CCL4L2 CXCL8 IL1B S100A8 S100A9 IL1RN HCAR2 TPM2 MYL9 ACTG2 TAGLN MYH11 ACTA2 SOSTDC1 MEG3 CXCL14 FN1 COL6A2 COL6A1 C11orf96 COL3A1 COL6A3 COL1A1 COL1A2 COL5A1 COLFA3 THBS1 COL18A1 COL4A2 COL4A1 SPARCL1 TGFBR2 FLT1 PECAM1 VWF CDH5 ENG KDR IRF4 HSP90B1 XBP1 JCHAIN MZB1 LYZ PSAP C1QC C1QB C1QA CD74 HLA-DQA1 HLA-DRA HLA-DPA1 HLA-DRB1 HLA-DQB1 CD37 LTB IL16 CD52 MS4A1 CIITA PTPRC ITGAL IL2RG IL32 CD3G CD2 CCL5 CD8A FYN IL7R
% Expressing
20
40
60
80
Average Scaled Expression
2
1
0
-1
D
CPI-Colitis vs Health
Neutrophils MMP9+
Plasma IGHG1+
T cells Cycling
T cells Effector CCL3+
T cells - TRM
Activated Stroma CHI3L1+
IL1B+
Neutrophils CXCL8+
Macrophages SPP1+
OLFM4+ LYZ+
T cells Effector
Macrophages IL1B+
Macrophages CXCL9+
Macrophages CXCL10+
CT IDO1+
Neutrophils
T cells GNLY+
Activated Stroma
Plasma
Pericytes
Macrophages
OLFM4+ LCN2+
T cells MAF+
CCL21+
Granulocytes
Endothelium
Macrophages CCL18+
Plasma IGHM+
Epithelium
Stromal 4
T cells CD4+
Fibroblasts
Macrophages SLC40A1+
B cells
CT
Lymphatic
MMP12+
THBS1+
T cells
Goblets
Glial
Myofibroblasts MYH11+
Mast
Myeloid
Myofibroblasts MYH11-
CT FOS+
EECs CHGA+
Stromal 2
EECs GCG+
Stromal 1
Undiff
TAs
-2
0
2
4
Credible interval of the slope
FDR < 0.05
FALSE
TRUE
E
UC vs Health
Plasma IGHG1+
CCL21+
OLFM4+ LYZ+
IL1B+
Neutrophils MMP9+
Plasma IGHM+
T cells Effector CCL3+
T cells MAF+
Mast
Activated Stroma
MMP12+
Plasma
T cells Effector
Lymphatic
Macrophages CXCL10+
OLFM4+ LCN2+
T cells - TRM
Pericytes
Neutrophils CXCL8+
Neutrophils
B cells
Endothelium
T cells
Macrophages SPP1+
Stromal 4
Macrophages
T cells Cycling
Macrophages CCL18+
Macrophages IL1B+
Activated Stroma CHI3L1+
THBS1+
CT IDO1+
Glial
Granulocytes
T cells GNLY+
CT FOS+
Macrophages CXCL9+
Fibroblasts
Myofibroblasts MYH11+
Epithelium
T cells CD4+
Stromal 2
Myofibroblasts MYH11-
Macrophages SLC40A1+
EECs GCG+
Stromal 1
CT
Undiff
Myeloid
Goblets
EECs CHGA+
TAs
-2
0
2
Credible interval of the slope
FDR < 0.05
FALSE
TRUE

## Supplementary Figure S3. Subcellular resolution CosMx ST analysis of CPI-Colitis and UC. Related to Figure 1.

**A.** Representative field of view image of a cellular morphology stain compositive image using CosMx subcellular resolution platform (left) and cell segmentation overlay (right). Scale=100µm

**B.** UMAP overlay showing sample type distribution (top) of segmented cells in transcriptome-based clustering analysis from sub-cellular CosMx ST dataset, with reference to clusters shown in **Figure 1D**. UMAP overlay with cells colored by donor of origin is shown at the bottom.

**C.** Dotplot heatmap shows top cluster marker genes for clustering analysis of CosMx ST dataset shown in **Figure 1D**.

**D-E.** Abundance analysis of all cell clusters detected in CosMx data clustering analyses, CPI-Colitis vs healthy control samples (D) and UC vs healthy control samples (E). Over-represented cell subpopulations in CPI-colitis are indicated by positive slope interval, while under-represented cell subpopulations are negative. Cell cluster markers and related information is summarized in **Table S3**.

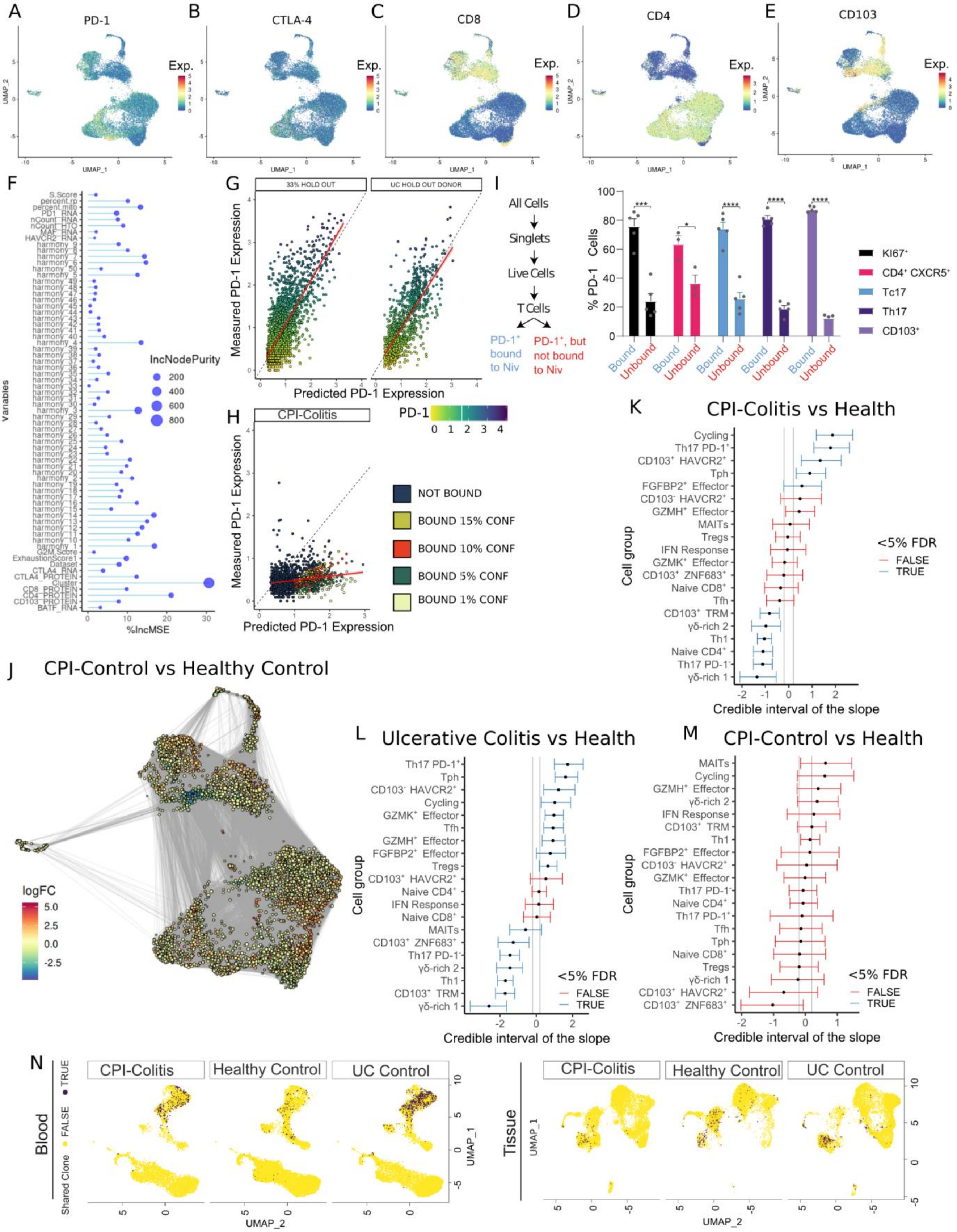
A
PD-1
B
CTLA-4
C
CD8
D
CD4
E
CD103
Exp.
UMAP_1
UMAP_2
F
S.Score
percent.rp
percent.mito
PD1_RNA
nCount_RNA
nCount_HTO
MAF_RNA
HAVCR2_RNA
G2M.Score
ExhaustionScore1
Dataset
CTLA4_RNA
CTLA4_PROTEIN
Cluster
CD8_PROTEIN
CD4_PROTEIN
CD103_PROTEIN
BATF_RNA
IncNodePurity
Variables
%IncMSE
G
33% HOLD OUT
UC HOLD OUT DONOR
Measured PD-1 Expression
Predicted PD-1 Expression
H
CPI-Colitis
PD-1
NOT BOUND
BOUND 15% CONF
BOUND 10% CONF
BOUND 5% CONF
BOUND 1% CONF
I
All Cells
Singlets
Live Cells
T Cells
PD-1+ bound to Niv
PD-1+, but not bound to Niv
% PD-1 Cells
Bound
Unbound
KI67+
CD4+ CXCR5+
Tc17
Th17
CD103+
J
CPI-Control vs Healthy Control
logFC
K
CPI-Colitis vs Health
L
Ulcerative Colitis vs Health
M
CPI-Control vs Health
Cell group
Credible interval of the slope
<5% FDR
FALSE
TRUE
Cycling
Th17 PD-1+
CD103+ HAVCR2+
Tph
FGFBP2+ Effector
CD103- HAVCR2+
GZMH+ Effector
MAITs
Tregs
IFN Response
GZMK+ Effector
CD103+ ZNF683+
Naive CD8+
Tfh
CD103+ TRM
γδ-rich 2
Th1
Naive CD4+
Th17 PD-1-
γδ-rich 1
N
Blood
Tissue
CPI-Colitis
Healthy Control
UC Control
Shared Clone
FALSE
TRUE
UMAP_1
UMAP_2

## Supplementary Figure S4. CITE-Seq and CPI-binding analysis of T cells in CPI-Colitis. Related to Figure 2 and Figure 3.

**A.** UMAP overlay visualizing expression of PD-1, as detected by CITE-Seq analysis. Reference UMAP shown in **Figure 2A.**

**B.** UMAP overlay visualizing expression of CTLA-4, as detected by CITE-Seq analysis. Reference UMAP shown in **Figure 2A.**

**C.** UMAP overlay visualizing expression of CD8, as detected by CITE-Seq analysis. Reference UMAP shown in **Figure 2A.**

**D.** UMAP overlay visualizing expression of CD4, as detected by CITE-Seq analysis. Reference UMAP shown in **Figure 2A.**

**E.** UMAP overlay visualizing expression of CD103, as detected by CITE-Seq analysis. Reference UMAP shown in **Figure 2A.**

**F.** Variable importance plot visualizing training features prior to feature selection for the CPI-binding detection model.

**G.** PD-1 expression regression model validation shows the correlation between predicted and measured PD-1 protein expression in non-immunotherapy 33% hold out cells not used for model training (left) and all cells from a single UC hold out donor not used for model training (right). Red line shows linear fit, dotted grey line shows x=y. Pearson's correlation (left) r=0.7612616, p-value < 2.2e-16 (right) r=0.7645451, p-value < 2.2e-16.

**H.** Scatterplot shows the predicted and measured PD-1 expression in CPI-treated sample cells. Cells are colored by the quantile of the divergence between the predicted and measured PD-1 values, with the highest confidence bound cells represented as 1% CONF. Red line shows linear fit, dotted grey line shows x=y, Pearson's correlation r=0.2088881, p-value < 2.2e-16.

**I.** Phenotypic FACS-based characterization of CPI-bound CPI-colitis patient samples, showing proportion of all PD-1$^+$KI67$^+$ (cycling), PD-1$^+$CD4$^+$CXCR5$^+$ (Tfh), PD-1$^+$CD8$^+$CXCR6$^+$ (Tc17), PD-1$^+$CD4$^+$CXCR6$^+$ (Th17) and PD-1$^+$CD103$^+$ (tissue resident) that remain bound to Nivolumab in colitis patient samples within 100 days of immunotherapy. Unpaired t test with Welch's correction. Bar plot (mean +/- standard error of mean), ns = not significant, * p value <0.05, *** p value = 0.0001, **** p value < 0.0001. Error bars represent mean +/- standard error of mean limits. n=3-5 samples.

**J.** UMAP overlay visualizing log fold changes of local neighborhood abundance differences when comparing CPI-control and healthy control patient samples. Each point represents a local neighborhood of cells within the single cell k-nearest neighbor graph visualized as a UMAP embedding. Regions on the embedding with highest cellular enrichment in CPI-control samples are shown in red, while depleted regions are in blue.

**K.** Abundance analysis of T cell subclusters, comparing CPI-colitis vs health. Over-represented cell subpopulations in each case are indicated by positive slope interval, while under-represented cell subpopulations are negative.

**L.** Abundance analysis of T cell subclusters, comparing UC vs health. Over-represented cell subpopulations in each case are indicated by positive slope interval, while under-represented cell subpopulations are negative.

**M.** Abundance analysis of T cell subclusters, comparing CPI-control vs health. Over-represented cell subpopulations in each case are indicated by positive slope interval, while under-represented cell subpopulations are negative.

**N.** UMAP overlay visualizing the cluster distribution of TCR clones in PBMC samples shared with colonic tissue biopsies (left) and TCR clones in colonic tissue biopsies shared with paired PBMC samples (right).

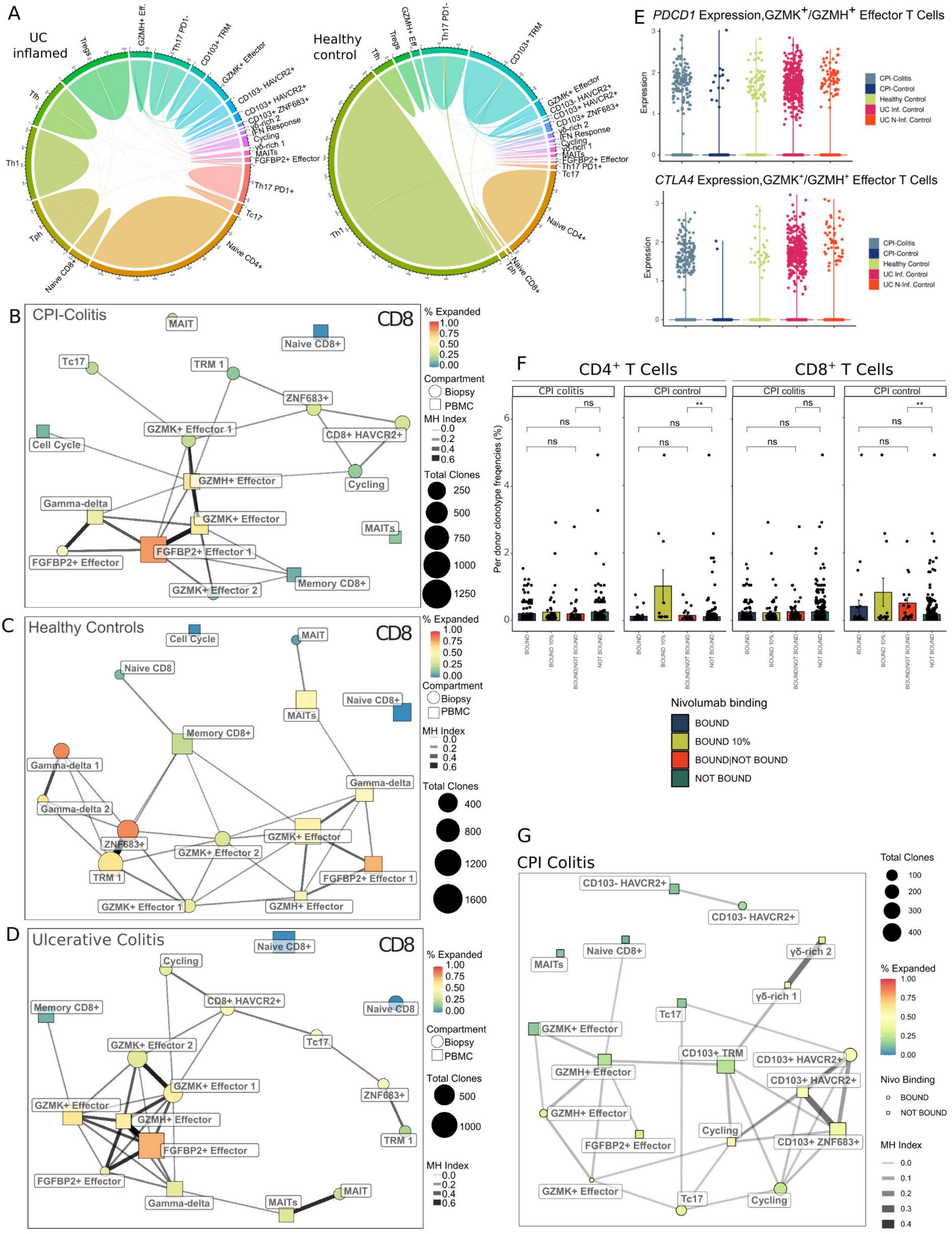

A
UC inflamed
Healthy control
B CPI-Colitis CD8
C Healthy Controls CD8
D Ulcerative Colitis CD8
% Expanded
Compartment
Biopsy
PBMC
MH Index
Total Clones
E PDCD1 Expression,GZMK+/GZMH+ Effector T Cells
CTLA4 Expression,GZMK+/GZMH+ Effector T Cells
Expression
CPI-Colitis
CPI-Control
Healthy Control
UC Inf. Control
UC N-Inf. Control
F CD4+ T Cells
CD8+ T Cells
CPI colitis
CPI control
Per donor clonotype frequencies (%)
Nivolumab binding
BOUND
BOUND 10%
BOUND|NOT BOUND
NOT BOUND
G CPI Colitis
Nivo Binding

## Supplementary Figure S5. T cell VDJ-based clonality analysis. Related to Figure 3.

**A.** Circos plots visualizing TCR clonal sharing and expansion levels between colonic tissue T cell populations in samples from Ulcerative Colitis (left), and healthy control samples (right).

**B.** Network plot visualizing TCR clonal trafficking between PBMC and colonic tissue T cell populations in samples from $CD8^+$ T cells in CPI-colitis. Each node represents a T cell population as defined by scRNA-seq transcriptome phenotype while edges represent clonal sharing as defined by Morisita's Index.

**C.** Network plot visualizing TCR clonal trafficking between PBMC and colonic tissue T cell populations in samples from $CD8^+$ T cells in healthy control samples. Each node represents a T cell population as defined by scRNA-seq transcriptome phenotype while edges represent clonal sharing as defined by Morisita's Index.

**D.** Network plot visualizing TCR clonal trafficking between PBMC and colonic tissue T cell populations in samples from $CD8^+$ T cells in UC. Each node represents a T cell population as defined by scRNA-seq transcriptome phenotype while edges represent clonal sharing as defined by Morisita's Index.

**E.** Violin plot visualizing differential expression of *PDCD1* and *CTLA4* mRNAs in inflammation in colonic cytotoxic $CD8^+$ T cell clusters. Centre bar indicates median value. *PDCD1*: CPI-Colitis vs Health – p.val = 0.004581825; CPI-Colitis vs UC – p.val = 0.1747312. *CTLA4*: CPI-Colitis vs Health – p.val= 0.0006036874, CPI-Colitis vs UC – p.val= 0.2090165. Negative binomial test.

**F.** Bar plot (mean, +/- standard error of mean) visualizing clonal expansions in CPI-bound cells identified with 0.05 confidence threshold (BOUND), < 0.1 confidence threshold (BOUND 10%), clones wherein some cells are CPI-bound and some CPI-free (BOUND|NOT BOUND) and clones wherein all cells are predicted to be CPI-free (NOT BOUND). Wilcox rank test. ns – not significant, ** p.value < 0.01.

**G.** Network plot visualizing TCR clonal sharing between CPI-bound and CPI-free cells and their phenotype in CPI colitis. Each node represents a T cell population as defined by scRNA-Seq transcriptome phenotype while edges represent clonal sharing as defined by Morisita's Index. CPI-bound cells are denoted as circles, CPI-free as squares.

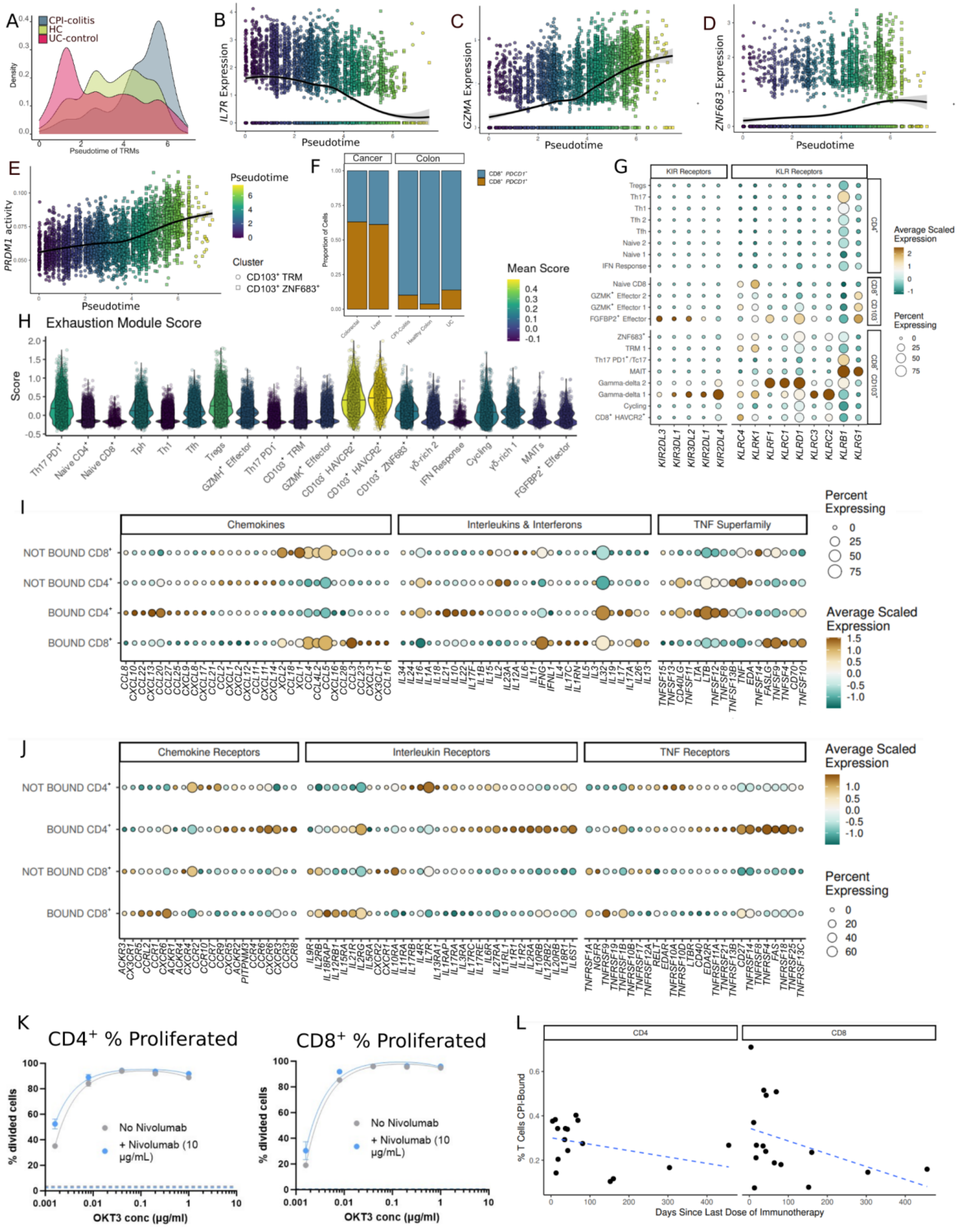

A
CPI-colitis
HC
UC-control
Density
Pseudotime of TRMs
B
IL7R Expression
Pseudotime
C
GZMA Expression
Pseudotime
D
ZNF683 Expression
Pseudotime
E
PRDM1 activity
Pseudotime
Pseudotime
Cluster
CD103+ TRM
CD103+ ZNF683+
F
Cancer
Colon
Proportion of Cells
CD8+ PDCD1-
CD8+ PDCD1+
Colorectal
Liver
CPI-Colitis
Healthy Colon
UC
Mean Score
G
KIR Receptors
KLR Receptors
Tregs
Th17
Th1
Tfh 2
Tfh
Naive 2
Naive 1
IFN Response
Naive CD8
GZMK+ Effector 2
GZMK+ Effector 1
FGFBP2+ Effector
ZNF683+
TRM 1
Th17 PD1+/Tc17
MAIT
Gamma-delta 2
Gamma-delta 1
Cycling
CD8+ HAVCR2+
CD4+
CD8+ CD103-
CD8+ CD103+
KIR2DL3
KIR3DL1
KIR3DL2
KIR2DL1
KIR2DL4
KLRC4
KLRK1
KLRF1
KLRC1
KLRD1
KLRC3
KLRC2
KLRB1
KLRG1
Average Scaled Expression
Percent Expressing
H
Exhaustion Module Score
Score
Th17 PD1+
Naive CD4+
Naive CD8+
Tph
Th1
Tfh
Tregs
GZMH+ Effector
Th17 PD1-
CD103+ TRM
GZMK+ Effector
CD103- HAVCR2+
CD103+ HAVCR2+
CD103+ ZNF683+
γδ-rich 2
IFN Response
Cycling
γδ-rich 1
MAITs
FGFBP2+ Effector
I
Chemokines
Interleukins & Interferons
TNF Superfamily
NOT BOUND CD8+
NOT BOUND CD4+
BOUND CD4+
BOUND CD8+
Percent Expressing
Average Scaled Expression
CCL8 CXCL10 CCL22 CXCL13 CCL20 CCL27 CCL25 CXCL9 CCL8 CXCL17 CCL21 CCL2 CXCL1 CXCL2 CCL12 CCL11 CCL14 CXCL14 CCL18 XCL2 XCL1 CCL4 CCL4L2 CCL5 CXCL16 CCL28 CCL3 CCL23 CX3CL1 CCL16
IL34 IL24 IL16 IL1A IL18 IL21 IL10 IL22 IL17F IL15 IL2 IL23A IL12A IL6 IL11 IFNG IFNL1 IL4 IL17C IL1RN IL5 IL3 IL32 IL19 IL17 IL17A IL26 IL13
TNFSF15 TNFSF13 CD40LG TNFSF11 LTA LTB TNFSF12 TNFSF8 TNFSF13B TNF EDA TNFSF14 FASLG TNFSF9 TNFSF4 CD70 TNFSF10
J
Chemokine Receptors
Interleukin Receptors
TNF Receptors
NOT BOUND CD4+
BOUND CD4+
NOT BOUND CD8+
BOUND CD8+
Average Scaled Expression
Percent Expressing
ACKR3 CX3CR1 CCR5 CCRL2 CCR1 CXCR6 ACKR1 CXCR4 CCR2 CCR10 CCR7 CCR9 CXCR5 ACKR2 PITPNM3 CCR4 CCR6 CXCR3 CCR3 CCR8
IL9R IL2RB IL18RAP IL12RB1 IL13RA1 IL2RA IL5RA CXCR2 CXCR1 IL10RA IL11RA IL17RB IL4R IL7R IL13RA1 IL1RAP IL17RA IL3RA IL17RC IL17RE IL2RA IL1RL1 IL1R1 IL1R2 IL2RA IL10RB IL12RB2 IL20RB IL18R1 IL6ST
TNFRSF1A NGFR TNFRSF19 TNFRSF1B TNFRSF10B TNFRSF17 TNFRSF12A RELT EDAR TNFRSF10A TNFRSF10D LTBR CD40 EDA2R TNFRSF11A TNFRSF21 TNFRSF13B CD27 TNFRSF14 TNFRSF8 TNFRSF4 FAS TNFRSF18 TNFRSF25 TNFRSF13C
K
CD4+ % Proliferated
% divided cells
No Nivolumab
+ Nivolumab (10 µg/mL)
OKT3 conc (µg/ml)
CD8+ % Proliferated
% divided cells
No Nivolumab
+ Nivolumab (10 µg/mL)
OKT3 conc (µg/ml)
L
CD4
CD8
% T Cells CPI-Bound
Days Since Last Dose of Immunotherapy

## Supplementary Figure S6. CPI-bound T cell transcriptome analysis. Related to Figure 3.

**A.** Transcriptomic pseudotime trajectory analysis of colonic tissue T cells, with density plot visualizing distribution of all CD103$^+$ TRM cells along pseudotime in CPI-colitis, UC and HC samples.

**B.** Expression of *IL7R* along pseudotime in TRMs. The line represents a loess fit of expression against pseudotime.

**C.** Expression of *GZMA* along pseudotime in TRMs. The line represents a loess fit of expression against pseudotime.

**D.** Expression of *ZNF683* along pseudotime in TRMs. The line represents a loess fit of expression against pseudotime.

**E.** PRDM1 TF regulon activity score along pseudotime in TRMs. The line represents a loess fit of TF activity against pseudotime.

**F.** Bar plot comparing the relative fraction of *PDCD1* positive CD8$^+$ T cells from tumor-infiltrating lymphocytes in colorectal (GEO: GSE108989) and liver (GEO: GSE98638) cancers, compared to non-cancer colonic biopsy samples from healthy and inflamed colons.

**G.** Dot plot heatmap visualizing T cell subpopulation specific expression of innate like Killer Ig-Like Receptors and Killer Lectic-like Receptors. The plot visualizes CD4$^+$ T cells, and resident (CD103$^+$) and non-resident (CD103$^-$) CD8$^+$ T cell populations.

**H.** Violin plot visualizing an "exhaustion" gene transcriptional module score distribution across colonic tissue T cell clusters. Transcriptomic score was derived from expression of *PDCD1, CTLA4, HAVCR2, LAG3, LAYN, ENTPD1, TNFRSF9.* Centre bar indicates median value, color indicates mean score.

**I.** Dot plot heatmap visualizing chemokine and cytokine expression profiles associated with CPI-bound and CPI-free CD4$^+$ and CD8$^+$ T cell populations.

**J.** Dot plot heatmap visualizing chemokine and cytokine receptor expression profiles associated with CPI-bound and CPI-free CD4$^+$ and CD8$^+$ T cells.

**K.** Overall CD4$^+$ and CD8$^+$ T cell proliferation rates in Nivolumab treated and non-treated T cells after 5 days of *in vitro* stimulation culture. Proliferated cells are measured by CFSE signal loss. Plot represents mean, error bars standard deviation from mean.

**L.** Plot visualizing fraction of CPI-bound CD4$^+$ and CD8$^+$ T cells in CPI patient samples vs days since last immunotherapy dose was administered in these patients. Dotted line represents a linear data fit. CD4: Pearson's correlation r= -0.880097, p-value = 0.001745. CD8: Pearson's correlation r= -0.8543625, p-value = 0.003356. CD4-CD8: Pearson's correlation r= 0.8599312, p-value = 0.002945.

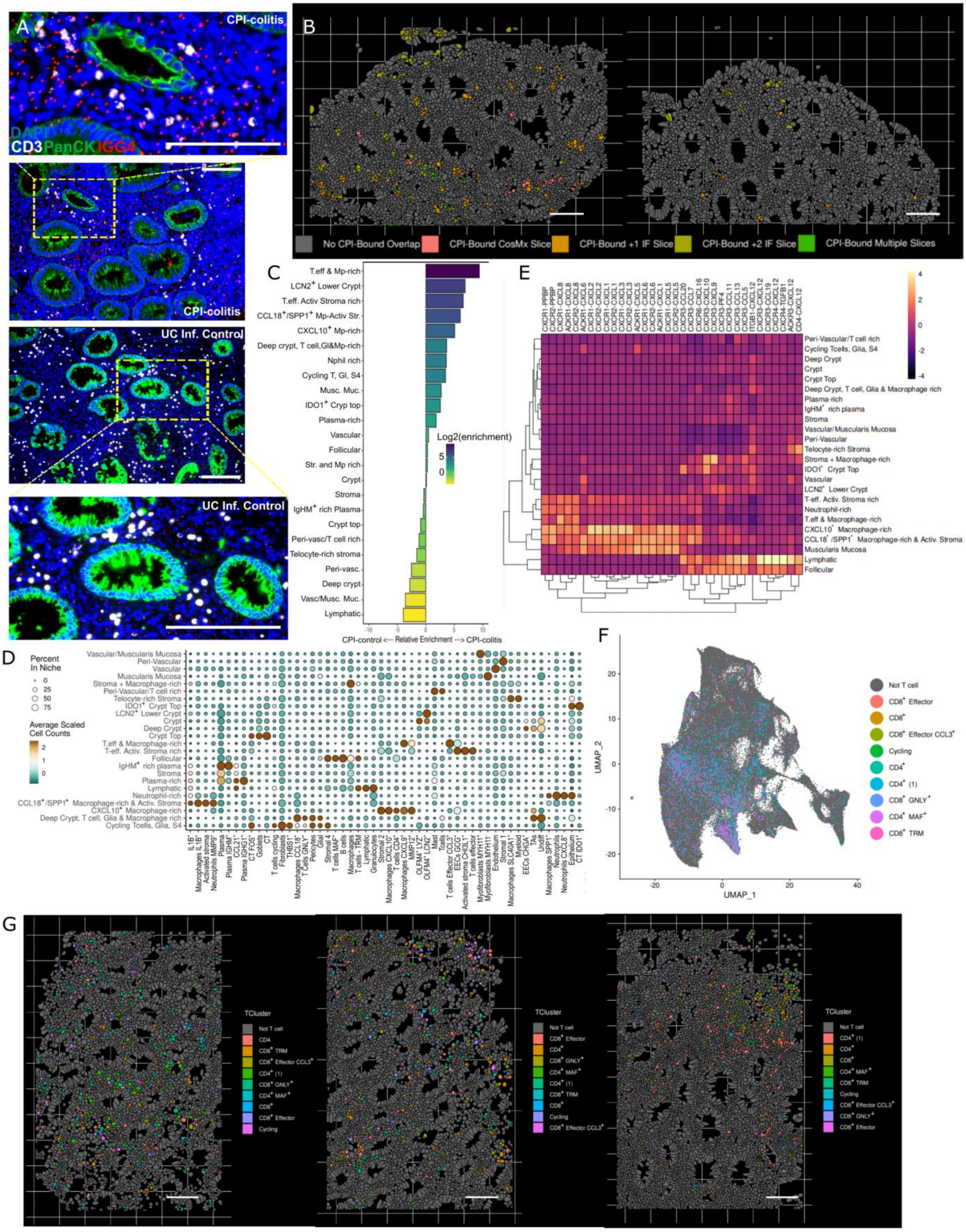
A
CPI-colitis
DAPI
CD3 PanCK IGG4
CPI-colitis
UC Inf. Control
UC Inf. Control
B
No CPI-Bound Overlap
CPI-Bound CosMx Slice
CPI-Bound +1 IF Slice
CPI-Bound +2 IF Slice
CPI-Bound Multiple Slices
C
T.eff & Mp-rich
LCN2+ Lower Crypt
T.eff. Activ Stroma rich
CCL18+/SPP1+ Mp-Activ Str.
CXCL10+ Mp-rich
Deep crypt, T cell,Gl&Mp-rich
Nphil rich
Cycling T, Gl, S4
Musc. Muc.
IDO1+ Cryp top
Plasma-rich
Vascular
Follicular
Str. and Mp rich
Crypt
Stroma
IgHM+ rich Plasma
Crypt top
Peri-vasc/T cell rich
Telocyte-rich stroma
Peri-vasc.
Deep crypt
Vasc/Musc. Muc.
Lymphatic
Log2(enrichment)
CPI-control <--- Relative Enrichment ---> CPI-colitis
E
Peri-Vascular/T cell rich
Cycling Tcells, Glia, S4
Deep Crypt
Crypt
Crypt Top
Deep Crypt, T cell, Glia & Macrophage rich
Plasma-rich
IgHM+ rich plasma
Stroma
Vascular/Muscularis Mucosa
Peri-Vascular
Telocyte-rich Stroma
Stroma + Macrophage-rich
IDO1+ Crypt Top
Vascular
LCN2+ Lower Crypt
T-eff. Activ. Stroma rich
Neutrophil-rich
T.eff & Macrophage-rich
CXCL10+ Macrophage-rich
CCL18+/SPP1+ Macrophage-rich & Activ. Stroma
Muscularis Mucosa
Lymphatic
Follicular
D
Percent In Niche
Average Scaled Cell Counts
F
UMAP_1
UMAP_2
Not T cell
CD8+ Effector
CD8+
CD8+ Effector CCL3+
Cycling
CD4+
CD4+ (1)
CD8+ GNLY+
CD4+ MAF+
CD8+ TRM
G
TCluster
Not T cell
CD4
CD8+ TRM
CD8+ Effector CCL3+
CD4+ (1)
CD8+ GNLY+
CD4+ MAF+
CD8+
CD8+ Effector
Cycling

## Supplementary Figure S7. Spatial analysis of T cells and CPI-bound T cells in CPI-Colitis. Related to Figure 4 and Figure 5.

**A.** Representative field of view image of cell boundary staining in CosMx ST dataset in a CPI-colitis section and an inflamed UC control section. Yellow boxes indicate zoomed in regions shown underneath each image, with CPI-colitis showing a region rich in double positive $CD3^{+}IgG4^{+}$ cells (pink) while in control sections CD3 detection does not co-localize with IgG4. While both types of sections detect background IgG4, however in CPI-colitis double positive cells the IgG4 detection corresponds to the shape of cell boundaries. Scale=100µm.

**B.** Selected fields of view in CosMx ST dataset visualizing the spatial distribution of CPI-bound T cells detected using both tissue morphology markers (CosMx Slice, shown in salmon) and cells overlapping CPI-bound T cells detected by IF in two directly adjacent tissue slices (shown in green/orange). Examples of CPI-bound cells enriched in lamina propria regions and detected across both adjacent IFs and in CosMx slice (left), as well as example of fields of view with limited CPI-bound cells detected (right) are shown.

**C.** Bar plot visualizing relative enrichment of spatial niches detected using CosMx ST data, comparing CPI-colitis and CPI-control samples. Populations are abbreviated as follows: T.eff & Mp-rich - T-effector and Macrophage-rich, T.eff. Activ Stroma rich - T-effector and activated stroma-rich, $CCL18^{+}/SPP1^{+}$ Mp-Activ Str. - $CCL18^{+}/SPP1^{+}$ Macrophage and activated stroma-rich, $CXCL10^{+}$ Mp-rich - $CXCL10^{+}$ Macrophage-rich, Deep crypt,T cell,Gl&Mp-rich - Deep crypt, T cell, Glia and Macrophage-rich, Nphil rich - Neutrophil rich, Cycling T,Gl,S4 - Cycling T cell, Glia and Stromal 4, Musc.Muc. - Muscularis mucosa, Str. and Mp rich - Stroma and Macrophage-rich, Peri-vasc/T cell rich - Peri-vascular and T cell rich, Peri-vasc. - Peri-vascular, Vasc/Musc. Muc. - Vascular/Muscularis mucosa.

**D.** Dot plot heatmap visualizing the relative abundance of cell type clusters across all spatial niche clusters detected using CosMx ST data.

**E.** Heatmap visualizing average, scaled receptor-ligand interaction scores within individual tissue niches for chemokines/chemokine receptor pairs detected in CosMx data.

**F.** UMAP overlay of tissue spatial niche clusters, visualizing the distribution of T cell subpopulations within different spatial domains, highlighting the enrichment of T cells, in particular $MAF^{+}$ cells within lymphoid structures.

**G.** Representative fields of view visualizing the spatial distribution of T cell subpopulations in CosMx ST dataset, showing high prevalence of actively cycling T cells in the lamina propria (left), enrichment of effector $CD8^{+}$ T cells and cycling T cells near regions of tissue damage (middle) and $CD4^{+}$ and $CD4^{+}$ $MAF^{+}$ T cell enrichment near and in lymphoid follicles (right).

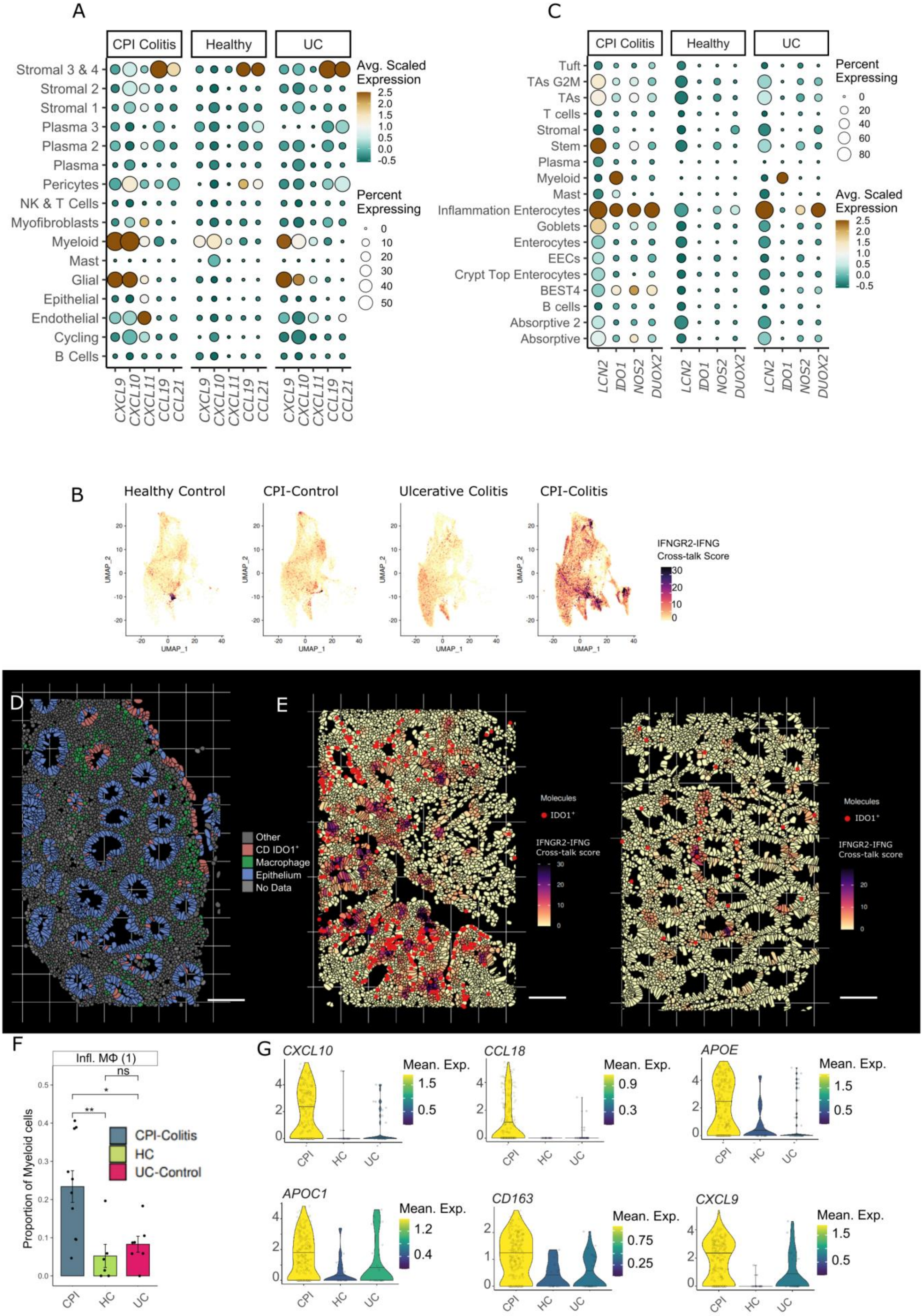

A
CPI Colitis
Healthy
UC
Stromal 3 & 4
Stromal 2
Stromal 1
Plasma 3
Plasma 2
Plasma
Pericytes
NK & T Cells
Myofibroblasts
Myeloid
Mast
Glial
Epithelial
Endothelial
Cycling
B Cells
CXCL9
CXCL10
CXCL11
CCL19
CCL21
Avg. Scaled Expression
2.5
2.0
1.5
1.0
0.5
0.0
-0.5
Percent Expressing
0
10
20
30
40
50
C
CPI Colitis
Healthy
UC
Tuft
TAs G2M
TAs
T cells
Stromal
Stem
Plasma
Myeloid
Mast
Inflammation Enterocytes
Goblets
Enterocytes
EECs
Crypt Top Enterocytes
BEST4
B cells
Absorptive 2
Absorptive
LCN2
IDO1
NOS2
DUOX2
Percent Expressing
0
20
40
60
80
Avg. Scaled Expression
2.5
2.0
1.5
1.0
0.5
0.0
-0.5
B
Healthy Control
CPI-Control
Ulcerative Colitis
CPI-Colitis
UMAP_2
UMAP_1
IFNGR2-IFNG Cross-talk Score
30
20
10
0
D
Other
CD IDO1+
Macrophage
Epithelium
No Data
E
Molecules
IDO1+
IFNGR2-IFNG Cross-talk score
F
Infl. MΦ (1)
ns
*
**
Proportion of Myeloid cells
CPI-Colitis
HC
UC-Control
CPI
HC
UC
G
CXCL10
CCL18
APOE
APOC1
CD163
CXCL9
Mean. Exp.
CPI
HC
UC

## Supplementary Figure S8. Immune responses in tissue niches in CPI-Colitis. Related to Figure 5.

**A.** Dot plot heatmap visualizing differential expression of selected cytokines/chemokines in scRNA-Seq data in immune, stromal and epithelial cell populations.

**B.** Split UMAP overlay showing the enrichment of interferon gamma signaling within specific spatial niches detected in CosMx dataset, as well as overall increase in CPI-colitis samples when compared to controls.

**C.** Dot plot heatmap visualizing differential expression of selected genes in epithelial cells in CPI-colitis and control samples.

**D.** Spatial distribution of crypt top IDO1$^{+}$ cells in a selected CPI-colitis field of view, showing co-localization with macrophages. Scale=100μm

**E.** Spatial distribution of *IDO1* molecule detection visualized over a local interferon gamma cell-cell signaling score in a selected field of view in CPI-colitis (left) and a CPI-control (right) sample. Scale=100μm.

**F.** M2-like inflammatory macrophage cluster abundance changes as a fraction of all myeloid cells in sample in CPI-colitis, UC and healthy control samples, shown as bar plots. Wilcox rank test, p-value < 0.05 ∗; p-value < 0.01 ∗∗; p-value < 0.001∗∗∗; ns = not significant. Error bars represent standard error of the mean (SEM).

**G.** Violin plots visualize the distribution of scRNA-Seq expression of selected M2 marker genes in Inflammatory Macrophage cluster in CPI-colitis, UC and healthy control samples. *CXCL10*: CPI-Colitis vs UC p.val- 0.0005758281, CPI-Colitis vs Health p.val- 3.919621e-21; *CCL18*: CPI-Colitis vs UC p.val - 1.832989e-06, CPI-Colitis vs Health p.val - 4.854261e-12; *APOE*: CPI-Colitis vs UC p.val - 0.004159857, CPI-Colitis vs Health p.val - 7.263763e-05; *APOC1*: CPI-Colitis vs UC p.val - 0.04563992 , CPI-Colitis vs Health p.val - 8.729329e-05 ; *CD163*: CPI-Colitis vs UC p.val - 1.368806e-08 , CPI-Colitis vs Health p.val - 3.073412e-14; *CXCL9*: CPI-Colitis vs UC p.val - 0.01867151 , CPI-Colitis vs Health p.val - 1.36742e-29; Negative binomial test. Centre bar indicates median value, color indicates mean score.

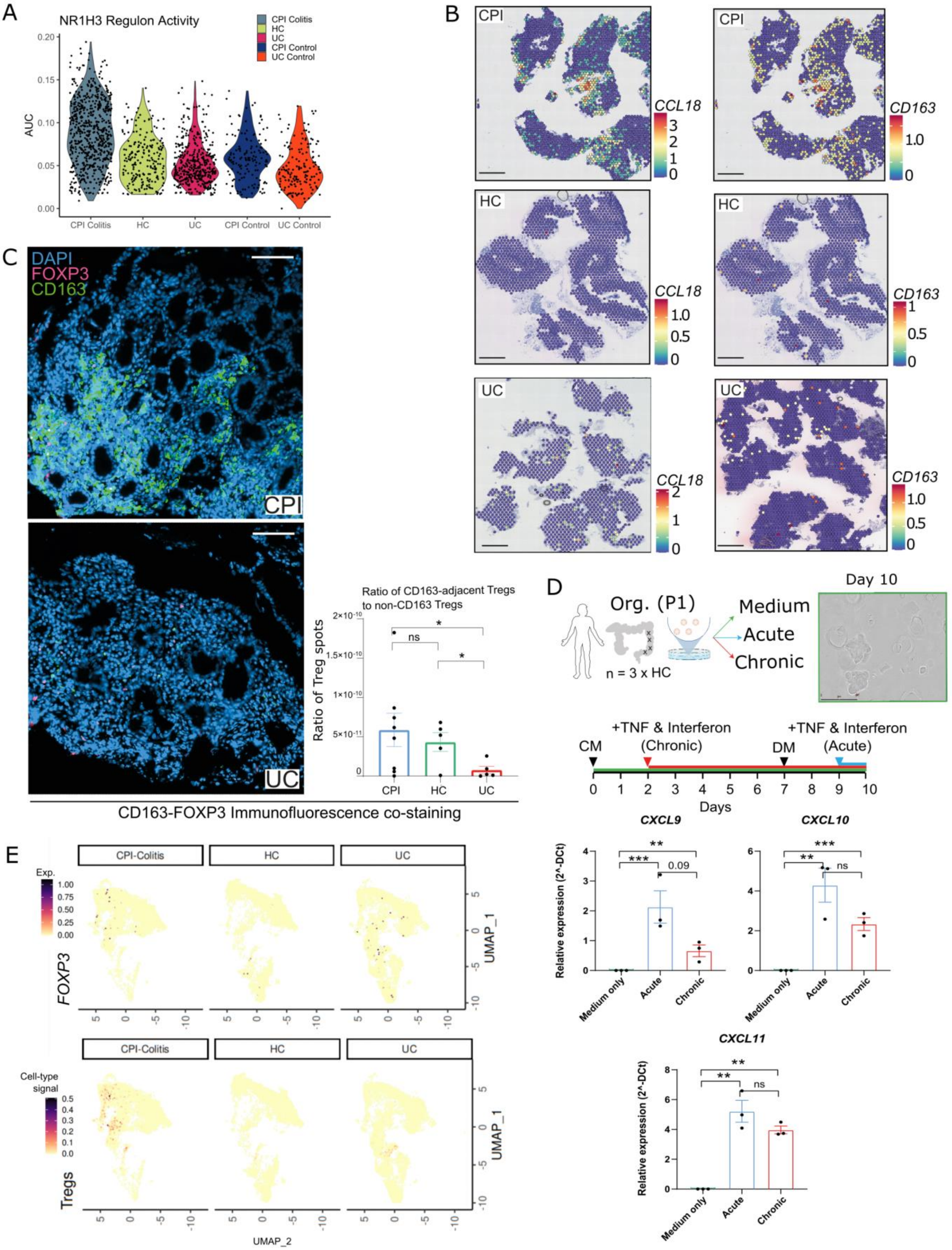
A
NR1H3 Regulon Activity
CPI Colitis
HC
UC
CPI Control
UC Control
AUC
B
CPI
CCL18
CD163
HC
UC
C
DAPI
FOXP3
CD163
CPI
UC
Ratio of CD163-adjacent Tregs to non-CD163 Tregs
Ratio of Treg spots
ns
CD163-FOXP3 Immunofluorescence co-staining
D
Day 10
Org. (P1)
n = 3 x HC
Medium
Acute
Chronic
+TNF & Interferon (Chronic)
+TNF & Interferon (Acute)
CM
DM
Days
CXCL9
CXCL10
CXCL11
Relative expression (2^-DCt)
Medium only
E
CPI-Colitis
Exp.
FOXP3
UMAP_1
UMAP_2
Cell-type signal
Tregs

## Supplementary Figure S9. Macrophage and Treg cell responses in CPI-Colitis. Related to Figure 5.

**A.** Violin plot visualizing NR1H3 TF regulon activity in macrophage cells in scRNA-Seq data in CPI-Colitis, UC and control patient samples. Centre bar indicates median value. CPI-Colitis vs Healthy controls p.val = 1.898957e-27, CPI-Colitis vs UC Inflamed p.val = 1.525041e-57, CPI-Colitis vs CPI-controls p.val= 4.097044e-19. Logistic regression likelihood ratio test.

**B.** Representative Visium ST sections from CPI-colitis, healthy control and UC samples visualizing expression of *CCL18* (left) and *CD163* (right) genes. Scale=1mm

**C.** Representative IF images in CPI-colitis (top) and UC (bottom) in colonic tissue sections show the distribution of CD163 expressing cells (green) in relation to T regulatory cells (FOXP3, red). Bar represents 100 µm. Bar plot (right) shows the number of $FOXP3^{+}$ cells in proximity to $CD163^{+}$ cells in colonic tissue sections as measured using IF across UC, CPI-Colitis and HC samples, normalized to total area. Unpaired non-parametric t-test, mean values are shown, Error bars represent standard error of the mean (SEM). p-value < 0.05 ∗; p-value < 0.001∗∗∗; ns = not significant. n=5-8 samples per condition. Scale=100µm.

**D.** Bar plot (mean +/- standard error of mean) shows the expression of chemokines *CXCL9*, *CXCL10* and *CXCL11* in colonic epithelial organoids stimulated with Interferon-γ and TNF-α in chronic or acute settings, as measured by qPCR. Schematic layout of organoid experiment is depicted above. Ratio paired t-test, ns = not significant, ** p value < 0.01, *** p value < 0.001. Error bars represent standard error of mean limits. n=3 samples. CM=Conditioning medium, DM=Differentiation medium, P1=Passage 1. Scale=500µm.

**E.** UMAP plot visualizing *FOXP3* expression (top) in Visium ST spots in CPI-colitis and control samples. Treg transcriptional signature signal is shown on the bottom. Full UMAP cluster reference is shown in **Figure 1C**.

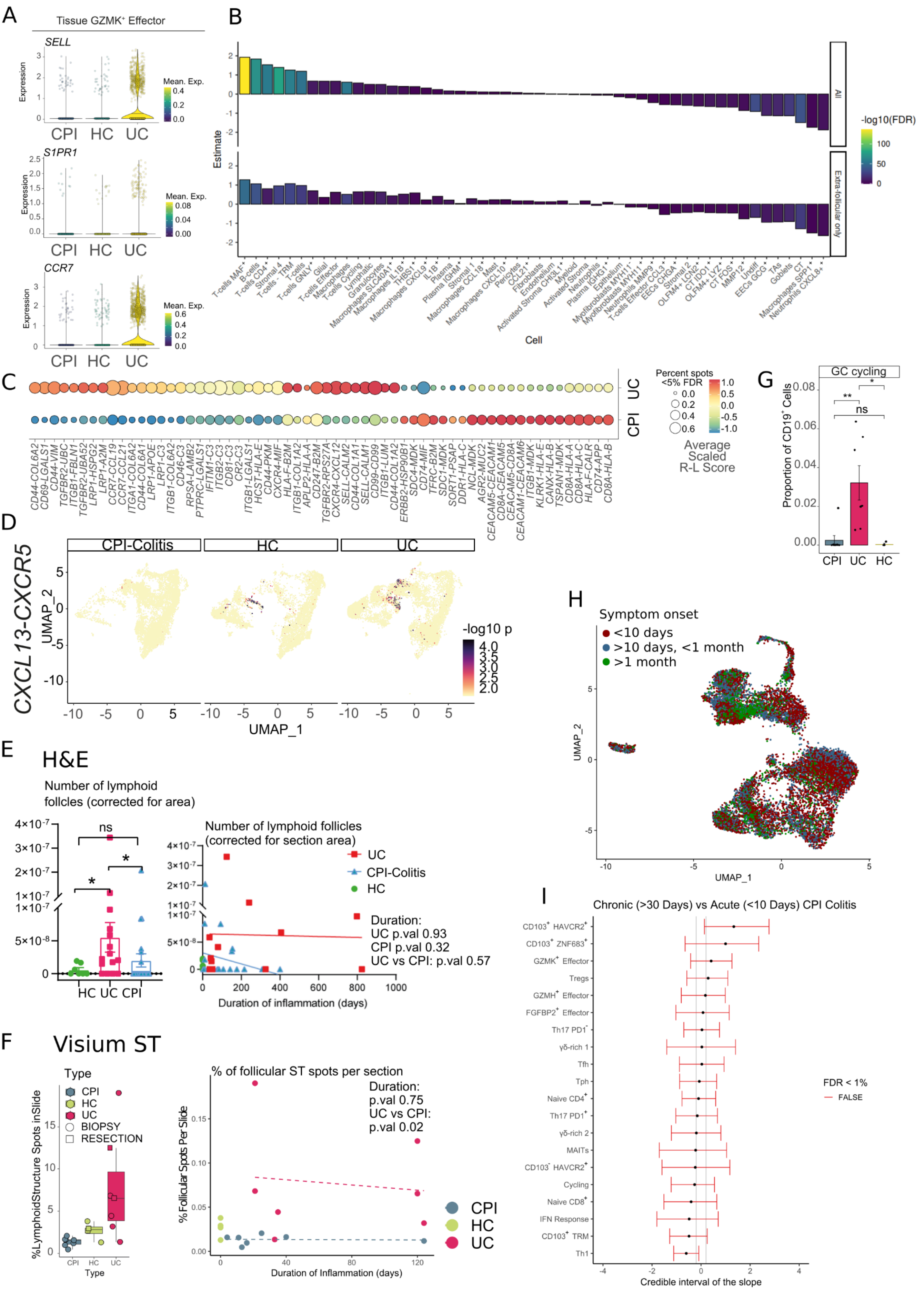
A
Tissue GZMK+ Effector
SELL
S1PR1
CCR7
Expression
Mean. Exp.
CPI HC UC
B
Estimate
All
Extra-follicular only
-log10(FDR)
Cell
C
UC
CPI
Percent spots <5% FDR
Average Scaled R-L Score
G
GC cycling
Proportion of CD19+ Cells
CPI UC HC
D
CXCL13-CXCR5
CPI-Colitis
HC
UC
UMAP_2
UMAP_1
-log10 p
H
Symptom onset
<10 days
>10 days, <1 month
>1 month
UMAP_2
UMAP_1
E
H&E
Number of lymphoid follcles (corrected for area)
HC UC CPI
Number of lymphoid follicles (corrected for section area)
UC
CPI-Colitis
HC
Duration:
UC p.val 0.93
CPI p.val 0.32
UC vs CPI: p.val 0.57
Duration of inflammation (days)
F
Visium ST
Type
CPI
HC
UC
BIOPSY
RESECTION
%LymphoidStructure Spots inSlide
% of follicular ST spots per section
Duration:
p.val 0.75
UC vs CPI:
p.val 0.02
% Follicular Spots Per Slide
Duration of Inflammation (days)
I
Chronic (>30 Days) vs Acute (<10 Days) CPI Colitis
CD103+ HAVCR2+
CD103+ ZNF683+
GZMK+ Effector
Tregs
GZMH+ Effector
FGFBP2+ Effector
Th17 PD1-
γδ-rich 1
Tfh
Tph
Naive CD4+
Th17 PD1+
γδ-rich 2
MAITs
CD103- HAVCR2+
Cycling
Naive CD8+
IFN Response
CD103+ TRM
Th1
FDR < 1%
FALSE
Credible interval of the slope

## Supplementary Figure S10. Follicular responses in CPI-Colitis and UC. Related to Figure 6.

**A.** Violin plot visualizing differential expression of lymph node homing markers in $CD8^+$ effector T cells in CPI-colitis, health and UC. *SELL*: UC vs CPI-Colitis p.val = 0.0001216083. UC vs Healthy Control p.val= 0.003402346. *S1PR1*: UC vs CPI-Colitis p.val = 0.001579474, UC vs Healthy Control p.val= 0.1342413; CCR7: UC vs CPI-Colitis p.val= 8.377306e-05, UC vs Healthy Control p.val= 0.1307333. Negative binomial test. Centre bar indicates median value, color indicates mean score.

**B.** Bar plot showing co-localization enrichment estimates of $MAF^+$ T cells in all tissue cells (top) and only cells outside of follicular regions (bottom). Significant co-localization with B cells is still observed when excluding follicular niches.

**C.** Dot plot heatmap shows selected receptor-ligand pairs detected as both significantly co-localizing within lymphoid structure regions in ST, and differential signaling strength between UC and CPI-colitis slides in lymphoid structure regions. Full results table of receptor-ligand interactions are presented in **Table S3.**

**D.** UMAP overlay of spatial distribution of significant co-localization of *CXCR5-CXCL13* spots in CPI-Colitis, HC and UC slides.

**E**. Bar plot (mean +/- standard error of mean) shows the number of lymphoid follicles detected corrected for overall section area in an independent cohort of H&E tissue sections in healthy, CPI-colitis and UC colonic tissue sections (left) and visualized over the overall duration of inflammation in CPI-colitis and UC (right). Mann-witney U test, ns = not significant, * p value <0.05. Error bars represent mean +/- standard error of mean limits. n=7 HC, n=15 UC and n=23 CPI-colitis samples. (Right): Line represents the best-fit simple linear regression plot of the values, p values as shown in the figure.

**F.** Box plot (the 25th, 50th and 75th percentiles are shown), shows the proportion of follicular/lymphoid structure spots in each ST slide in CPI-colitis and control sections, as a proportion of all QC-passing, tissue covered spots (left), and visualized over the overall duration of inflammation in CPI-colitis and UC patients at the time of sample collection (right). Generalized linear model fit.

**G.** Proportion bar plot visualizes the distribution of germinal center B cell cluster abundance changes between CPI-colitis and control samples. Wilcox rank test. * p.value < 0.05, ** p.value < 0.01, ns not significant. n=4-11.

**H.** UMAP overlay of scRNA-Seq tissue T cells from CPI-Colitis patients only, visualizing cells from patient groups by time between symptom onset and sample collection.

**I.** Abundance analysis of T cell clusters, comparing CPI-colitis patients where samples were collected more than 30 days since symptom onset, and early diagnosis patients (less than 10 days since symptom onset). Over-represented cell subpopulations in late diagnosis samples are indicated by positive slope interval, while under-represented cell subpopulations are negative.

## Supplementary References


94. Capone, A., and Volpe, E. (2020). Transcriptional Regulators of T Helper 17 Cell Differentiation in Health and Autoimmune Diseases. Front. Immunol. *11*. 10.3389/FIMMU.2020.00348.

95. Christie, D., and Zhu, J. (2014). Transcriptional regulatory networks for CD4 T cell differentiation. Curr. Top. Microbiol. Immunol. *381*, 125–172. 10.1007/82_2014_372.

96. Jaeger, N., Gamini, R., Cella, M., Schettini, J.L., Bugatti, M., Zhao, S., Rosadini, C. V., Esaulova, E., Luccia, B. Di, Kinnett, B., et al. (2021). Single-cell analyses of Crohn's disease tissues reveal intestinal intraepithelial T cells heterogeneity and altered subset distributions. Nat. Commun. *12*. 10.1038/S41467-021-22164-6.

97. Martin, J.C., Chang, C., Boschetti, G., Ungaro, R., Giri, M., Grout, J.A., Gettler, K., Chuang, L. shiang, Nayar, S., Greenstein, A.J., et al. (2019). Single-Cell Analysis of Crohn's Disease Lesions Identifies a Pathogenic Cellular Module Associated with Resistance to Anti-TNF Therapy. Cell *178*, 1493-1508.e20. 10.1016/j.cell.2019.08.008.

98. Yamazaki, S., Tanaka, Y., Araki, H., Kohda, A., Sanematsu, F., Arasaki, T., Duan, X., Miura, F., Katagiri, T., Shindo, R., et al. (2017). The AP-1 transcription factor JunB is required for Th17 cell differentiation. Sci. Rep. *7*. 10.1038/S41598-017-17597-3.

99. Evans, C., and Jenner, R. (2013). Transcription factor interplay in T helper cell differentiation. Brief. Funct. Genomics *12*, 499–511. 10.1093/BFGP/ELT025.

100. Oestreich, K., and Weinmann, A. (2012). Transcriptional mechanisms that regulate T helper 1 cell differentiation. Curr. Opin. Immunol. *24*, 191–195. 10.1016/J.COI.2011.12.004.

101. Zhou, L. (2015). How smart can it be: transcriptional regulation of T helper cells by SMAR1. Mucosal Immunol. *8*, 1181–1183. 10.1038/MI.2015.71.

102. Corridoni, D., Antanaviciute, A., Gupta, T., D, F.-C., A, A., M, J., K, P., E, R., S, T., D, I., et al. (2020). Single-cell atlas of colonic CD8 + T cells in ulcerative colitis. Nat. Med. *26*, 1480–1490. 10.1038/S41591-020-1003-4.

103. Egawa, T., Tillman, R.E., Naoe, Y., Taniuchi, I., and Littman, D.R. (2007). The role of the Runx transcription factors in thymocyte differentiation and in homeostasis of naive T cells. J. Exp. Med. *204*, 1945. 10.1084/JEM.20070133.

104. Feng, X., Wang, H., Takata, H., Day, T.J., Willen, J., and Hu, H. (2011). Transcription factor Foxp1 exerts essential cell-intrinsic regulation of the quiescence of naive T cells. Nat. Immunol. *12*, 544. 10.1038/NI.2034.

105. Willinger, T., Freeman, T., Herbert, M., Hasegawa, H., McMichael, A., and Callan, M. (2006). Human naive CD8 T cells down-regulate expression of the WNT pathway transcription factors lymphoid enhancer binding factor 1 and transcription factor 7 (T cell factor-1) following antigen encounter in vitro and in vivo. J. Immunol. *176*, 1439–1446. 10.4049/JIMMUNOL.176.3.1439.

106. Chen, Y., Zander, R., Khatun, A., Schauder, D.M., and Cui, W. (2018). Transcriptional and Epigenetic Regulation of Effector and Memory CD8 T Cell Differentiation. Front. Immunol. *9*, 2826. 10.3389/FIMMU.2018.02826.

107. Kaech, S.M., and Cui, W. (2012). Transcriptional control of effector and memory CD8+ T cell differentiation. Nat. Rev. Immunol. *12*, 749. 10.1038/NRI3307.

108. Behr, F.M., Chuwonpad, A., Stark, R., and Gisbergen, K.P.J.M. van (2018). Armed and Ready: Transcriptional Regulation of Tissue-Resident Memory CD8 T Cells. Front. Immunol. *9*, 1770. 10.3389/FIMMU.2018.01770.

109. Barros-Martins, J., Schmolka, N., Fontinha, D., Miranda, M.P. de, Simas, J.P., Brok, I., Ferreira, C., Veldhoen, M., Silva-Santos, B., and Serre, K. (2016). Effector γδ T Cell Differentiation Relies on Master but Not Auxiliary Th Cell Transcription Factors. J. Immunol. *196*, 3642–3652. 10.4049/JIMMUNOL.1501921.

110. Parker, M.E., and Ciofani, M. (2020). Regulation of γδ T Cell Effector Diversification in the Thymus. Front. Immunol. *11*, 42. 10.3389/FIMMU.2020.00042.

111. Bhullar, J., and Sollars, V.E. (2011). YBX1 expression and function in early hematopoiesis and leukemic cells. Immunogenet. 2011 636 *63*, 337–350. 10.1007/S00251-011-0517-9.

112. Caushi, J.X., Zhang, J., Ji, Z., Vaghasia, A., Zhang, B., Hsiue, E.H.-C., Mog, B.J., Hou, W., Justesen, S., Blosser, R., et al. (2021). Transcriptional programs of neoantigen-specific TIL in anti-PD-1-treated lung cancers. Nat. 2021 5967870 *596*, 126–132. 10.1038/s41586-021-03752-4.

113. Cobaleda, C., Jochum, W., and Busslinger, M. (2007). Conversion of mature B cells into T cells by dedifferentiation to uncommitted progenitors. Nat. 2007 4497161 *449*, 473–477. 10.1038/nature06159.

114. David-Fung, E.-S., Butler, R., Buzi, G., Yui, M.A., Diamond, R.A., Anderson, M.K., Rowen, L., and Rothenberg, E. V. (2009). Transcription factor expression dynamics of early T-lymphocyte specification and commitment. Dev. Biol. *325*, 444. 10.1016/J.YDBIO.2008.10.021.

115. Gegonne, A., Tai, X., Zhang, J., Wu, G., Zhu, J., Yoshimoto, A., Hanson, J., Cultraro, C., Chen, Q.-R., Guinter, T., et al. (2012). The General Transcription Factor TAF7 Is Essential for Embryonic Development but Not Essential for the Survival or Differentiation of Mature T Cells. Mol. Cell. Biol. *32*, 1984. 10.1128/MCB.06305-11.

116. Guo, X., Zhang, Y., Zheng, L., Zheng, C., Song, J., Zhang, Q., Kang, B., Liu, Z., Jin, L., Xing, R., et al. (2018). Global characterization of T cells in non-small-cell lung cancer by single-cell sequencing. Nat. Med. *24*, 978–985. 10.1038/s41591-018-0045-3.

117. Kragten, N.A.M., Behr, F.M., Braga, F.A.V., Remmerswaal, E.B.M., Wesselink, T.H., Oja, A.E., Hombrink, P., Kallies, A., Lier, R.A.W. van, Stark, R., et al. (2018). Blimp-1 induces and Hobit maintains the cytotoxic mediator granzyme B in CD8 T cells. Eur. J. Immunol. *48*, 1644–1662. 10.1002/EJI.201847771.

118. Kumar, B. V., Ma, W., Miron, M., Granot, T., Guyer, R.S., Carpenter, D.J., Senda, T., Sun, X., Ho, S.-H., Lerner, H., et al. (2017). Human tissue-resident memory T cells are defined by core transcriptional and functional signatures in lymphoid and mucosal sites. Cell Rep. *20*, 2921. 10.1016/J.CELREP.2017.08.078.

119. Parish, I.A., and Kaech, S.M. (2009). Diversity in CD8+ T cell differentiation. Curr. Opin. Immunol. *21*, 291. 10.1016/J.COI.2009.05.008.

120. Scott, C.L., and Omilusik, K.D. (2019). ZEBs: Novel Players in Immune Cell Development and Function. Trends Immunol. *40*, 431–446. 10.1016/J.IT.2019.03.001.

121. Vandereyken, M., James, O.J., and Swamy, M. (2020). Mechanisms of activation of innate-like intraepithelial T lymphocytes. Mucosal Immunol. 2020 135 *13*, 721–731. 10.1038/s41385-020-0294-6.

122. Viganò, S., Banga, R., Bellanger, F., Pellaton, C., Farina, A., Comte, D., Harari, A., and Perreau, M. (2014). CD160-Associated CD8 T-Cell Functional Impairment Is Independent of PD-1 Expression. PLoS Pathog. *10*. 10.1371/JOURNAL.PPAT.1004380.

123. Willis, S.N., Tellier, J., Liao, Y., Trezise, S., Light, A., O'Donnell, K., Garrett-Sinha, L.A., Shi, W., Tarlinton, D.M., and Nutt, S.L. (2017). Environmental sensing by mature B cells is controlled by the transcription factors PU.1 and SpiB. Nat. Commun. *8*. 10.1038/S41467-017-01605-1.

124. Xue, H.-H., Jing, X., Bollenbacher-Reilley, J., Zhao, D.-M., Haring, J.S., Yang, B., Liu, C., Bishop, G.A., Harty, J.T., and Leonard, W.J. (2008). Targeting the GA Binding Protein β1L Isoform Does Not Perturb Lymphocyte Development and Function. Mol. Cell. Biol. *28*, 4300. 10.1128/MCB.01855-07.

125. Yao, C., Sun, H.W., Lacey, N.E., Ji, Y., Moseman, E.A., Shih, H.Y., Heuston, E.F., Kirby, M., Anderson, S., Cheng, J., et al. (2019). Single-cell RNA-seq reveals TOX as a key regulator of CD8+ T cell persistence in chronic infection. Nat. Immunol. *20*, 890–901. 10.1038/s41590-019-0403-4.

126. Li, H., van der Leun, A.M., Yofe, I., Lubling, Y., Gelbard-Solodkin, D., van Akkooi, A.C.J., van den Braber, M., Rozeman, E.A., Haanen, J.B.A.G., Blank, C.U., et al. (2019). Dysfunctional CD8 T Cells Form a Proliferative, Dynamically Regulated Compartment within Human Melanoma. Cell *176*, 775-789.e18. 10.1016/j.cell.2018.11.043.

127. Seo, W., Jerin, C., and Nishikawa, H. (2021). Transcriptional regulatory network for the establishment of CD8+ T cell exhaustion. Exp. Mol. Med. *53*, 202. 10.1038/S12276-021-00568-0.

128. Mielke, L.A., Liao, Y., Clemens, E.B., Firth, M.A., Duckworth, B., Huang, Q., Almeida, F.F., Chopin, M., Koay, H.-F., Bell, C.A., et al. (2019). TCF-1 limits the formation of Tc17 cells via repression of the MAF–RORγt axis. J. Exp. Med. *216*, 1682. 10.1084/JEM.20181778.

129. Yamauchi, T., Hoki, T., Oba, T., Jain, V., Chen, H., Attwood, K., Battaglia, S., George, S., Chatta, G., Puzanov, I., et al. (2021). T-cell CX3CR1 expression as a dynamic blood-based biomarker of response to immune checkpoint inhibitors. Nat. Commun. 2021 121 *12*, 1–14. 10.1038/s41467-021-21619-0.

130. Gérart, S., Sibéril, S., Martin, E., Lenoir, C., Aguilar, C., Picard, C., Lantz, O., Fischer, A., and Latour, S. (2013). Human iNKT and MAIT cells exhibit a PLZF-dependent proapoptotic propensity that is counterbalanced by XIAP. Blood *121*, 614–623. 10.1182/BLOOD-2012-09-456095.

131. Leeansyah, E., Svärd, J., Dias, J., Buggert, M., Nyström, J., Quigley, M.F., Moll, M., Sönnerborg, A., Nowak, P., and Sandberg, J.K. (2015). Arming of MAIT Cell Cytolytic Antimicrobial Activity Is Induced by IL-7 and Defective in HIV-1 Infection. PLoS Pathog. *11*. 10.1371/JOURNAL.PPAT.1005072.

132. Arbogast, A., Boutet, S., Phelouzat, M.A., Plastre, O., Quadri, R., and Proust, J.J. (1999). Failure of T Lymphocytes from Elderly Humans to Enter the Cell Cycle Is Associated with Low Cdk6 Activity and Impaired Phosphorylation of Rb Protein. Cell. Immunol. *197*, 46–54. 10.1006/CIMM.1999.1550.

133. Shi, M., Lin, T.H., Appell, K.C., and Berg, L.J. (2009). Cell cycle progression following naïve T cell activation is independent of Jak3/γc cytokine signals. J. Immunol. *183*, 4493. 10.4049/JIMMUNOL.0804339.

134. Koizumi, S., and Ishikawa, H. (2019). Transcriptional Regulation of Differentiation and

Functions of Effector T Regulatory Cells. Cells *8*, 939. 10.3390/CELLS8080939.

135. Shevyrev, D., and Tereshchenko, V. (2019). Treg Heterogeneity, Function, and Homeostasis. Front. Immunol. *10*, 3100. 10.3389/FIMMU.2019.03100.

136. Castro, G., Liu, X., Ngo, K., Leon-Tabaldo, A. De, Zhao, S., Luna-Roman, R., Yu, J., Cao, T., Kuhn, R., Wilkinson, P., et al. (2017). RORγt and RORα signature genes in human Th17 cells. PLoS One *12*. 10.1371/JOURNAL.PONE.0181868.

137. Gavins, F.N.E., and Hickey, M.J. (2012). Annexin A1 and the regulation of innate and adaptive immunity. Front. Immunol. *3*. 10.3389/FIMMU.2012.00354.

138. Ramesh, R., Kozhaya, L., McKevitt, K., Djuretic, I.M., Carlson, T.J., Quintero, M.A., McCauley, J.L., Abreu, M.T., Unutmaz, D., and Sundrud, M.S. (2014). Pro-inflammatory human Th17 cells selectively express P-glycoprotein and are refractory to glucocorticoids. J. Exp. Med. *211*, 89. 10.1084/JEM.20130301.

139. Singh, S.P., Zhang, H.H., Foley, J.F., Hedrick, M.N., and Farber, J.M. (2008). Human T Cells That Are Able to Produce IL-17 Express the Chemokine Receptor CCR6. J. Immunol. *180*, 214–221. 10.4049/JIMMUNOL.180.1.214.

140. Andrew, D.P., Ruffing, N., Kim, C.H., Miao, W., Heath, H., Li, Y., Murphy, K., Campbell, J.J., Butcher, E.C., and Wu, L. (2001). C-C Chemokine Receptor 4 Expression Defines a Major Subset of Circulating Nonintestinal Memory T Cells of Both Th1 and Th2 Potential. J. Immunol. *166*, 103–111. 10.4049/JIMMUNOL.166.1.103.

141. Hudak, S., Hagen, M., Liu, Y., Catron, D., Oldham, E., McEvoy, L.M., and Bowman, E.P. (2002). Immune Surveillance and Effector Functions of CCR10+ Skin Homing T Cells. J. Immunol. *169*, 1189–1196. 10.4049/JIMMUNOL.169.3.1189.

142. Nakatani, T., Kaburagi, Y., Shimada, Y., Inaoki, M., Takehara, K., Mukaida, N., and Sato, S. (2001). CCR4+ memory CD4+ T lymphocytes are increased in peripheral blood and lesional skin from patients with atopic dermatitis. J. Allergy Clin. Immunol. *107*, 353–358. 10.1067/MAI.2001.112601.

143. Soler, D., Humphreys, T.L., Spinola, S.M., and Campbell, J.J. (2003). CCR4 versus CCR10 in human cutaneous TH lymphocyte trafficking. Blood *101*, 1677–1683. 10.1182/BLOOD-2002-07-2348.

144. Batista, N. V., Chang, Y.-H., Chu, K.-L., Wang, K.C., Girard, M., and Watts, T.H. (2020). T Cell–Intrinsic CX3CR1 Marks the Most Differentiated Effector CD4+ T Cells, but Is Largely Dispensable for CD4+ T Cell Responses during Chronic Viral Infection. ImmunoHorizons *4*, 701–712. 10.4049/IMMUNOHORIZONS.2000059.

145. Gerlach, C., Moseman, E.A., Loughhead, S.M., Alvarez, D., Zwijnenburg, A.J., Waanders, L., Garg, R., Torre, J.C. de la, and Andrian, U.H. von (2016). The chemokine receptor CX3CR1 defines three antigen-experienced CD8 T cell subsets with distinct roles in immune surveillance and homeostasis. Immunity *45*, 1270. 10.1016/J.IMMUNI.2016.10.018.

146. Kobayashi, T., Okamoto, S., Iwakami, Y., Nakazawa, A., Hisamatsu, T., Chinen, H., Kamada, N., Imai, T., Goto, H., and Hibi, T. (2007). Exclusive increase of CX3CR1+CD28-CD4+ T cells in inflammatory bowel disease and their recruitment as intraepithelial lymphocytes. Inflamm. Bowel Dis. *13*, 837–846. 10.1002/IBD.20113.

147. Nishimura, M., Umehara, H., Nakayama, T., Yoneda, O., Hieshima, K., Kakizaki, M., Dohmae, N., Yoshie, O., and Imai, T. (2002). Dual Functions of Fractalkine/CX3C Ligand 1 in Trafficking of Perforin+/Granzyme B+ Cytotoxic Effector Lymphocytes That Are Defined by CX3CR1

Expression. J. Immunol. *168*, 6173–6180. 10.4049/JIMMUNOL.168.12.6173.

148. Omilusik, K.D., Best, J.A., Yu, B., Goossens, S., Weidemann, A., Nguyen, J. V., Seuntjens, E., Stryjewska, A., Zweier, C., Roychoudhuri, R., et al. (2015). Transcriptional repressor ZEB2 promotes terminal differentiation of CD8+ effector and memory T cell populations during infection. J. Exp. Med. *212*, 2027. 10.1084/JEM.20150194.

149. Szabo, P.A., Levitin, H.M., Miron, M., Snyder, M.E., Senda, T., Yuan, J., Cheng, Y.L., Bush, E.C., Dogra, P., Thapa, P., et al. (2019). Single-cell transcriptomics of human T cells reveals tissue and activation signatures in health and disease. Nat. Commun. 2019 101 *10*, 1–16. 10.1038/s41467-019-12464-3.

150. Viallard, J.F., Bloch-Michel, C., Neau-Cransac, M., Taupin, J.L., Garrigue, S., Miossec, V., Mercie, P., Pellegrin, J.L., and Moreau, J.F. (2001). HLA-DR expression on lymphocyte subsets as a marker of disease activity in patients with systemic lupus erythematosus. Clin. Exp. Immunol. *125*, 485. 10.1046/J.1365-2249.2001.01623.X.

151. Chiba, A., Murayama, G., and Miyake, S. (2018). Mucosal-Associated Invariant T Cells in Autoimmune Diseases. Front. Immunol. *0*, 1333. 10.3389/FIMMU.2018.01333.

152. Belarif, L., Mary, C., Jacquemont, L., Mai, H. Le, Danger, R., Hervouet, J., Minault, D., Thepenier, V., Nerrière-Daguin, V., Nguyen, E., et al. (2018). IL-7 receptor blockade blunts antigen-specific memory T cell responses and chronic inflammation in primates. Nat. Commun. 2018 91 *9*, 1–13. 10.1038/s41467-018-06804-y.

153. Sakaguchi, S., Yamaguchi, T., Nomura, T., and Ono, M. (2008). Regulatory T Cells and Immune Tolerance. Cell *133*, 775–787. 10.1016/J.CELL.2008.05.009.

154. Fawkner-Corbett, D., Antanaviciute, A., Parikh, K., M, J., AS, G., T, G., N, A., D, K., D, F., E, M., et al. (2021). Spatiotemporal analysis of human intestinal development at single-cell resolution. Cell *184*, 810-826.e23. 10.1016/J.CELL.2020.12.016.

155. Pilling, D., Vakil, V., Cox, N., and Gomer, R.H. (2015). TNF-α–stimulated fibroblasts secrete lumican to promote fibrocyte differentiation. Proc. Natl. Acad. Sci. U. S. A. *112*, 11929. 10.1073/PNAS.1507387112.

156. Shoshkes-Carmel, M., Wang, Y.J., Wangensteen, K.J., Tóth, B., Kondo, A., Massassa, E.E., Itzkovitz, S., and Kaestner, K.H. (2018). Subepithelial telocytes are an important source of Wnts that supports intestinal crypts. Nature *557*, 242. 10.1038/S41586-018-0084-4.

157. Brügger, M.D., Valenta, T., Fazilaty, H., Hausmann, G., and Basler, K. (2020). Distinct populations of crypt-associated fibroblasts act as signaling hubs to control colon homeostasis. PLoS Biol. *18*. 10.1371/JOURNAL.PBIO.3001032.

158. Huan, C., Xu, W., Liu, Y., Ruan, K., Shi, Y., Cheng, H., Zhang, X., Ke, Y., and Zhou, J. (2021). Gremlin2 Activates Fibroblasts to Promote Pulmonary Fibrosis Through the Bone Morphogenic Protein Pathway. Front. Mol. Biosci. *0*, 619. 10.3389/FMOLB.2021.683267.

159. Kosinski, C., Li, V.S.W., Chan, A.S.Y., Zhang, J., Ho, C., Tsui, W.Y., Chan, T.L., Mifflin, R.C., Powell, D.W., Yuen, S.T., et al. (2007). Gene expression patterns of human colon tops and basal crypts and BMP antagonists as intestinal stem cell niche factors. Proc. Natl. Acad. Sci. U. S. A. *104*, 15418. 10.1073/PNAS.0707210104.

160. Martin, J.C., Chang, C., Boschetti, G., R, U., M, G., JA, G., K, G., LS, C., S, N., AJ, G., et al. (2019). Single-Cell Analysis of Crohn's Disease Lesions Identifies a Pathogenic Cellular Module Associated with Resistance to Anti-TNF Therapy. Cell *178*, 1493-1508.e20.

161. Travaglini, K.J., Nabhan, A.N., Penland, L., Sinha, R., Gillich, A., Sit, R. V., Chang, S., Conley, S.D., Mori, Y., Seita, J., et al. (2020). A molecular cell atlas of the human lung from single-cell RNA sequencing. Nat. 2020 5877835 *587*, 619–625. 10.1038/s41586-020-2922-4.

162. Uhlitz, F., Bischoff, P., Peidli, S., Sieber, A., Obermayer, B., Blanc, E., Trinks, A., Lüthen, M., Ruchiy, Y., Sell, T., et al. (2021). Mitogen-activated protein kinase activity drives cell trajectories in colorectal cancer. bioRxiv, 2020.01.10.901579. 10.1101/2020.01.10.901579.

163. Melissari, M.-T., Henriques, A., Tzaferis, C., Prados, A., Sarris, M.E., Chouvardas, P., Grammenoudi, S., Kollias, G., and Koliaraki, V. (2021). Col6a1+/CD201+ mesenchymal cells regulate intestinal morphogenesis and homeostasis. bioRxiv, 2021.02.16.431453. 10.1101/2021.02.16.431453.

164. Popescu, L.M., and Faussone-Pellegrini, M.-S. (2010). TELOCYTES – a case of serendipity: the winding way from Interstitial Cells of Cajal (ICC), via Interstitial Cajal-Like Cells (ICLC) to TELOCYTES. J. Cell. Mol. Med. *14*, 729. 10.1111/J.1582-4934.2010.01059.X.

165. Qi, Z., Li, Y., Zhao, B., Xu, C., Liu, Y., Li, H., Zhang, B., Wang, X., Yang, X., Xie, W., et al. (2017). BMP restricts stemness of intestinal Lgr5+ stem cells by directly suppressing their signature genes. Nat. Commun. *8*. 10.1038/NCOMMS13824.

166. Chen, W., Liu, Y., Liu, W., Zhou, Y., He, H., and Lin, S. (2020). Neuropeptide Y Is an Immunomodulatory Factor: Direct and Indirect. Front. Immunol. *11*. 10.3389/FIMMU.2020.580378.

167. Cox, H.M. (2007). Neuropeptide Y receptors; antisecretory control of intestinal epithelial function. Auton. Neurosci. Basic Clin. *133*, 76–85. 10.1016/J.AUTNEU.2006.10.005.

168. El-Salhy, M., and Hausken, T. (2016). The role of the neuropeptide Y (NPY) family in the pathophysiology of inflammatory bowel disease (IBD). Neuropeptides *55*, 137–144. 10.1016/J.NPEP.2015.09.005.

169. Harnack, C., Berger, H., Antanaviciute, A., Vidal, R., Sauer, S., Simmons, A., Meyer, T.F., and Sigal, M. (2019). R-spondin 3 promotes stem cell recovery and epithelial regeneration in the colon. Nat. Commun. 2019 101 *10*, 1–15. 10.1038/s41467-019-12349-5.

170. Gautier, E.L., Shay, T., Miller, J., Greter, M., Jakubzick, C., Ivanov, S., Helft, J., Chow, A., Elpek, K.G., Gordonov, S., et al. (2012). Gene expression profiles and transcriptional regulatory pathways underlying mouse tissue macrophage identity and diversity. Nat. Immunol. *13*, 1118. 10.1038/NI.2419.

171. Villar, J., and Segura, E. (2020). Decoding the Heterogeneity of Human Dendritic Cell Subsets. Trends Immunol. *41*, 1062–1071. 10.1016/J.IT.2020.10.002.

172. I, S., C, W., SA, J., KS, C., C, T., MC, W., J, H., and FE, L. (2019). Challenges and Opportunities for Consistent Classification of Human B Cell and Plasma Cell Populations. Front. Immunol. *10*. 10.3389/FIMMU.2019.02458.

173. da Silva, F.A.R., Pascoal, L.B., Dotti, I., Setsuko Ayrizono, M. de L., Aguilar, D., Rodrigues, B.L., Arroyes, M., Ferrer-Picon, E., Milanski, M., Velloso, L.A., et al. (2020). Whole transcriptional analysis identifies markers of B, T and plasma cell signaling pathways in the mesenteric adipose tissue associated with Crohn's disease. J. Transl. Med. 2020 181 *18*, 1–14. 10.1186/S12967-020-02220-3.

174. Stewart, A., Ng, J.C.-F., Wallis, G., Tsioligka, V., Fraternali, F., and Dunn-Walters, D.K. (2021). Single-Cell Transcriptomic Analyses Define Distinct Peripheral B Cell Subsets and Discrete Development Pathways. Front. Immunol. *0*, 743. 10.3389/FIMMU.2021.602539.
